# Comparative Real-Time Kinetics of Ligand–Receptor Interactions Using Immobilization-Based Sensing Readouts

**DOI:** 10.1021/acs.analchem.6c02900

**Published:** 2026-07-09

**Authors:** Yazheng Wang, Yalun Wu, Lauren A. Mayse, Danny Capucilli, Aaron J. Wolfe, Liviu Movileanu

**Affiliations:** † Department of Physics, 2029 Syracuse University, 201 Physics Building, Syracuse, New York, New York 13244, United States; ‡ Department of Biomedical and Chemical Engineering, Syracuse University, 329 Link Hall, Syracuse, New York, New York 13244, United States; § 558832 Ichor Life Sciences Inc, 831 James Street, Syracuse, New York, New York 13203, United States; ∥ Department of Chemistry, College of Environmental Science and ForestryState, 14797 University of New York, 1 Forestry Dr., Syracuse, New York, New York 13210, United States; ⊥ The BioInspired Institute, Syracuse University, Syracuse, New York, New York 13244, United States; # Department of Biology, Syracuse University, 114 Life Sciences Complex, Syracuse, New York, New York 13244, United States

## Abstract

Interactions between receptor tyrosine kinases and their specific growth factor ligands are crucial for cell signaling. Although much research has focused on these processes, quantitative attention to their earliest kinetics has been limited. Here, we used two immobilization-based sensing methods, biolayer interferometry (BLI) and surface plasmon resonance (SPR), to analyze the real-time binding kinetics of three high-affinity ligands with the full extracellular domain of various epidermal growth factor receptor (EGFR) isoforms. We observed that BLI measurements show both fast and slow dissociation phases, regardless of the EGFR isoform. SPR experiments, despite using a different detection readout, confirm the existence of two binding substates, indicating that this is a common feature of ligand-EGFR interactions. Furthermore, our data systematically demonstrate that the type of immobilization-based sensing technique influences not only the magnitude of kinetic and affinity parameters but also the relative interactions among different ligands and EGFR isoforms. Using these methods, we also found that glycan side chains at position N151 within the canonical ligand-binding site do not affect overall interactions with the three common high-affinity growth factors. Finally, we show that an extensively deglycosylated EGFR isoform binds all tested ligands with significantly lower affinity. Our approach could be applied to other ligand–receptor systems to directly evaluate how specific posttranslational modifications impact their interactions.

## Graphical abstract

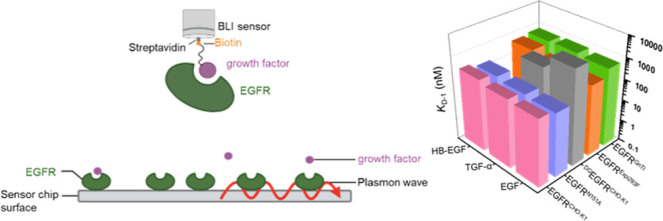

## Introduction

The epidermal growth factor receptor (EGFR) is a critical protein within the receptor tyrosine kinase (RTK) family.^1^ EGFR plays a focal role in a key regulatory signaling pathway, transmitting growth signals into cells and directly influencing their development, survival, and proliferation.^2,3^ It is also upregulated in several cancers, making it a strategic therapeutic target.^4–8^ EGFR is a single-chain transmembrane glycoprotein that consists of an extracellular ligand-binding domain (ectodomain, ECD), a single-pass transmembrane domain, and an intracellular kinase-containing domain. The receptor is activated by direct binding of a soluble growth factor (GF) polypeptide ligand to its extracellular ECD, triggering dimerization via receptor–receptor interactions.^9–11^ In addition, EGFR selectively interacts with and preferentially responds to seven GFs^12–14^ with varying binding affinities. These include the epidermal growth factor (EGF), transforming growth factor-alpha (TGF-α), heparin-binding EGF-like growth factor (HB-EGF), betacellulin (BTC), amphiregulin (AREG), epiregulin (EREG), and epigen (EPGN). EGF, TGF-α, HB-EGF, and BTC are high-affinity GF ligands, while AREG, EREG, and EPGN are low-affinity GF ligands.^12,13^

Ligand-binding to the EGFR ECD produces conformational alterations in the intracellular domain of the monomeric form of the full-length receptor.^15^ Hence, there is a coupling between the extracellular binding interactions and the intracellular conformational modifications of EGFR. Moreover, the kinetics and strength of ligand–receptor interactions serve as a fingerprint of subsequent dimerization stability and signaling output dynamics. For example, there is strong experimental evidence that different low- and high-affinity GFs exert distinct effects on dimer stability and the trajectory of the EGFR signaling pathway at the cellular level.^16–19^ The low-affinity EREG and EPGN ligands trigger significantly less stable EGFR dimers yet produce a prolonged EGFR signaling effect compared with the canonical EGF ligand.^20^

The crucial role of GF-EGFR ECD interactions under physiological conditions is further amplified by oncogenic mutations in the receptor’s extracellular domain (e.g., in glioblastoma).^6^ For example, Macdonald-Oberman and Pike (2024)^21^ demonstrated that three common glioblastoma mutants, R84K, A265 V, and G574 V, significantly increase the binding affinity of each GF to the receptor’s extracellular domain. Molecular dynamics (MD) simulations have also been used to gain mechanistic insights into the ligand–receptor interactions.^22–24^ A special emphasis in these computational studies was on studying the effect of glycosylation at Asn sites (N-glycosylation) on the structure, stability, and dynamics of the receptor in its monomeric and dimeric forms.^25–27^ These studies agree that the N-glycosylation is significant in stabilizing the overall ECD structure of EGFR and maintaining its function. Although many reports in the literature over the years address ligand–receptor binding kinetics, dynamics, and stoichiometry, both in vitro and in living cells, the details of these processes vary substantially from case to case. These variations result from subtle differences in experimental conditions, approaches, readout signals, receptor isoforms, and host expression systems used across these studies.^7,16,28,29^

In this study, we utilized two label-free, immobilization-based biosensing approaches using biolayer interferometry (BLI)^30,31^ and surface plasmon resonance (SPR)^32,33^ to examine the different effects of reversible interactions between high-affinity growth factors (GFs), such as EGF, TGF-α, and HB-EGF, and several soluble EGFR ECD isoforms (Figure 1; Supporting Information Tables S1–S6 and Figures S1–S3).

**Figure 1:**
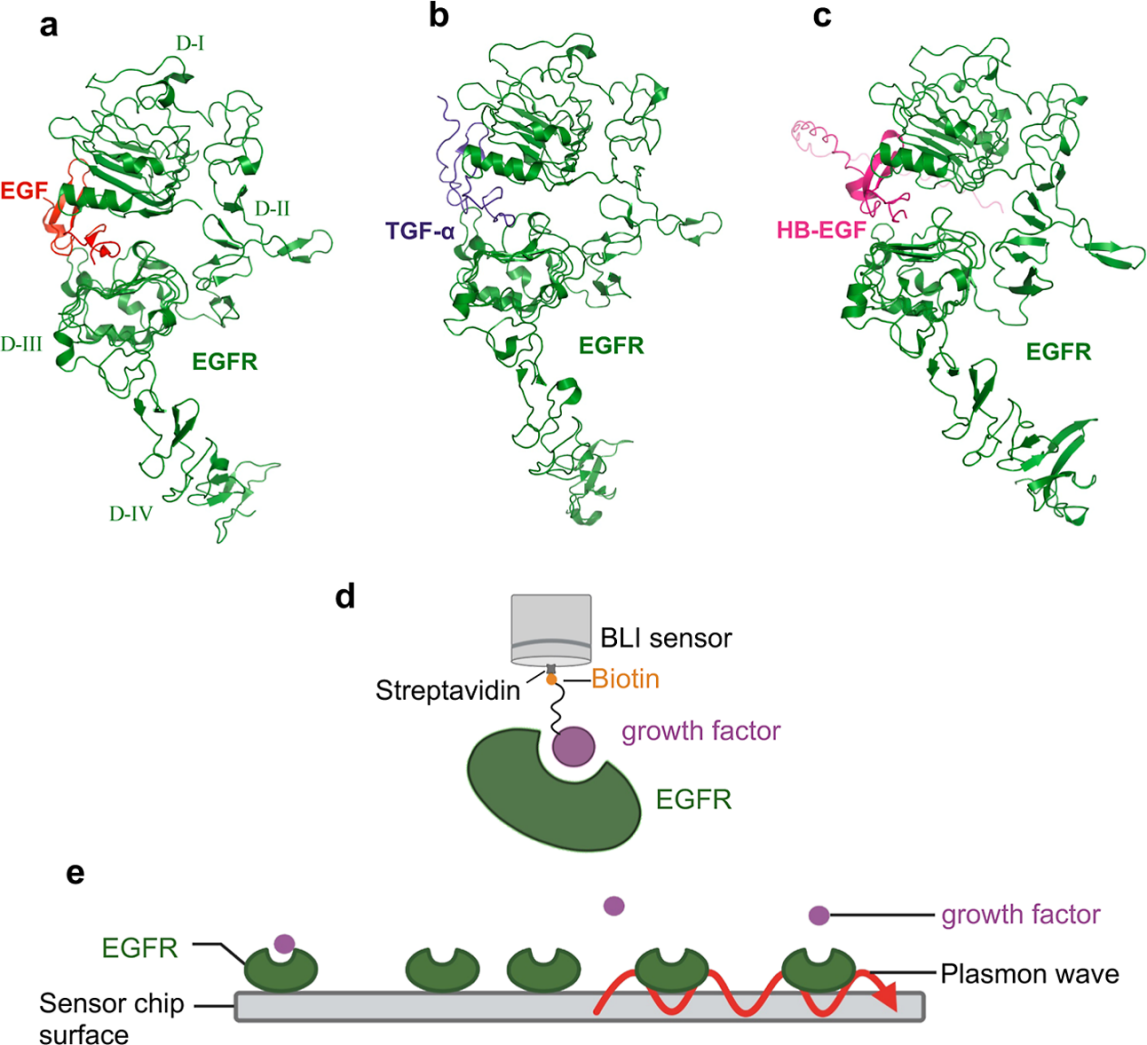
Three-dimensional structures of the GF-EGFR ECD complexes. (a) The molecular model of the EGF-EGFR ECD complex (8HGS.pdb).^34^ (b) The molecular model of the TGFα-EGFR ECD complex (7SZ7.pdb).^17^ (c) The AlphaFold3 prediction of the molecular model of the HB-EGF-EGFR ECD complex.^35^ (d) An immobilized biotinylated GF ligand (purple) is loaded onto the streptavidin-coated BLI sensor (gray), while EGFR (green) is in the well. (e) EGFR (green) is immobilized on the CM5 SPR chip via amine coupling, while the GF ligand (purple) remains free in solution.

We systematically determined the association and dissociation rate constants, as well as the equilibrium dissociation constants for all cases, revealing quantitative differences in the measurements obtained with these two methods. Our findings unambiguously indicate that the glycan side chains at position N151 within the ligand binding site did not affect the strong affinity of each GF. Furthermore, we present compelling experimental evidence that an extensively deglycosylated EGFR ECD isoform shows a markedly reduced binding affinity to all GF ligands tested in this work.

## Experimental Section

### Reagents and Proteins

Recombinant human epidermal growth factor (EGF) was obtained from GoldBio (St. Louis, MO; catalog #1150-04-1000). Human transforming growth factor-alpha (TGF-α) and human heparin-binding EGF-like growth factor (HB-EGF) were obtained from Thermo Fisher Scientific (Waltham, MA; catalog #100-16A-250UG and #100-47-250UG, respectively). Tobacco Etch Virus (TEV) protease was obtained from GenScript (Piscataway, NJ; catalog #Z03030-10K). All other chemical reagents were obtained from Sigma-Aldrich (St. Louis, MO).

### The Design of Genes and Expression Constructs

The ompa3-pngasef-tev-his6 gene was purchased from Addgene (Addgene plasmid #114274). This gene was used to produce PNGase, which was used to remove N-glycans from EGFR ECD isoforms. The egfr^n151a^ gene was generated using the egfr gene and employing site-directed mutagenesis with the Q5 mutagenesis kit (New England Biolabs, Ipswich, MA).^36^ All plasmid sequences were checked through whole-plasmid DNA sequencing by MCLab (San Francisco, CA). All amino acid sequences of the proteins used in this study are provided in Supporting Information Tables S1–S4.

### Protein Expression and Purification

The ectodomains (ECDs) of the epidermal growth factor receptor (EGFR) isoforms were expressed in Expi293F cells (Thermo Fisher Scientific; catalog A14527), Expi293F GnTI cells (Thermo Fisher Scientific; catalog A39240), or CHO-K1 cells (ATCC, Manassas, VA; catalog CCL-61) using poly ethylenimine (PEI) mediated transfection. The cells were cultured in 1 L batches for 5 days in Dynamis Growth Medium (Gibco, Thermo Fisher Scientific, Pittsburgh, PA), during which the targeted proteins were secreted into the culture medium. The supernatant was treated with 5 mL of 1 M Tris–HCl, pH 8.0, and 125 μL of CaCl_2_, then loaded onto a 1 mL HisTrap HP immobilized metal-affinity column (GE Healthcare Life Sciences, Pittsburgh, PA). This column was pre-equilibrated with a buffer containing 50 mM Tris–HCl, 500 mM NaCl, 10% glycerol, 10 mM imidazole, and pH 8.0. The bound protein was eluted using a 100 mL linear gradient from 0% to 100% of an elution buffer containing 50 mM Tris–HCl, 500 mM NaCl, 10% glycerol, 500 mM imidazole, pH 8.0. The peak fractions containing the target protein were assessed for purity by SDS-PAGE. Pooled fractions were dialyzed overnight in 2 L of 20 mM sodium phosphate, 150 mM NaCl, pH 7.5. The protein was further purified using a Superdex 200 pg SEC column (HiLoad, Cytiva, Marlborough, MA). The protein sample was concentrated using a 10 kDa molecular weight cutoff concentrator and flash-frozen in 100 μL aliquots.

The deglycosylation enzyme PNGase F was expressed using *E. coli* BL21­(DE3) cell line (New England Biolabs, Ipswich, MA). All transformed cells were grown in Luria–Bertani medium containing 100 μg/mL ampicillin at 37 °C until the OD_600_ reached 0.5, then induced with 0.5 mM isopropyl β-D-1-thiogalactopyranoside (IPTG) and grown for 16 h at 25 °C. The cells were harvested by centrifugation at 3500 × *g* for 60 min at 4 °C. The cell pellet was resuspended in lysis buffer containing 150 mM KCl, 50 mM Tris, 5 mM ethylenediaminetetraacetic acid (EDTA), pH 7.5. We utilized a microfluidizer (model 110 L; Microfluidics, Newton, MA) to lyse the cells, followed by their centrifugation at 108,000 × *g* at 4 °C for 30 min. Then, the supernatant was collected and filtered using a 0.22 μm filter. Further purification was performed using fast protein liquid chromatography (FPLC) with a next-generation chromatography (NGC) Quest 10 Plus system (Bio-Rad, Hercules, CA). This FPLC system was equipped with an immobilized metal affinity chromatography (IMAC) column (5 mL EconoFit Profinity IMAC; Bio-Rad, Hercules, CA). The protein was eluted using a 10-column linear imidazole gradient in a buffer containing 150 mM KCl, 50 mM Tris–HCl, 500 mM imidazole, and 5 mM EDTA, pH 7.5. The peak fractions containing the targeted recombination protein were collected and dialyzed using a 14 kDa molecular weight cutoff bag in 1 L of dialysis buffer containing 150 mM KCl, 50 mM Tris, 5 mM EDTA, pH 7.5 at 4 °C for 24 h. We then used the TEV protease digestion to remove the hexahistidine tag and incubated the protein samples at 30 °C for 2 h. The protease tag was removed using the NGC Quest 10 Plus system equipped with an IMAC column. The protein was concentrated using 10 kDa molecular weight cutoff concentrators (Corning, Glendale, AZ), then resuspended in a storage buffer containing 50 mM KCl, 20 mM Tris, 5 mM EDTA, and 50% (v/v) glycerol, pH 7.5, and stored at −20 °C. The purity of PNGase F was verified by SDS-PAGE. The protein concentration was determined by measuring the absorbance at 280 nm using a SpectraMax i3 plate reader (Molecular Devices, San Jose, CA).

### Extensive N-Deglycosylation of the EGFR ECD

PNGase F was added to the EGFR solution, and the mixture was incubated at 37 °C for 24 h. The reaction was stopped by removing the enzyme using an IMAC column. The deglycosylated EGFR protein was eluted and dialyzed in a 14 kDa molecular weight cutoff bag against 1 L of dialysis buffer containing 150 mM KCl, 20 mM Tris–HCl, pH 7.5, at 4 °C for 24 h. The protein was concentrated using a 10 kDa molecular weight cutoff concentrator (Corning, Glendale, AZ). Protein purity was assessed by absorbance scanning from 260 to 320 nm. The degree of N-deglycosylation of PNGase F-treated EGFR ECD was evaluated by time-dependent SDS-PAGE.

### Biolayer Interferometry Determinations

Biolayer interferometry (BLI) experiments were performed using an Octet Red384 platform (FortéBio, Fremont, CA).^37,38^ The GF ligand was biotinylated at its N-terminus through a flexible spacer to enable immobilization onto streptavidin-coated BLI sensors.^39,40^ The biotinylated spacer, EZ-Link NHS-(PEG)_12_-Biotin (Thermo Scientific, Rockford, IL), was chemically attached to the GF ligand using NHS-ester–mediated amine coupling chemistry.^41^ Excess unreacted spacer was removed using a PD-10 desalting column (Cytiva, Marlborough, MA). All BLI measurements were conducted in buffer containing 150 mM KCl, 20 mM Tris–HCl, 1 mg/mL bovine serum albumin (BSA), and 0.005% (v/v) polysorbate 20 (Tween-20) at pH 7.5. Streptavidin sensors were presoaked in the running buffer for 15 min, then loaded with 50 nM biotinylated GF for 5 min. During the association phase, sensors were immersed in wells containing EGFR ECD, whereas during the dissociation phase, sensors were transferred to wells with EGFR ECD-free buffer. Reference sensors lacking immobilized growth GF were run in parallel, and their signals were subtracted to correct the sensorgram baseline. All experiments were performed in triplicate. Binding curves were analyzed and fitted using Octet Data Analysis software (FortéBio). The BLI data were analyzed using heterogeneous 2:1 and homogeneous 1:1 ligand-binding kinetic models (see below). The heterogeneous kinetic model assumes two independent binding events, each with distinct association and dissociation rate constants. The total binding response was modeled as the sum of the contributions from each population.^42,43^ All measurements were performed at 24 °C.

### Surface Plasmon Resonance Measurements

All surface plasmon resonance (SPR) experiments^32,44^ were conducted on a Cytiva Biacore 8K Plus (Cytiva Life Sciences, Marlborough, MA).^31^ All buffers and dilutions were prepared in-house using ultrapure water from an IQ 7000 Milli-Q system (Millipore Sigma, Burlington, MA). EGFR ECD proteins were immobilized onto the active flow cell of each channel of a Cytiva Series S Sensor Chip CM5 (Cytiva Life Sciences) according to the following protocol. A CM5 chip was inserted into the instrument and equilibrated for 1 h at 25 °C in PBS-P and in a running buffer consisting of 20 mM sodium phosphate, 137 mM NaCl, 2.7 mM KCl, and 0.05% (v/v) Tween 20, pH 7.4. The chip surface was activated with a 420 s injection of a 1:1 mixture of *N*-hydroxysuccinimide (NHS) and 1-ethyl-3-(3-(dimethylamino)­propyl) carbodiimide (EDC) (Cytiva Amine Coupling Kit, Cytiva Life Sciences) at 10 μL/min across both active and reference flow cells. This activation was followed by washing the microfluidics with 1 M ethanolamine-HCl at pH 8.0.

Following activation, GF, prepared in 10 mM sodium acetate, 50 mM NaCl, pH 4.0, was injected across the active flow cell for 270 s at 5 μL/min. Following EGFR ECD immobilization, both active and passive flow cells were chemically deactivated by a 420 s injection of 1 M ethanolamine-HCl, pH 8.0, at 10 μL/min. All analytes were prepared in the same manner as for the BLI assay. Multicycle kinetic analyses were conducted at a flow-cell and sample-compartment temperature of 25 °C in a running buffer composed of 20 mM Tris–HCl, 150 mM KCl, 1% (w/v) BSA, and 0.05% (v/v) Tween 20, pH 7.5. All GF injections consisted of a 2-fold, 12-point dilution series with a 60 s association time, a 300 s dissociation time, and a flow rate of 50 μL/min. Before curve fitting, all data from the active flow cell in each channel were double referenced to both the appropriate buffer blanks and the reference flow cell. For all data, the equilibrium dissociation constant, *K*_D_, was calculated using heterogeneous (2:1) and homogeneous (1:1) ligand-binding kinetics models (see below). All interactions were determined independently in triplicate.

### Data Analysis of the Kinetics of GF-EGFR ECD Interactions

We assume that there is no exchange between the R_1_ and R_2_ substate sensor responses. Hence, the two binding reactions and the conservation of mass in the two parallel reactions yield the two ordinary differential equations that describe the response contributions *R*_1_(t) and *R*_2_(t)^45^

1 $$\frac{dR_{1}\left(t\right)}{dt}=k_{on-1}\left[C\right]\left(R_{\max-1}-R_{1}\left(t\right)\right)-k_{off-1}R_{1}\left(t\right)$$

2 $$\frac{dR_{2}\left(t\right)}{dt}=k_{on-2}\left[C\right]\left(R_{\max-2}-R_{2}\left(t\right)\right)-k_{off-2}R_{2}\left(t\right)$$

where *k*_on‑1_ and *k*_on‑2_ are the rate constants of association. Here, *k*_off‑1_ and *k*_off‑2_ are the rate constants of dissociation. *R*_max‑1_ and *R*_max‑2_ indicate the maximum binding responses corresponding to the two substates, and [*C*] is the analyte concentration.

The total measured response is given by

3 $$R\left(t\right)=R_{1}\left(t\right)+R_{2}\left(t\right)$$

and

4 $$R_{\max}=R_{\max-1}+R_{\max-2}$$

During the dissociation phase, when the sensor was transferred to an EGFR-free buffer, the response exhibited a biexponential decay.

5 $$R\left(t\right)=R_{1}\left(t_{0}\right)e^{-k_{off-1}t}+R_{2}\left(t_{0}\right)e^{-k_{off-2}t}$$

where *R*_1_(*t*_0_) and *R*_1_(*t*_0_)­are the responses at the start of dissociation for each binding mode. The corresponding equilibrium dissociation constants were calculated as

6 $$K_{D-1}=\frac{k_{off-1}}{k_{on-1}}$$

7 $$K_{D-2}=\frac{k_{off-2}}{k_{on-2}}$$

The 1:1 binding model assumes that a single analyte molecule binds reversibly to a single immobilized ligand site, characterized by a single set of association (*k*_on_) and dissociation (*k*_off_) rate constants. During the association phase, the time-dependent response *R*(t) is governed by

$$\frac{dR\left(t\right)}{dt}=k_{on}C\left(R_{\max}-R\left(t\right)\right)-k_{off}R\left(t\right)$$ 8

where *R*(t) means observed sensor response at time *t*, *R*_max_ is the maximum binding response, and *C* is the analyte concentration. The solution for *R*(*t*) during association can be presented as^46,47^

$$R\left(t\right)=R_{\max}\frac{\left(k_{on}C\right)}{\left(k_{on}C+k_{off}\right)}\left(1-e^{-\left(k_{on}C+k_{off}\right)t}\right)$$ 9

During the dissociation phase, when the sensor was transferred to EGFR-free buffer

$$\frac{dR\left(t\right)}{dt}=-k_{off}R\left(t\right)$$ 10

The equilibrium dissociation constant was calculated as

11 $$K_{D}=\frac{k_{off}}{k_{on}}$$

## Results and Discussion

### Two Real-Time Kinetic Methods to Determine GF-EGFR ECD Interactions

Our main biosensing approach involved attaching each high-affinity GF ligand to the BLI sensor surface via biotin–streptavidin chemistry (Figure 1), with EGFR ECD isoforms added to the wells (experimental section). However, initial tests with a flexible linker approximately 3 nm long failed to elicit any BLI response, likely due to steric hindrance from the GF attached to the sensor surface. Consequently, we increased the linker length to 5.6 nm, which yielded detectable BLI responses during both the association and dissociation phases (Supporting Information Figures S4 and S5). Importantly, the longer linker not only helped generate a BLI signal but also significantly reduced the entropic repulsion of the bound EGFR ECD isoform from the sensor surface.^48^ Further, an alternative approach to benchmark our kinetic determinations using a similar readout is to use BLI to study GF-EGFR ECD interactions with the GF ligand added to the wells. Unfortunately, we observed no satisfactory BLI response because the GF molecular masses are below the method’s minimum detection limit for these analytes (Supporting Information Table S2 and Figure S6).^30^ Furthermore, an SPR-based approach using NHS-ester-mediated amine-reactive cross-linker chemistry, in which EGFR ECD isoforms were covalently attached to the sensor surface (Experimental Section), was introduced to study the interaction.^31,41^ In this way, we opportunistically utilized two related but complementary real-time, label-free, and immobilization-based approaches to determine binding kinetics when either reactant is attached to the sensor surface. The association and dissociation kinetics were first measured using wild-type EGFR ECD expressed in CHO-K1 cell lines (EGFR^CHO–K1^), since this EGFR ECD isoform has been well characterized for mapping glycan side chains at the asparagine sites (N-sites, Supporting Information Figure S2 and Table S6).^49^

Representative sets of BLI and SPR curves obtained with EGF, TGF-α, and HB-EGF are shown in Figure 2, with the left panel (BLI) and the right panel (SPR) displaying the respective data. In all cases, we observed a fast-response BLI dissociation phase, followed by a slower one. This finding suggests that a single-exponential fit cannot adequately describe the time-dependent dissociation phase. Indeed, we found that a fit using a heterogeneous ligand 2:1 binding model^45,50^ was statistically superior, with preferred chi-squared (χ^2^) and coefficient of determination (R-squared, *R* ^2^) values, to a homogeneous 1:1 model (experimental section; Supporting Information Tables S7 and S8 and Figure S7).^37,51^ Hence, we probed short- and long-lived GF-EGFR ECD binding interactions, as denoted by subscripts “1” and “2”, respectively. Interestingly, these kinetic determinations yielded equilibrium dissociation constants *K*_D‑1_ and *K*_D‑2_ within the same order of magnitude, in the hundreds of nanomolar range, for all high-affinity GFs. This means that the long-lived binding events of the GFs were substantially less frequent than their short-lived ones. In general, *K*_D‑1_ accounted for about 65% of all events. Indeed, the association rate constants of the short- and long-lived binding interactions, *k*_on‑1_ and *k*_on‑2_, were in the order of 10^5^ and 10^4^ M^–1^ s^–1^, respectively. For EGFR^CHO–K1^, *K*_D‑1_ (EGF) < *K*_D‑1_ (TGF-α) < *K*_D‑1_ (HB-EGF), while *K*_D‑2_ values are not statistically different among all examined GFs (Supporting Information Table S7).

**Figure 2:**
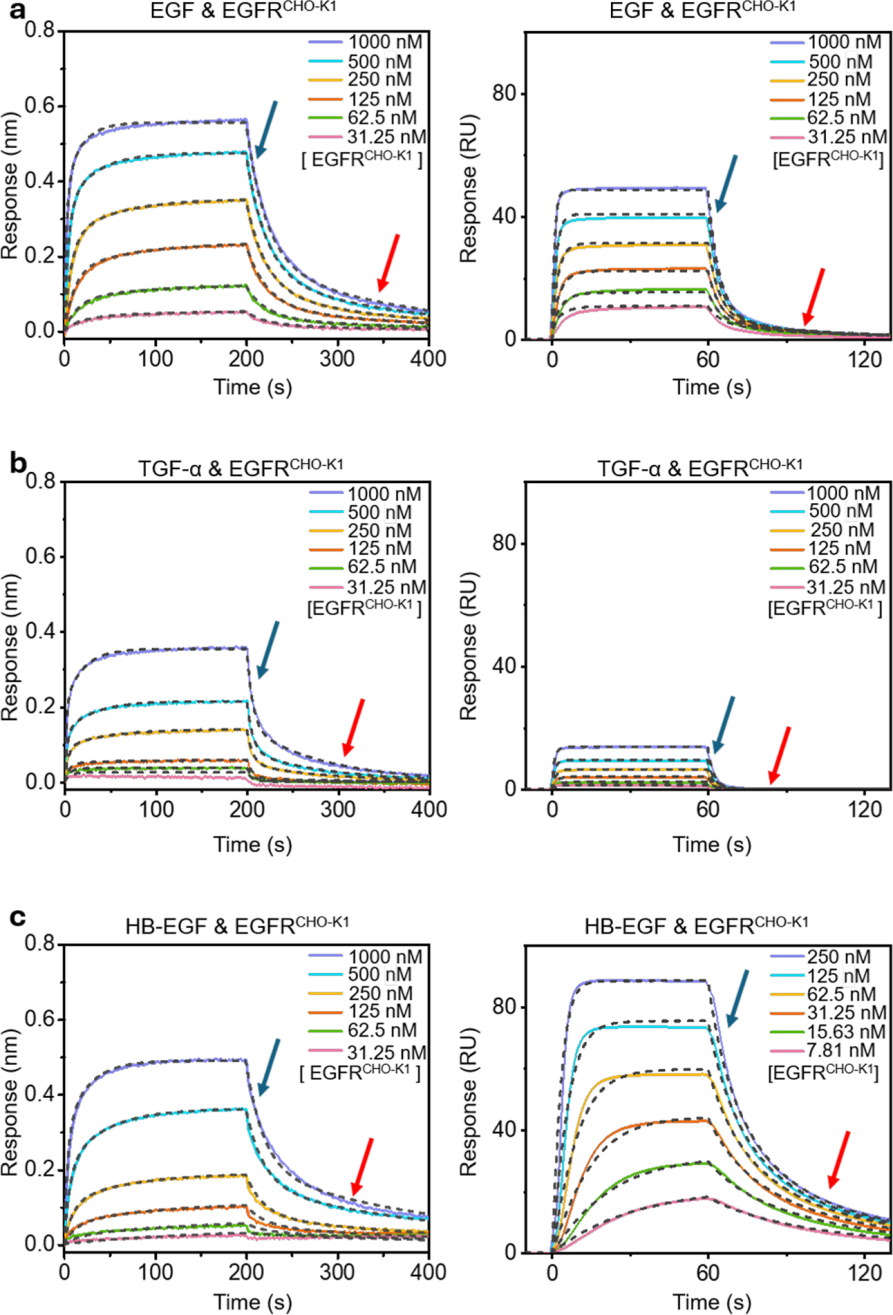
BLI and SPR analyses of GF-EGFR ECD interactions with the extracellular domain of EGFR expressed in CHO-K1 cells. The binding interactions between GFs and the extracellular domain of EGFR expressed in CHO-K1 cells (EGFR^CHO–K1^) were measured by biolayer interferometry (BLI) and surface plasmon resonance (SPR). (a) EGF-EGFR^CHO–K1^, (b) TGF-α-EGFR^CHO–K1^, and (c) HB-EGF-EGFR^CHO–K1^. Representative BLI (left) and SPR (right) sensorgrams show the association and dissociation phases. For BLI, 10 nM biotinylated GFs were immobilized on streptavidin-coated biosensors for 10 min, followed by the association phase with six 2-fold serial dilutions of EGFR^CHO–K1^ and the dissociation phase in GF-free buffer. For SPR, EGFR^CHO–K1^ was immobilized on Cytiva CM5 sensor chips, and GFs were injected into the solution. All binding curves were globally fitted (the black dotted lines) using a heterogeneous ligand 2:1 kinetic model. For BLI, the first 200 s represent the association phase, and 200–400 s represent the dissociation phase. For SPR, the first 60 s represent the association phase, and the 60–130 s represent the dissociation phase. Blue and red arrows indicate the fast- and slow-dissociation phases, respectively.

Similarly, we conducted real-time kinetics measurements of GF-EGFR^CHO‑K1^ interactions using SPR, which confirmed a two-substate binding profile, as indicated by a two-exponential response (Figure 2, right-hand panels; Tables S9 and S10 and Figure S8). We observed both quantitative and qualitative differences between the SPR and BLI measurements. The two binding substates showed higher event frequencies in SPR versus BLI. In fact, the association rate constants of the long- and short-lived binding interactions, *k*_on‑1_ and *k*_on‑2_, were on the order of 10^6^ and 10^5^ M^–1^ s^–1^, respectively (Table S9). Second, the short-lived binding events are less frequent than the long-lived interactions. However, the dissociation rate constants of the long-lived bindings by SPR are within the same order of magnitude as those values determined for the short-lived bindings by BLI. Further, they are the most frequently probed events across both approaches. The rise in the *k*_on_ values by nearly 10-fold in the SPR versus BLI measurements is expected, given the significantly higher translational diffusion coefficients of lower-molecular-weight GFs compared to EGFR^CHO–K1^ (Table S2 and Figure S2).^48^ Qualitatively, the covalent attachment of EGFR^CHO–K1^ onto the SPR sensor surface can also affect the *k*_off_ values in various ways. The EGF-binding site of EGFR ECD is located between domains D-I and D-III, which are considered relatively rigid.^52,53^ In contrast, there is greater flexibility in the conformations adopted by domains D-I and D-IV, which may facilitate rearrangements between domains D-I and D-III. Therefore, the relative positions of domains D-I and D-III of the EGFR ECD when attached to the SPR sensor surface may differ from the receptor’s average conformation in solution, affecting both the short- and long-lived binding substates.

Using BLI, we obtained *K*_D‑1_ and *K*_D‑2_ values of 153 ± 20 nM and 162 ± 20 nM, respectively, for EGF-EGFR^CHO‑K1^. In contrast, the *K*_D‑1_ and *K*_D‑2_ values were 27.8 ± 1.6 nM and 441 ± 18 nM, respectively, as determined by SPR measurements. Our SPR results agree with prior determinations under similar experimental conditions (Table S11),^47,54^ which also revealed a two-substate kinetic interaction model. As a result of these conceptual distinctions between the two immobilization-based approaches, we also observed that interactions between individual GFs are affected differently by SPR measurements than by BLI determinations. For example, *K*_D‑1_ (HB-EGF) < *K*_D‑1_ (EGF) < *K*_D‑1_ (TGF-α) and *K*_D‑2_ (HB-EGF) < *K*_D‑2_ (EGF) < *K*_D‑2_ (TGF-α) by SPR measurements, contrasting BLI examinations above. In addition, we tentatively interpret that SPR provides stronger binding affinities of GFs to EGFR^CHO–K1^, primarily because of the enhanced translational diffusion coefficient of the freely moving GFs.^48^ Moreover, the two approaches probe distinct interaction strengths of GFs with the EGFR ECD relative to each other, likely due to subtle differences in binding-site exposure and dynamic conformations when GFs are immobilized versus free in solution.^55^ Remarkably, our BLI measurements probing TGF-α-EGFR^CHO–K1^ interactions with TGF-α attached onto the sensor surface were consistent with an earlier proposed two-substate binding model for SPR measurements using the same immobilization reactant and a closely similar salt concentration (Tables S7 and S12).^56^ Also, previous computational^22^ and experimental^57^ studies indicated high-affinity interactions between HB-EGF and EGRF ECD,^12,13^ with a *K*_D_ in the low-nanomolar range (Table S13).

### N151-Glycan Chain Is Not Essential for High-Affinity GF-EGFR ECD Interactions

Next, we wanted to test the sensitivity of these approaches to local and global alterations in the distribution of N-glycosylation side chains in the EGFR ECD. For a local modification of the glycosyl side chains, Asn151 (N151) is a glycosylation site in the extracellular domain of EGFR that is directly involved in the ligand-binding cleft (Figure S9).^58^ We expected to observe changes in binding affinity upon elimination of the N-glycan chain at this position. For example, MD simulations previously revealed that glycan moieties at N151 stabilize the local EGFR conformation within the binding cleft, thereby increasing affinity.^25,27^

In the absence of the N-glycan side chain at this site, GFs would bind less strongly to the EGFR ECD. Motivated by this idea, we created a single-alanine mutation at N151 (EGFR^N151A^).

Surprisingly, BLI measurements revealed that all high-affinity GFs examined interacted slightly stronger with EGFR^N151A^ than with the wild-type protein (Figure 3; Table S14). The two-substate binding profile observed during dissociation was also seen with this mutant, as a standard 1:1 binding model failed to fit the experimental data with statistical significance (Figure S10 and Table S15). Additionally, a high-affinity interaction of GFs with EGFR^N151A^ and a heterogeneous 2:1 binding model were confirmed by SPR measurements, which yielded kinetic and affinity parameters closely similar to EGFR^CHO–K1^ (Tables S16 and S17 and Figure S11).

**Figure 3:**
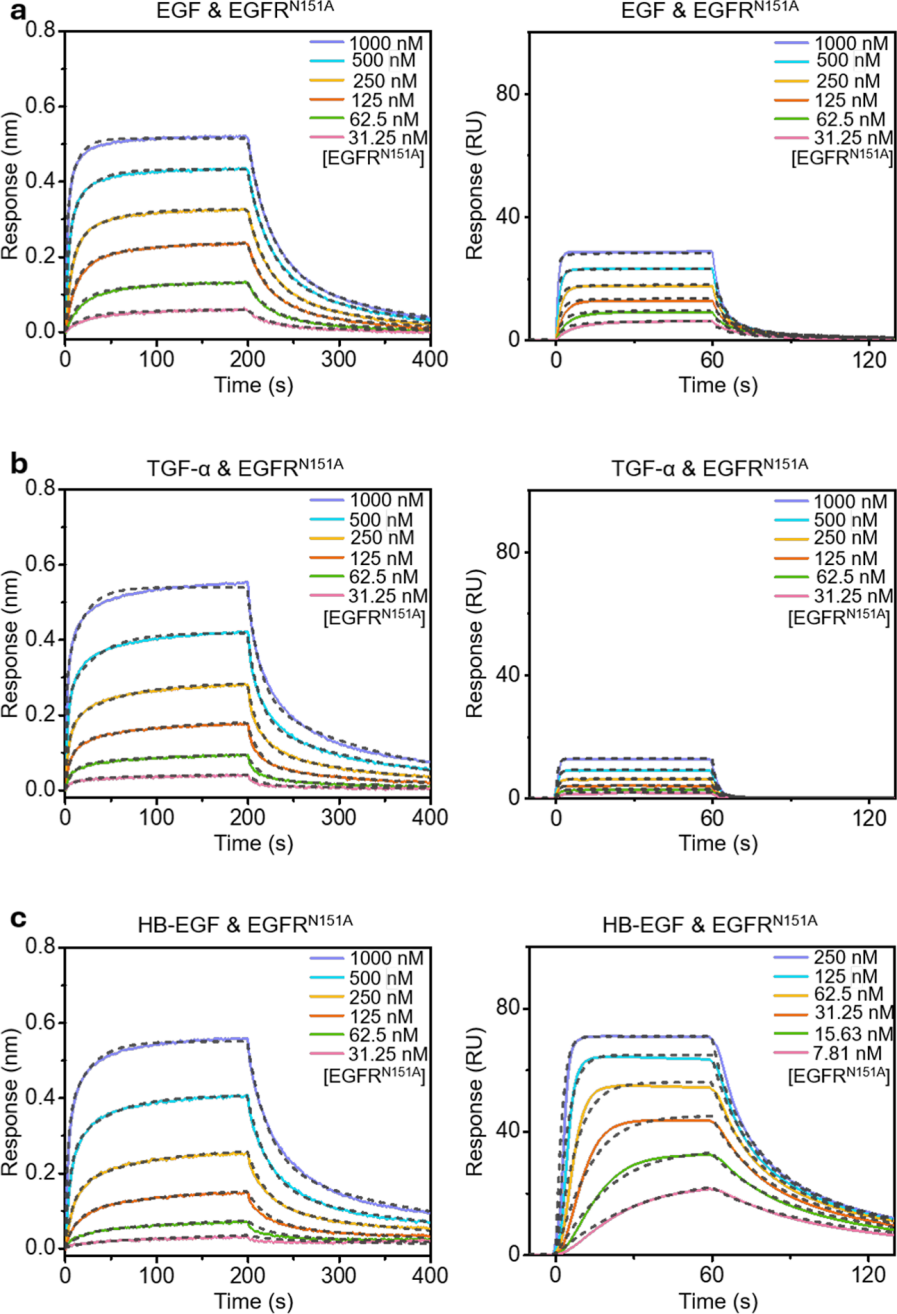
BLI and SPR analyses GF-EGFR interactions with the extracellular domain of EGFR^N151A^expressed in CHO-K1 cells. The binding between GFs and the extracellular domain of EGFR mutation expressed in CHO-K1 cells (EGFR^N151A^) was measured by biolayer interferometry (BLI) and surface plasmon resonance (SPR). (a) EGF-EGFR^N151A^, (b) TGF-α-EGFR^N151A^, and (c) HB-EGF-EGFR^N151A^. Representative BLI (left) and SPR (right) sensorgrams show the association and dissociation phases. For BLI, 10 nM biotinylated GFs were immobilized on streptavidin (SA) biosensors for 10 min, followed by association with six 2-fold serial dilutions of EGFR^N151A^ and dissociation in GF-free buffer. For SPR, EGFR^N151A^ was immobilized on Cytiva CM5 sensor chips, and GFs were injected into the solutions. All binding curves were globally fitted (the black dotted lines) using a heterogeneous ligand 2:1 kinetic model. For BLI, the first 200 s represent the association phase, and 200–400 s represent the dissociation phase. For SPR, the first 60 s represent the association phase, and the 60–130 s represent the dissociation phase.

### A PNGase F-Treated EGFR Isoform Shows Significantly Weaker Interactions with All High-Affinity GF Ligands

Next, we examined a PNGase F-treated EGFR isoform produced by CHO-K1 cells, which resulted in an extensive deglycosylation of the wild-type protein (^DG^EGFR^CHO‑K1^; experimental section and Figure S1). Our investigation was again prompted by previous computational studies demonstrating how N-glycosylation stabilizes the local structure of the EGFR ECD, which may influence membrane interactions^27^ and ligand-binding dynamics.^25,26^ Notably, all BLI measurements with immobilized GFs interacting with ^DG^EGFR^CHO‑K1^ added to the wells revealed markedly reduced interactions (Figure 4 and Table S18). For example, using BLI, we observed a 20-fold stronger interaction between EGF and the wild-type EGFR than with ^DG^EGFR^CHO‑K1^, as judged by *K*_D‑1_ values (Tables S7 and S18). Also, a 10-fold stronger interaction was observed with EGFR^CHO–K1^ than with ^DG^EGFR^CHO‑K1^, as judged by the *K*_D‑2_ values.

**Figure 4:**
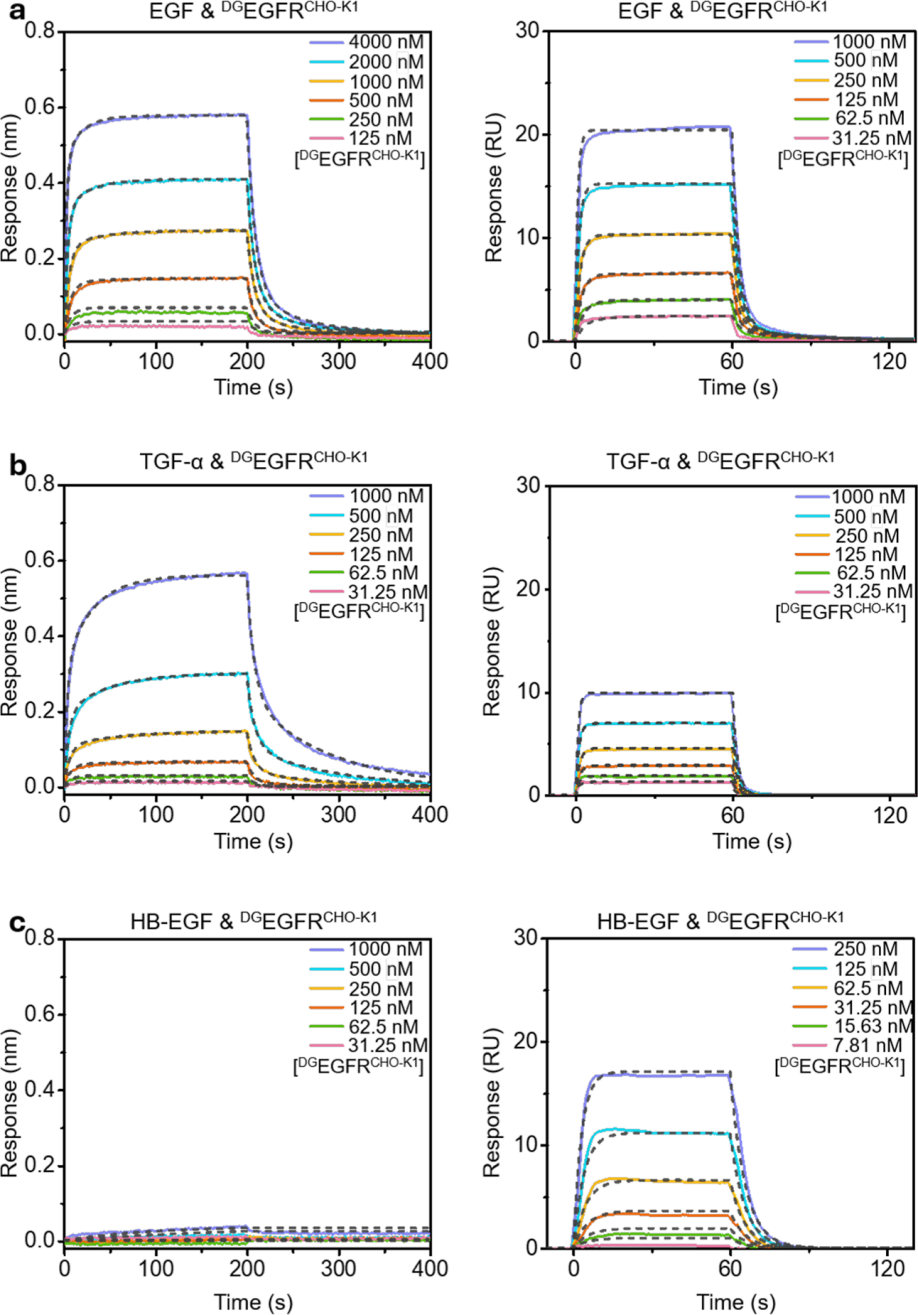
BLI and SPR analyses of interactions between high-affinity GFs and the PNGase F-treated EGFR. The EGFR ECD expressed in CHO-K1 cells (EGFR^CHO–K1^) was PNGase F-treated (^DG^EGFR^CHO‑K1^). Binding interactions between GFs and ^DG^EGFR^CHO‑K1^ were examined using BLI and SPR. (a) EGF-^DG^EGFR^CHO‑K1^, (b) TGF-α-^DG^EGFR^CHO‑K1^, and (c) HB-EGF-^DG^EGFR^CHO‑K1^. Representative BLI (left) and SPR (right) sensorgrams show the association and dissociation phases. For BLI, 10 nM biotinylated GFs were immobilized on streptavidin-coated biosensors for 10 min, followed by the association phase with six 2-fold serial dilutions of ^DG^EGFR^CHO‑K1^, and the dissociation phase in GF-free buffer. For SPR, ^DG^EGFR^CHO‑K1^ was immobilized on Cytiva CM5 sensor chips, and GFs were injected into the solution. Sensorgrams were globally fitted using a heterogeneous ligand 2:1 interaction model (black dotted lines). For BLI, the first 200 s represent the association phase, and 200–400 s represent the dissociation phase. For SPR, the first 60 s represent the association phase, and the 60–130 s represent the dissociation phase.

These differences, caused by extensive EGFR deglycosylation, were less pronounced for TGF-α, with 4- and 3-fold differences, respectively. Surprisingly, we were unable to detect a BLI binding response to HB-EGF. Therefore, we conclude that ^DG^EGFR^CHO‑K1^ does not interact with HB-EGF when HB-EGF is immobilized on a BLI sensor surface. In addition, we tentatively interpret that a PNGase F-driven deglycosylation reaction of EGFR ECD fully destabilizes the local conformations that pertain to the essential binding interface for each GF. Moreover, our biosensing approach is sufficiently sensitive to detect drastic alterations in the binding affinity of this EGFR isoform to individual GFs. Furthermore, for EGF and TGF-α, the data corresponding to GF-^DG^EGFR^CHO–K1^ interactions were also best represented by a heterogeneous 2:1 binding model (Figure S12 and Tables S18 and S19).

One immediate question is whether the extensive deglycosylation through PNGase F treatment preserves the functionality of ^DG^EGFR^CHO‑K1^. To explore this further, we conducted independent experiments using an Adnectin 1,^59^ an antibody mimetic small protein based on the 94-residue fibronectin type III (FN3) domain.^60^ EGF and Adnectin 1 bind to the EGFR domain D-I through overlapping binding surfaces.^36^ However, when interacting with EGFR, Adnectin 1 is not exposed to glycan side chains at Asn151. Therefore, it serves as an appropriate control protein binder to evaluate the functional folding state of the EGFR ECD under PNGase F treatment conditions. Biotinylated Adnectin 1 was attached to streptavidin-coated BLI sensors, and EGFR isoforms at various concentrations were added to the sensor wells (Figure S13). Notably, we observed that EGFR^CHO–K1^ and ^DG^EGFR^CHO‑K1^ exhibited identical high-affinity interactions at low nanomolar levels, consistent with previous findings (Table S20).^36,59^ It should be noted that in these BLI experiments, Adnectin 1-EGFR interactions were best fit by a homogeneous 1:1 binding model.

Next, we analyzed the effects of the PNGase treatment on the GF-EGFR ECD binding interactions using related SPR measurements (Figure 4). Our SPR-based kinetic analysis of the ^DG^EGFR^CHO‑K1^ isoform confirmed that all GFs have relatively weaker affinities compared to the wild-type protein (Figure 4, Tables S21 and S22 and Figure S14). In this case, the short-lived substate was markedly faster and not well resolved in the SPR measurements, so a second substate was not identified with sufficient statistical confidence using a heterogeneous 2:1 binding model. GF binds to the EGFR ECD between domains D-I and D-III (Figure 1a–c). While D-I and D-III are relatively rigid, D-II and D-IV can adopt numerous conformations that place D-III in different orientations relative to D-I.^52,53^ By immobilizing the EGFR ECD on the SPR sensor surface, such flexible orientations are likely eliminated. Hence, we speculate that steric constraints may shorten the GF binding time to the immobilized EGFR ECD. In addition, under these conditions, the short-lived substate reaches the boundary at which the SPR cannot resolve such events.

Interestingly, although HB-EGF-^DG^EGFR^CHO–K1^ interactions were absent in the BLI sensorgrams, we observed a response in our SPR experiments with a submicromolar binding affinity. This indicates a more flexible structure of ^DG^EGFR^CHO‑K1^ in solution, likely disrupting its binding interface with this GF, compared to when it is immobilized on the SPR sensor surface. In addition, through both the BLI and SPR measurements, we find that the interactions of HB-EGF with ^DG^EGFR^CHO‑K1^ are most affected among all high-affinity GFs examined in this work.

### Interactions of High-Affinity GFs with EGFR Isoforms Expressed by Human Expi293F Cells

In the final part of this study, we investigated the wild-type EGFR ECD protein expressed in human Expi293F cells (EGFR^Expi293F^) and found weaker interactions with all GFs using BLI (Figures S15 and S16 and Tables S23 and S24). We postulate that differences in GF affinity between CHO-K1- and Expi293F-derived EGFR isoforms are related to differences in their glycan profiles. These cell lines derive from hamsters and humans, respectively, and produce distinct glycan signatures. The quantity, location, and complexity of the glycan chains covalently attached to the N-sites after protein translation are expected to differ slightly between these isoforms. These differences would account for changes in ligand-binding kinetics because alterations in the glycan moieties of the EGFR ECD affect its stability, both locally and potentially globally.^25–27^ These findings were confirmed by SPR measurements (Figure S15 and S17 and Tables S25 and S26) with EGF and TGF-α. However, a striking deviation from this trend was observed in SPR experiments with HB-EGF, where the two-substate kinetic and equilibrium constants were identical for the CHO-K1 and Expi293F cell lines (Tables S9 and S25).

We extended these measurements to an Expi293F cell line that expresses only an EGFR ECD isoform lacking complex, bulky glycan side chains (EGFR^GnTI^; Figure S2 and Table S6).

We discovered that this EGFR isoform exhibits a further decline in binding interactions. Given its less complex high-mannose N-glycan chains (Figure S3 and Table S6), this finding is in accordance with substantially weakened GF-^DG^EGFR^CHO‑K1^ interactions discussed above. However, HB-EGF-EGFR^GnTI^ interactions again remained unchanged in SPR experiments as compared with HB-EGF-EGFR^Expi293F^ (Figures S18–S20 and Tables S27–S30). Independently conducted SPR binding experiments of HB-EGF with immobilized EGFR^CHO–K1^, EGFR^Expi293F^, and EGFR^GnTI^ isoforms consistently suggest that these high-affinity interactions do not depend on the EGFR isoform. This likely occurs because HB-EGF is the largest high-affinity GF tested, and EGFR is immobilized on the SPR surface, making it more challenging to probe the deep binding cleft (Figure 1a–c). We conclude that complexity and diverse distributions of N-glycans of these EGFR ECD isoforms have distinctive implications in the kinetics and dynamics of their interactions with individual GF ligands (Figures 5, S21 and S25). Finally, we present compelling evidence that distinct sensing readouts and immobilization strategies of one reactant versus the other can illuminate distinct aspects of the interactions.

**Figure 5:**
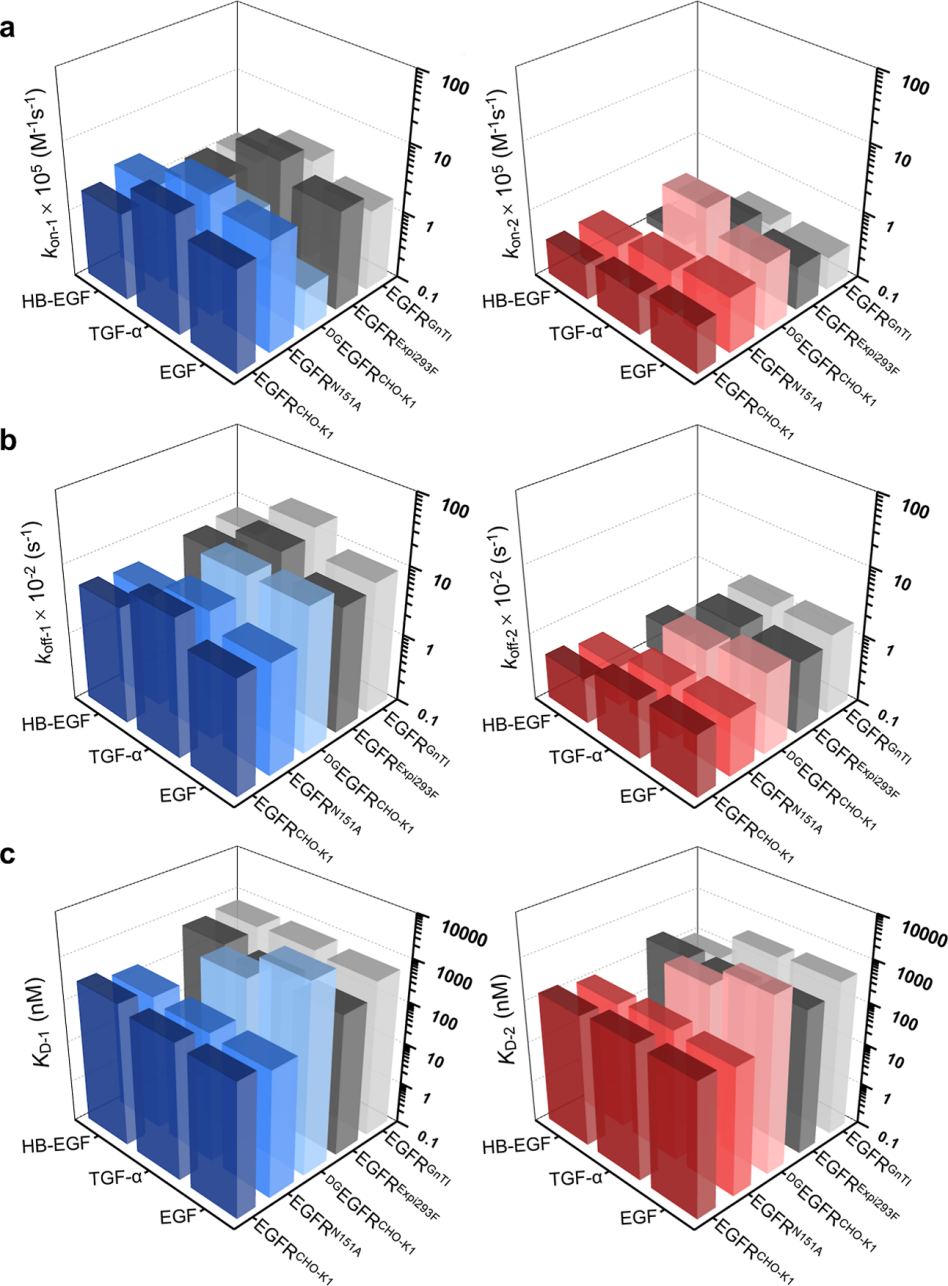
Logarithmic 3D plots summarizing the kinetic and affinity constants of the GF–EGFR ECD interactions using BLI. (a) The association rate constants of the fast and slow substates. (b) The dissociation rate constants of the fast and slow substates. (c) The equilibrium dissociation constants of the fast and slow substates.

## Concluding Remarks

Using two independent biosensing readouts, we demonstrate that high-affinity GF-EGFR ECD interactions exhibit both fast and slow binding substates, regardless of the EGFR ECD isoform. In addition, we provide compelling evidence that the design, composition, and readout of the immobilization-based sensing approach can influence not only the magnitude of kinetic and affinity parameters but also the relative interactions among different ligands and receptor isoforms. These distinctions clearly indicate that each biosensing approach probes the same bimodal protein recognition of the EGFR ECD in different physical ways, reflecting the constraints imposed on the immobilized molecule. We also show that a PNGase F-treated EGFR ECD isoform, which undergoes extensive deglycosylation at the N sites, binds to all tested GF ligands with significantly lower affinity than the wild-type protein. In accord with prior computational studies,^25–27^ we suggest that N-glycans affect these interactions by altering the local structural stability of the EGFR ECD. Our comparative approach could be applied to other ligand–receptor systems to directly examine how specific posttranslational modifications influence their interactions.

## Supplementary Material



## Notes

Amino acid sequences and other properties of proteins used in this study, Glycan mapping of the EGFR isoforms, Optimization of the linker length for acquiring the GF-EGFR interactions via BLI recordings, Kinetic and affinity constants of high-affinity GFs with wild-type EGFR expressed in the CHO-K1 cell line (EGFR^CHO–K1^) using BLI, Kinetic and affinity constants of high-affinity GFs with EGFR^CHO–K1^ using SPR, Kinetic and affinity constants of GF-EGFR interactions reported by other research groups under different experimental circumstances and using diverse methods, Structural mapping of N-glycosylation sites of EGFR ECD and the specific location of N151 within the EGF binding site, Kinetic and affinity constants of high-affinity GFs with EGFR^N151A^ expressed in the CHO-K1 cell line using BLI, Kinetic and affinity constants of high-affinity GFs with EGFR^N151A^ expressed in the CHO-K1 cell line using SPR, Kinetic and affinity constants of high-affinity GFs with the PNGase F-treated EGFR isoform (^DG^EGFR^CHO‑K1^) expressed in the CHO-K1 cell line using BLI, The negative-control experiment for demonstrating the functional state of ^DG^EGFR^CHO‑K1^ using BLI, Kinetic and affinity constants of high-affinity GFs with ^DG^EGFR^CHO‑K1^ using SPR, Kinetic and affinity constants of high-affinity GFs with EGFR^Expi293F^ using BLI and SPR, Kinetic and affinity constants of high-affinity GFs with EGFR^GnTI^ using BLI and SPR, Two-dimensional plots of the kinetic and affinity constants of GF-EGFR interactions for various EGFR isoforms using BLI, Two-dimensional plots of the kinetic and affinity constants of GF-EGFR interactions for various high-affinity GFs against specific EGFR isoforms using BLI, Supplementary references (PDF)
