# Supplementary material for: Comparative Real-Time Kinetics of Ligand–Receptor Interactions Using Immobilization-Based Sensing Readouts
Source: Anal Chem. 2026 Jul 9;98(28):21022–33. doi: 10.1021/acs.analchem.6c02900 (PMC13393086; doi:10.1021/acs.analchem.6c02900)
Supplement: Supplementary file 1 [file ac6c02900_si_001.pdf]

SUPPORTING INFORMATION FILE

Comparative Real-Time Kinetics of Ligand-Receptor Interactions Using Immobilization-Based Sensing Readouts

Yazheng Wang[1,2], Yalun Wu[3,4], Lauren A. Mayse[1,5], Danny Capucilli[3], Aaron J. Wolfe[1,3,4], and Liviu Movileanu[1,2,5,6*]

[1]Department of Physics, Syracuse University, 201 Physics Building, Syracuse, New York 13244, United States

[2]Department of Biomedical and Chemical Engineering, Syracuse University, 329 Link Hall, Syracuse, New York 13244, United States

[3]Ichor Life Sciences, Inc., 831 James Street, Syracuse, New York 13203, United States

[4]Department of Chemistry, State University of New York, College of Environmental Science and Forestry, 1 Forestry Dr., Syracuse, New York 13210, United States

[5]The BioInspired Institute, Syracuse University, Syracuse, New York, 13244, United States

[6]Department of Biology, Syracuse University, 114 Life Sciences Complex, Syracuse, New York 13244, United States

*The corresponding author:
Liviu Movileanu, PhD, Phone: 315-443-8078; E-mail: lmovilea@syr.edu

TABLE OF CONTENTS Page

1-Amino acid sequences and other properties of proteins used in this study S3

2-Glycan mapping of the EGFR isoforms S7

3-Optimization of the linker length for acquiring the GF-EGFR interactions via BLI recordings S9

4-Kinetic and affinity constants of high-affinity GFs with wild-type EGFR expressed in the CHO-K1 cell line (EGFR^{CHO-K1}) using BLI S11

5-Kinetic and affinity constants of high-affinity GFs with EGFR^{CHO-K1} using SPR S14

6-Kinetic and affinity constants of GF-EGFR interactions reported by other research groups under different experimental circumstances and using diverse methods S17

7-Structural mapping of N-glycosylation sites of EGFR ECD and the specific location of N151 within the EGF binding site S18

8-Kinetic and affinity constants of high-affinity GFs with EGFR^{N151A} expressed in the CHO-K1 cell line using BLI S19

9-Kinetic and affinity constants of high-affinity GFs with EGFR^{N151A} expressed in the CHO-K1 cell line using SPR S22

10-Kinetic and affinity constants of high-affinity GFs with the PNGase F-treated EGFR isoform (^{DG}EGFR^{CHO-K1}) expressed in the CHO-K1 cell line using BLI S25

11-The negative-control experiment for demonstrating the functional state of ^{DG}EGFR^{CHO-K1} using BLI S28

12-Kinetic and affinity constants of high-affinity GFs with ^{DG}EGFR^{CHO-K1} using SPR S30

13-Kinetic and affinity constants of high-affinity GFs with EGFR^{Expi293F}

using BLI and SPR S33

14-Kinetic and affinity constants of high-affinity GFs with $EGFR^{GnTI}$

using BLI and SPR S40

15-Two-dimensional plots of the kinetic and affinity constants of GF-EGFR interactions for

various EGFR isoforms using BLI S47

16-Two-dimensional plots of the kinetic and affinity constants of GF-EGFR interactions for

various high-affinity GFs against specific EGFR isoforms using BLI S49

17-Supplementary references S52

1. Amino acid sequences and other biochemical properties of proteins used in this study.

Supplementary Table S1. Sequences of growth factor proteins used in this work.

**Protein sequence for EGF**
NSDSECPLSH DGYCLHDGVC MYIEALDKYA CNCVVGYIGE RCQYRDLKWW ELR
**Protein sequence for TGF-α**
VVSHFNDCPD SHTQFCFHGT CRFLVQEDKP ACVCHSGYVG ARCEHADLLA
**Protein sequence for HB-EGF**
DLQEADLDLL RVTLSSKPQA LATPNKEEHG KRKKKGKGLG KKRDPCLRKY KDFCIHGECK YVKELRAPSC ICHPGYHGER CHGLSL

Supplementary Table S2. Protein Data Bank (PDB) codes of GF ligand structures used in this work. Here, EGFR is the ectodomain (ECD) of the human epidermal growth factor receptor.

Growth factor or growth factor-receptor complex	Molecular weight	PDB code and reference
EGF	6.2 kDa	1JL9[1]
TGF-α	5.5 kDa	2TGF[2]
HB-EGF	9.7 kDa	1XDT[3]
EGF–EGFR	75.7 kDa	8HGS[4]
TGF-α–EGFR	75 kDa	7SZ7[5]
HB-EGF–EGFR	79.2 kDa	NA*

*Crystal structure of the HB-EGF–EGFR complex is not available. In **Figure 1**, the structure of this bimolecular complex was predicted using AlphaFold 3.[6]

Supplementary Table S3. The amino acid sequence of the ectodomain of the human epidermal growth factor receptor (EGFR) proteins (wild-type and mutants). In this study, we use the amino acid sequence 25-645 of EGFR ECD. This sequence also includes a hexahistidine tag.

**Protein sequence for EGFR**
VLEEKKVCQGTSNKLTQLGTFEDHFLSLQRMFNNCEVVLGNLEITYVQRNYDLSFLK<br>TIQEVAGYVLIALNTVERIPLENLQIIRGNMYYENSYALAVLSNYDANKTGLKELPMR<br>NLQEILHGAVRFSNNPALCNVESIQWRDIVSSDFLSNMSMDFQNHLGSCQKCDPSCPN<br>GSCWGAGEENCQKLTKIICAQQCSGRCRGKSPSDCCHNQCAAGCTGPRESDCLVCRK<br>FRDEATCKDTCPPLMLYNPTTYQMDVNPEGKYSFGATCVKKCPRNYVVTDHGSCVR<br>ACGADSYEMEEDGVRKCKKCEGPCRKVCNGIGIGEFKDSLSINATNIKHFKNCTSISG<br>DLHILPVAFRGDSFTHTPPLDPQELDILKTVKEITGFLLIQAWPENRTDLHAFENLEIIR<br>GRTKQHGQFSLAVVSLNITSLGLRSLKEISDGDVIISGNKNLCYANTINWKKLFGTSGQ<br>KTKIISNRGENSCKATGQVCHALCSPEGCWGPEPRDCVSCRNVSRGRECVDKCNLLE<br>GEPREFVENSECIQCHPECLPQAMNITCTGRGPDNCIQCAHYIDGPHCVKTCPAGVMG<br>ENNTLVWKYADAGHVCHLCHPNCTYGCTGPGLEGCPTNGPKIPSHHHHHH
**Protein sequence for EGFR^{N151A}**
VLEEKKVCQGTSNKLTQLGTFEDHFLSLQRMFNNCEVVLGNLEITYVQRNYDLSFLK<br>TIQEVAGYVLIALNTVERIPLENLQIIRGNMYYENSYALAVLSNYDANKTGLKELPMR<br>NLQEILHGAVRFSNNPALCNVESIQWRDIVSSDFLSAMSMDFQNHLGSCQKCDPSCPN<br>GSCWGAGEENCQKLTKIICAQQCSGRCRGKSPSDCCHNQCAAGCTGPRESDCLVCRK<br>FRDEATCKDTCPPLMLYNPTTYQMDVNPEGKYSFGATCVKKCPRNYVVTDHGSCVR<br>ACGADSYEMEEDGVRKCKKCEGPCRKVCNGIGIGEFKDSLSINATNIKHFKNCTSISG<br>DLHILPVAFRGDSFTHTPPLDPQELDILKTVKEITGFLLIQAWPENRTDLHAFENLEIIR<br>GRTKQHGQFSLAVVSLNITSLGLRSLKEISDGDVIISGNKNLCYANTINWKKLFGTSGQ<br>KTKIISNRGENSCKATGQVCHALCSPEGCWGPEPRDCVSCRNVSRGRECVDKCNLLE<br>GEPREFVENSECIQCHPECLPQAMNITCTGRGPDNCIQCAHYIDGPHCVKTCPAGVMG<br>ENNTLVWKYADAGHVCHLCHPNCTYGCTGPGLEGCPTNGPKIPSHHHHHH

Supplementary Table S4. List of primers used for EGFR^{N151A}.

Primer name	Sequences (5´-3´)
EGFR^{N151A} _forward	CTTTCTCAGCGCCATGTCGATGGAC
EGFR^{N151A} _reverse	TCACTGCTGACTATGTCCCGCCA

Supplementary Table S5. The amino acid sequence of the PNGaseF enzyme used in this work. This sequence also includes a hexahistidine tag.

**Protein sequence for PNGaseF**
MKKTAIAIAVALAGFATVAQAGIPAPADNTVNIKTFDKVKNAFGDGLSQSAEGTFTFP<br>ADVTAVKTIKMFIKNECPNKTCDEWDRYANVYVKNKTTGEWYEIGRFITPYWVGTE<br>KLPRGLEIDVTDFKSLLSGNTELKIYTETWLAKGREYSVDFDIVYGTPDYKYSAVVPV<br>VQYNKSSIDGVPYGKAHTLALKKNIQLPTNTEKAYLRTTISGWGHAKPYDAGSRGCA<br>EWCFRTHTIAINNSNTFQHQLGALGCSANPINNQSPGNWTPDRAGWCPGMAVPTRID<br>VLNNSLIGSTFSYEYKFQNWTNNGTNGDAFYAISSFVIAKSNTPISAPVVTNGENLYFQ<br>GSDPHHHHHH

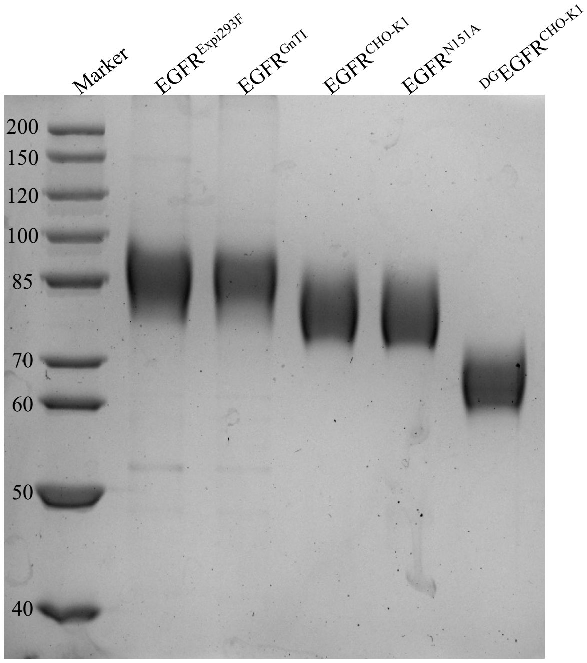

Supplementary Figure S1. An SDS-PAGE gel analysis of the EGFRs. The purity and size of all EGFR ECD isoforms were checked by an 8% SDS-PAGE gel analysis. The expected molecular weight of PNGase F-treated $EGFR^{CHO\text{-}K1}$ ($^{DG}EGFR^{CHO\text{-}K1}$) is 69.6 kDa (**Supplementary Figure S2**). However, we observed a higher apparent MW due to N-glycosylation at Asn sites.

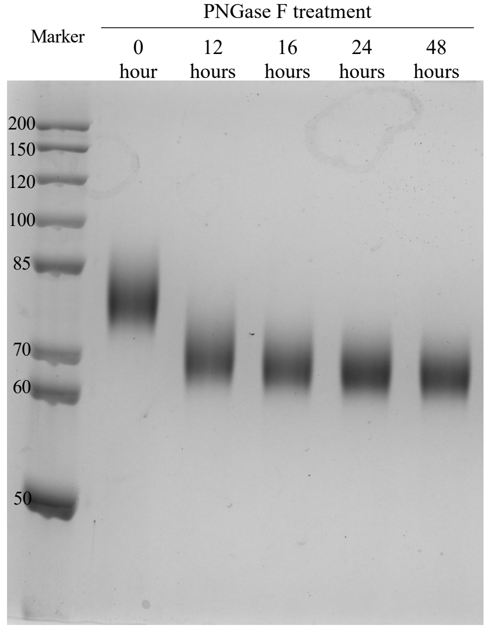

<u>Supplementary Figure S2.</u> An SDS-PAGE gel image showing PNGase F-treated EGFR ECD expressed in CHO-K1 cells. EGFR was incubated with PNGase F at 37°C, and protein samples were analyzed at different time points. The molecular weight decreased over time and reached a plateau after 24 hours, with no further reduction.

2. Glycan mapping at Asn sites of the EGFR ECD isoforms.

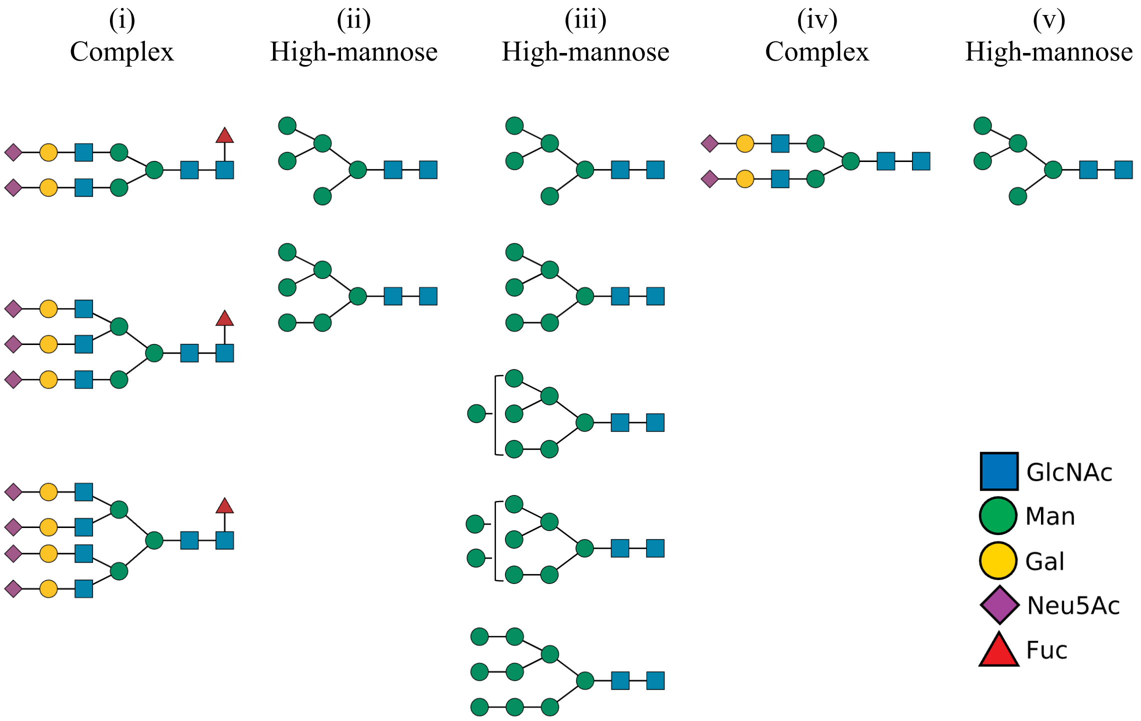

Supplementary Figure S3. Compositions of N-glycans of EGFR. The illustration shows the compositions of N-glycans identified for EGFR. Glycan symbols follow the Symbol Nomenclature for Glycans (SNFG) conventions. The blue square represents N-acetylglucosamine (GlcNAc). The green circle indicates mannose (Man). The yellow circle denotes galactose (Gal). The purple diamond represents sialic acid (Neu5Ac). The red triangle shows fucose (Fuc).[7] N-glycan structure data were obtained from GlycoShape.[8]

<u>Supplementary Table S6.</u> Summary of N-glycans of EGFR produced in CHO-K1 and Expi293F GnTI cells. The left column indicates glycosylation occupancy, and the right column shows the type of N-glycan side chains. In the glycosylation occupancy columns, "◯" signifies 100% glycosylation, "×" indicates no glycosylation, and "–" denotes that no supporting data are available. N.A. stands for not applicable. Mass spectrometry data for the CHO-K1 cell line EGFR ($EGFR^{CHO\text{-}K1}$) glycans were obtained from Hasegawa et al. (2015).[9] The Expi293F GnTI cell line is a modified expression system lacking N-acetylglucosaminyltransferase I (GnTI), an enzyme required for the maturation of complex N-linked glycans. Consequently, proteins expressed in these cells predominantly carry high-mannose N-glycans, mainly $Man_5GlcNAc_2$, as shown in group (v).[10]

Cell type		CHO-K1						Expi293F GnTI	
Protein		$EGFR^{CHO\text{-}K1}$		$EGFR^{N151A}$		$^{DG}EGFR^{CHO\text{-}K1}$		$EGFR^{GnTI}$	
Domain	site	Occupancy	Glycan type	Occupancy	Glycan type	Occupancy	Glycan type	Occupancy	Glycan type
I	N32	◯	(i)	◯	(i)	×	N.A.	–	(v)
	N104	Partial	(i)	Partial	(i)	×	N.A.	–	(v)
	N151	◯	(i)	×	N.A.	×	N.A.	–	(v)
II	N172	Partial	(i)	Partial	(i)	×	N.A.	–	(v)
III	N328	◯	(ii)	◯	(ii)	×	N.A.	–	(v)
	N337	◯	(iii)	◯	(iii)	×	N.A.	–	(v)
	N389	◯	(i)	◯	(i)	×	N.A.	–	(v)
	N420	◯	(iv)	◯	(iv)	×	N.A.	–	(v)
IV	N504	◯	(i)	◯	(i)	×	N.A.	–	(v)
	N544	◯	(i)	◯	(i)	×	N.A.	–	(v)
	N579	◯	(i)	◯	(i)	×	N.A.	–	(v)
	N599	Partial	(i)	Partial	(i)	×	N.A.	–	(v)

3. Optimization of the linker length for acquiring the GF-EGFR interactions via biolayer interferometry (BLI) recordings.

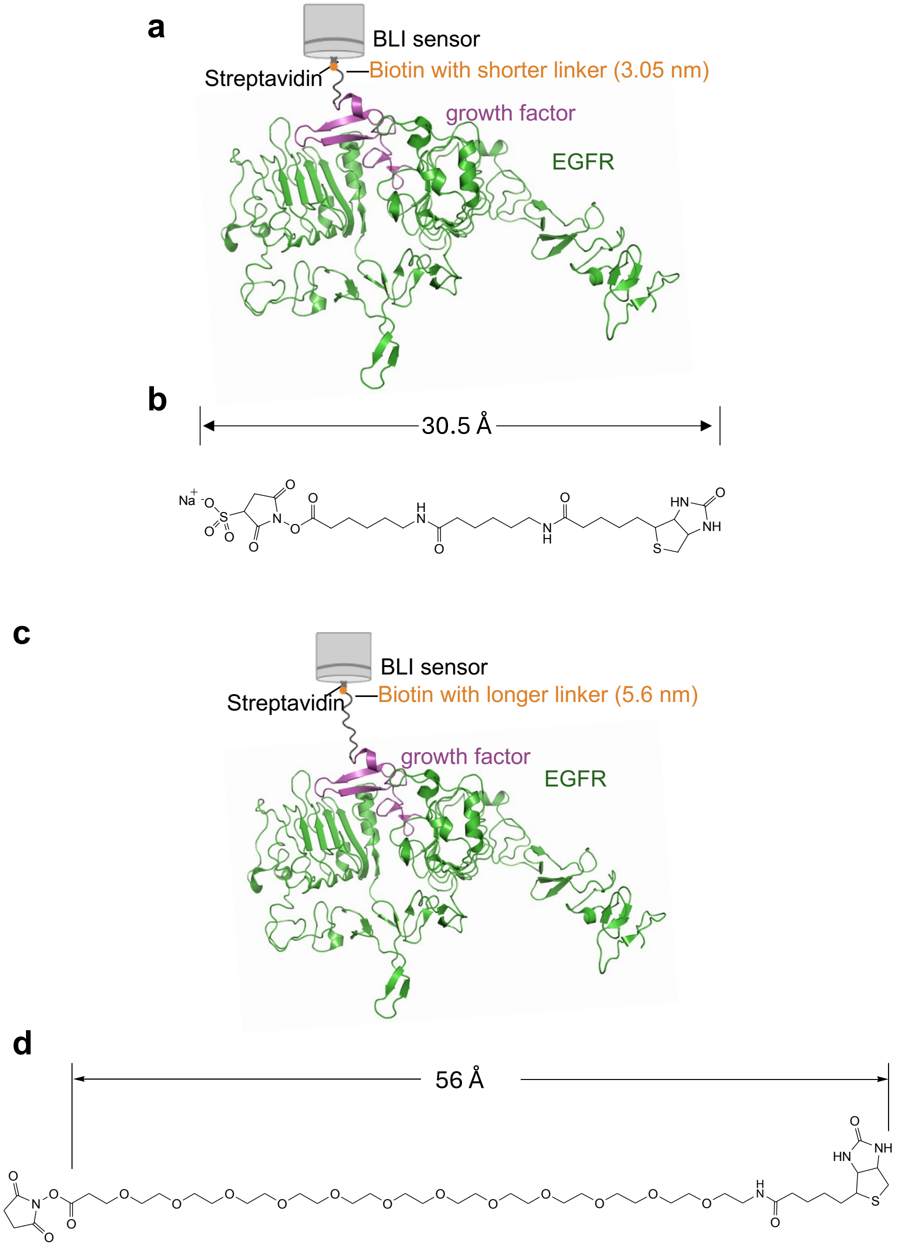

__Supplementary Figure S4.__ **Optimization of the biotinylated linker length for probing binding interactions between the high-affinity GF ligand immobilized on the sensor surface and EGFR ECD in the sensor wells. (a)** A short biotinylated linker, EZ-Link Sulfo-NHS-LC-LC-biotin, was chemically attached to EGF using NHS-ester–mediated amine coupling chemistry (**Experimental section**).[11] **(b)** Chemical structure of EZ-Link Sulfo-NHS-LC-LC-biotin, with an overall length of 3.05 nm. **(c)** A long biotinylated linker, EZ-Link NHS-$(PEG)_{12}$-biotin, was chemically attached to EGF using NHS-ester–mediated amine coupling chemistry (**Experimental section**).[11] **(d)** Chemical structure of EZ-Link NHS-$(PEG)_{12}$-biotin, with an overall length of 5.6 nm.

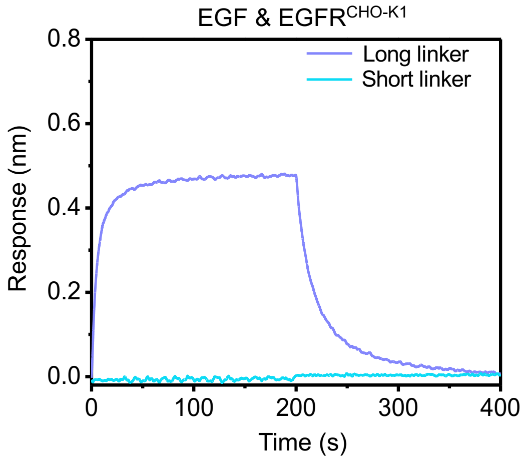

<u>Supplementary Figure S5.</u> BLI sensorgrams using long and short linkers. 50 nM biotinylated linkers were each chemically attached to EGF and loaded onto streptavidin-coated BLI sensors for 5 min. For the association phase, they were dipped into a running buffer containing 1000 nM EGFR$^{CHO\text{-}K1}$. For the dissociation phase, they were incubated in EGFR-free running buffer. Curves corresponding to long and short linkers are colored magenta and blue, respectively.

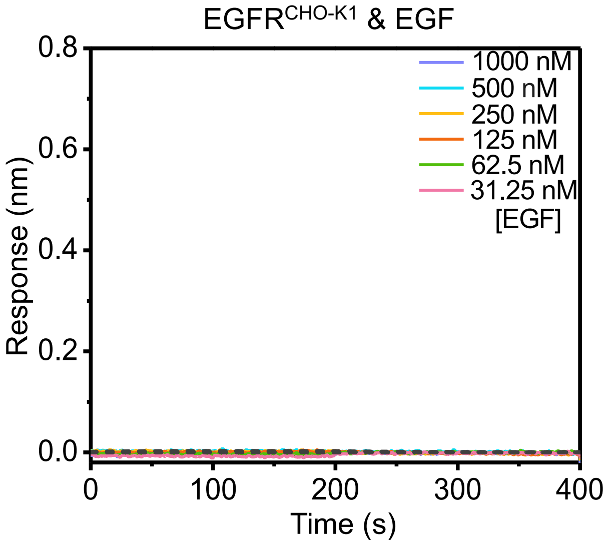

<u>Supplementary Figure S6.</u> Negative-control BLI experiment for an immobilization configuration with the EGFR ECD attached to the BLI surface. EGFR$^{CHO\text{-}K1}$ was immobilized on the BLI biosensor surface, and EGF was present in the wells. When EGFR ECD was immobilized, no detectable binding signal was observed, indicating that this BLI experimental design is not suitable for analyzing GF-EGFR ECD interactions.

4. Kinetic and affinity constants of high-affinity growth factors with the wild-type EGFR expressed in the CHO-K1 cell line using BLI.

<u>**Supplementary Table S7.**</u> **BLI-determined kinetic rate constants of association and dissociation, k_{on} and k_{off}, respectively, and equilibrium dissociation constants, K_D, of the GF-receptor interactions with the EGF, TGFα, or HB-EGF attached to the BLI chip surface, and $EGFR^{CHO\text{-}K1}$ added to the wells.** The running buffer consisted of 20 mM Tris-HCl, 150 mM KCl, and 1 mg/ml bovine serum albumin (BSA), 0.005% (v/v) Tween 20, pH 7.5. Values of all constants, which were derived using a heterogeneous ligand 2:1 binding model and global fits **(Experimental Section)**, indicate mean ± s.e.m. The average values are reported as mean ± s.d. and were obtained from n = 3 independent BLI experiments.

The quality of the fits was assessed using the reduced chi-squared (χ^2) and the coefficient of determination (R-squared, R^2). χ^2 quantifies the deviation between the experimental and fitted sensorgrams, while R^2 indicates how well the model predicts the binding response. Lower χ^2 and higher R^2 values indicate good agreement between the binding model and the acquired BLI data.

EGF & $EGFR^{CHO\text{-}K1}$								
**Repeat**	$k_{on\text{-}1}$ (×10^5 M^{-1}s^{-1})	$k_{off\text{-}1}$ (×10^{-2} s^{-1})	$K_{D\text{-}1}$ (nM)	$k_{on\text{-}2}$ (×10^4 M^{-1}s^{-1})	$k_{off\text{-}2}$ (×10^{-2} s^{-1})	$K_{D\text{-}2}$ (nM)	χ^2	R^2
**1**	2.76 ± 0.03	5.00 ± 0.03	181 ± 1	4.54 ± 0.06	0.873 ± 0.009	192 ± 3	0.039	0.999
**2**	3.12 ± 0.05	4.36 ± 0.04	140 ± 1	5.38 ± 0.11	0.934 ± 0.015	174 ± 5	0.072	0.999
**3**	3.27 ± 0.03	4.56 ± 0.03	139 ± 1	5.10 ± 0.06	0.615 ± 0.006	121 ± 2	0.025	0.999
**Average**	3.05 ± 0.21	4.64 ± 0.27	153 ± 20	5.01 ± 0.35	0.807 ± 0.138	162 ± 30		

TGF-α & $EGFR^{CHO\text{-}K1}$								
**Repeat**	$k_{on\text{-}1}$ (×10^5 M^{-1}s^{-1})	$k_{off\text{-}1}$ (×10^{-2} s^{-1})	$K_{D\text{-}1}$ (nM)	$k_{on\text{-}2}$ (×10^4 M^{-1}s^{-1})	$k_{off\text{-}2}$ (×10^{-2} s^{-1})	$K_{D\text{-}2}$ (nM)	χ^2	R^2
**1**	5.35 ± 0.13	9.53 ± 0.10	178 ± 1	4.14 ± 0.05	0.767 ± 0.006	185 ± 3	0.078	0.999
**2**	5.43 ± 0.12	10.0 ± 0.1	185 ± 1	4.15 ± 0.05	0.712 ± 0.005	172 ± 2	0.077	0.999
**3**	5.49 ± 0.13	10.4 ± 0.1	190 ± 1	4.01 ± 0.05	0.699 ± 0.006	174 ± 3	0.069	0.999
**Average**	5.43 ± 0.06	9.99 ± 0.36	184 ± 5	4.10 ± 0.06	0.726 ± 0.029	177 ± 6		

HB-EGF & $EGFR^{CHO\text{-}K1}$								
**Repeat**	$k_{on\text{-}1}$ (×10^5 M^{-1}s^{-1})	$k_{off\text{-}1}$ (×10^{-2} s^{-1})	$K_{D\text{-}1}$ (nM)	$k_{on\text{-}2}$ (×10^4 M^{-1}s^{-1})	$k_{off\text{-}2}$ (×10^{-2} s^{-1})	$K_{D\text{-}2}$ (nM)	χ^2	R^2
**1**	1.76 ± 0.02	5.68 ± 0.04	324 ± 1	2.94 ± 0.03	0.753 ± 0.004	256 ± 3	0.052	0.999
**2**	1.82 ± 0.03	3.87 ± 0.04	213 ± 1	3.53 ± 0.07	0.177 ± 0.007	50.1 ± 2.3	0.043	0.998
**3**	2.09 ± 0.05	5.23 ± 0.07	250 ± 1	3.69 ± 0.06	0.467 ± 0.008	126 ± 3	0.082	0.998
**Average**	1.89 ± 0.15	4.93 ± 0.77	262 ± 46	3.39 ± 0.32	0.465 ± 0.235	144 ± 85		

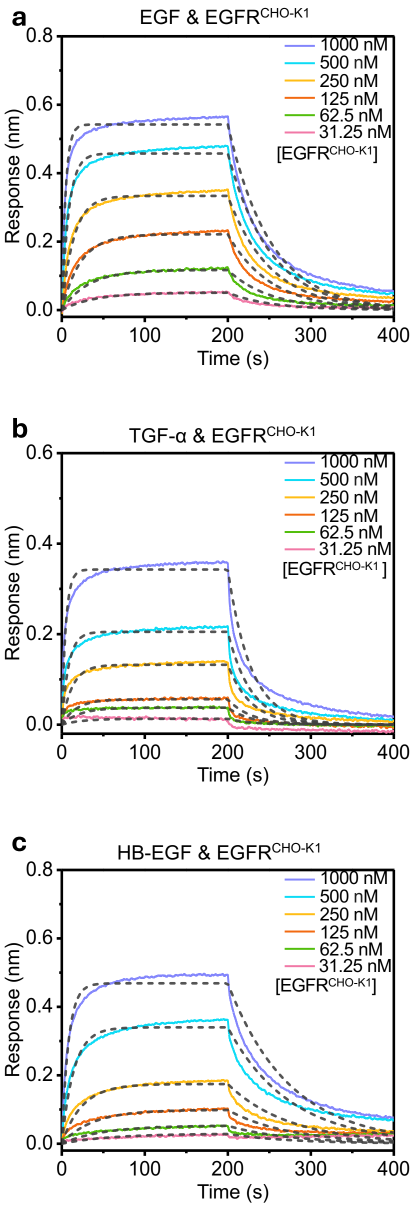

<u>Supplementary Figure S7.</u> BLI assays measuring the interactions between high-affinity GFs and the EGFR extracellular domain expressed by the CHO-K1 cell line. (a) EGF–EGFR^{CHO-K1}. **(b)** TGF-α–EGFR^{CHO-K1}. **(c)** HB-EGF–EGFR^{CHO-K1}. Representative BLI sensorgrams show the association and dissociation phases. For each panel, 10 nM biotinylated GF was loaded onto streptavidin-coated sensors for 10 min, then dipped into buffers containing six twofold serial dilutions of EGFR^{CHO-K1} from 1000 nM to 31.25 nM for the association phase. Sensors were subsequently transferred to EGFR^{CHO-K1}-free buffer for the dissociation phase. Dashed lines indicate fits of BLI sensorgrams showing interactions between EGFR^{CHO-K1} and its high-affinity GFs. The binding curves were globally fitted (the black dotted lines) using the FortéBio Octet Data Analysis software (FortéBio) with a homogeneous 1:1 binding model. The association phase is represented by the first 200 seconds, and the dissociation is indicated by the second 200 seconds.

<u>Supplementary Table S8.</u> BLI-determined kinetic rate constants of association and dissociation, k_{on} and k_{off}, respectively, and equilibrium dissociation constants, K_D, of the GF-receptor interactions with EGF, TGF-α, or HB-EGF attached to the BLI chip surface, and the EGFR$^{CHO\text{-}K1}$ added to the wells. The running buffer consisted of 20 mM Tris-HCl, 150 mM KCl, and 1 mg/ml bovine serum albumin (BSA), 0.005% (v/v) Tween 20, pH 7.5. Values of all constants, which were derived using a homogeneous ligand 1:1 binding model and global fits **(Experimental Section)**, indicate mean ± s.e.m. The average values are reported as mean ± s.d. and were obtained from n = 3 independent BLI experiments.

The quality of the fits was assessed using the reduced chi-squared (χ^2) and the coefficient of determination (R-squared, R^2). χ^2 quantifies the deviation between the experimental and fitted sensorgrams, while R^2 indicates how well the model predicts the binding response. Lower χ^2 and higher R^2 values indicate good agreement between the binding model and the acquired BLI data.

**EGF & EGFR$^{CHO\text{-}K1}$**					
**Repeat**	**k_{on} (×10^5 M^{-1}s^{-1})**	**k_{off} (×10^{-2} s^{-1})**	**K_D (nM)**	**χ^2**	**R^2**
**1**	1.64 ± 0.03	2.43 ± 0.01	149 ± 3	0.739	0.993
**2**	1.84 ± 0.03	2.32 ± 0.01	126 ± 2	0.525	0.994
**3**	1.97 ± 0.04	1.95 ± 0.01	98.7 ± 2.1	0.799	0.989
**Average**	1.82 ± 0.14	2.23 ± 0.21	124 ± 20		

**TGF-α & EGFR$^{CHO\text{-}K1}$**					
**Repeat**	**k_{on} (×10^5 M^{-1}s^{-1})**	**k_{off} (×10^{-2} s^{-1})**	**K_D (nM)**	**χ^2**	**R^2**
**1**	1.99 ± 0.07	2.01 ± 0.02	101 ± 4	1.985	0.975
**2**	2.08 ± 0.07	1.94 ± 0.02	92.9 ± 3.3	1.880	0.975
**3**	2.12 ± 0.07	1.98 ± 0.02	93.6 ± 3.4	1.622	0.974
**Average**	2.06 ± 0.05	1.97 ± 0.03	95.7 ± 3.5		

**HB-EGF & EGFR$^{CHO\text{-}K1}$**					
**Repeat**	**k_{on} (×10^5 M^{-1}s^{-1})**	**k_{off} (×10^{-2} s^{-1})**	**K_D (nM)**	**χ^2**	**R^2**
**1**	0.998 ± 0.027	1.66 ± 0.02	167 ± 5	0.521	0.984
**2**	1.14 ± 0.03	1.04 ± 0.01	91.8 ± 2.7	0.679	0.971
**3**	1.10 ± 0.03	1.35 ± 0.01	122 ± 3	0.846	0.981
**Average**	1.08 ± 0.06	1.35 ± 0.25	127 ± 31		

5. Kinetic and affinity constants of high-affinity growth factors with EGFR$^{CHO\text{-}K1}$ using SPR.

Supplementary Table S9. SPR-determined kinetic rate constants of association and dissociation, k_{on} and k_{off}, respectively, and equilibrium dissociation constants, K_D, of the GF-receptor interactions with the EGFR$^{CHO\text{-}K1}$ attached to the SPR chip surface, and the EGF, TGF-α, or HB-EGF added to the solution. The running buffer consisted of 20 mM Tris-HCl, 150 mM KCl, and 1 mg/ml bovine serum albumin (BSA), 0.05% Tween 20, pH 7.5. Values of all constants, which were derived using heterogeneous ligand 2:1 global fits **(Experimental Section)**, indicate mean ± s.e.m. The average values are reported as mean ± s.d. and were obtained from n = 3 independent SPR experiments.

The quality of the SPR fits was assessed using the reduced chi-squared (χ^2). χ^2 quantifies the deviation between the experimental and fitted sensorgrams. Lower χ^2 values indicate good agreement between the binding model and the acquired SPR data.

EGF & EGFR$^{CHO\text{-}K1}$

Repeat	$k_{on\text{-}1}$ (×10^6 M^{-1}s^{-1})	$k_{off\text{-}1}$ (×10^{-1} s^{-1})	$K_{D\text{-}1}$ (nM)	$k_{on\text{-}2}$ (×10^5 M^{-1}s^{-1})	$k_{off\text{-}2}$ (×10^{-1} s^{-1})	$K_{D\text{-}2}$ (nM)	χ^2
1	0.928 ± 0.006	0.273 ± 0.001	29.5	5.27 ± 0.02	2.39 ± 0.01	454	0.087
2	1.00 ± 0.01	0.258 ± 0.001	25.7	5.79 ± 0.02	2.41 ± 0.01	416	0.088
3	1.00 ± 0.01	0.283 ± 0.001	28.3	5.54 ± 0.02	2.51 ± 0.01	453	0.095
**Average**	0.977 ± 0.035	0.271 ± 0.010	27.8 ± 1.6	5.53 ± 0.21	2.44 ± 0.05	441 ± 18	

TGF-α & EGFR$^{CHO\text{-}K1}$

Repeat	$k_{on\text{-}1}$ (×10^6 M^{-1}s^{-1})	$k_{off\text{-}1}$ (×10^{-1} s^{-1})	$K_{D\text{-}1}$ (nM)	$k_{on\text{-}2}$ (×10^5 M^{-1}s^{-1})	$k_{off\text{-}2}$ (×10^{-1} s^{-1})	$K_{D\text{-}2}$ (nM)	χ^2
1	1.61 ± 0.02	1.66 ± 0.01	103	2.61 ± 0.02	4.76 ± 0.02	1820	0.0254
2	1.49 ± 0.03	2.02 ± 0.02	135	2.58 ± 0.03	5.10 ± 0.03	1979	0.0108
3	1.64 ± 0.02	2.60 ± 0.02	158	2.39 ± 0.03	5.74 ± 0.04	2401	0.0083
**Average**	1.58 ± 0.06	2.09 ± 0.39	132 ± 23	2.52 ± 0.10	5.20 ± 0.41	2067 ± 245	

HB-EGF & EGFR$^{CHO\text{-}K1}$

Repeat	$k_{on\text{-}1}$ (×10^6 M^{-1}s^{-1})	$k_{off\text{-}1}$ (×10^{-1} s^{-1})	$K_{D\text{-}1}$ (nM)	$k_{on\text{-}2}$ (×10^5 M^{-1}s^{-1})	$k_{off\text{-}2}$ (×10^{-1} s^{-1})	$K_{D\text{-}2}$ (nM)	χ^2
1	1.72 ± 0.01	0.149 ± 0.001	8.64	9.21 ± 0.05	0.770 ± 0.004	83.7	0.533
2	4.29 ± 0.07	0.275 ± 0.003	6.42	14.1 ± 0.1	1.09 ± 0.01	77.0	0.591
3	7.04 ± 0.23	0.499 ± 0.015	7.09	16.9 ± 0.2	1.39 ± 0.01	82.4	0.229
**Average**	4.35 ± 2.17	0.308 ± 0.145	7.39 ± 0.93	13.4 ± 3.2	1.08 ± 0.25	81.0 ± 2.9	

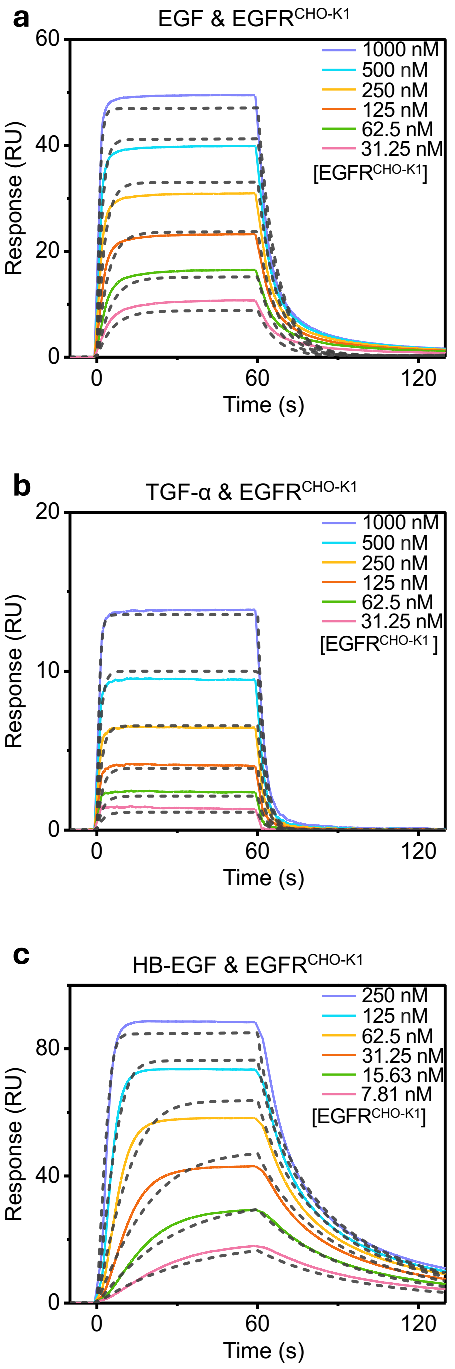

Supplementary Figure S8. SPR analyses of GF interactions with the extracellular domain of EGFR expressed in CHO-K1 cells. (a) EGF–EGFR$^{CHO\text{-}K1}$, **(b)** TGF-α–EGFR$^{CHO\text{-}K1}$, and **(c)** HB-EGF–EGFR$^{CHO\text{-}K1}$. Representative SPR sensorgrams show the association and dissociation phases. EGFR$^{CHO\text{-}K1}$ was immobilized on Cytiva CM5 sensor chips, and each GF was injected into the solution. All binding curves were globally fitted (black dotted lines) using a homogeneous 1:1 binding model. The association phase is shown in the first 60 seconds, and the dissociation phase is shown in the next 70 seconds.

Supplementary Table S10. **SPR-determined kinetic rate constants of association and dissociation, k_{on} and k_{off}, respectively, and equilibrium dissociation constants, K_D, of the growth factor-receptor interactions with the $EGFR^{CHO-K1}$ attached to the SPR chip surface and the EGF or TGF-α or HB-EGF added to the wells.** The running buffer consisted of 20 mM Tris-HCl, 150 mM KCl, and 1 mg/ml bovine serum albumin (BSA), 0.05% Tween 20, pH 7.5. Values of all constants, which were derived using 1:1 global fits **(Experimental Section)**, indicate mean ± s.e.m. The average values are reported as mean ± s.d. and were obtained from *n* = 3 independent SPR experiments.

The quality of the SPR fits was assessed using the reduced chi-squared (χ^2). χ^2 quantifies the deviation between the experimental and fitted sensorgrams. Lower χ^2 values indicate good agreement between the binding model and the acquired SPR data.

**EGF & $EGFR^{CHO-K1}$**				
**Repeat**	**k_{on} ($\times 10^5$ $M^{-1}s^{-1}$)**	**k_{off} ($\times 10^{-1}$ s^{-1})**	**K_D (nM)**	**χ^2**
**1**	5.42 ± 0.02	1.28 ± 0.01	237	0.813
**2**	5.81 ± 0.03	1.31 ± 0.01	226	0.950
**3**	5.72 ± 0.02	1.32 ± 0.01	231	0.901
**Average**	5.65 ± 0.17	1.30 ± 0.02	231 ± 5	

**TGF-α & $EGFR^{CHO-K1}$**				
**Repeat**	**k_{on} ($\times 10^5$ $M^{-1}s^{-1}$)**	**k_{off} ($\times 10^{-1}$ s^{-1})**	**K_D (nM)**	**χ^2**
**1**	8.19 ± 0.03	4.29 ± 0.01	524	0.0101
**2**	7.36 ± 0.03	3.21 ± 0.01	436	0.0432
**3**	6.17 ± 0.02	3.34 ± 0.01	541	0.0284
**Average**	7.24 ± 0.83	3.61 ± 0.48	500 ± 46	

**HB-EGF & $EGFR^{CHO-K1}$**				
**Repeat**	**k_{on} ($\times 10^5$ $M^{-1}s^{-1}$)**	**k_{off} ($\times 10^{-1}$ s^{-1})**	**K_D (nM)**	**χ^2**
**1**	30.3 ± 0.3	0.872 ± 0.009	28.7	3.17
**2**	45.4 ± 0.8	1.43 ± 0.02	31.4	1.86
**3**	54.2 ± 1.0	1.49 ± 0.03	27.6	2.96
**Average**	43.3 ± 9.9	1.26 ± 0.28	29.2 ± 1.6	

6. Kinetic and affinity constants of GF-EGFR ECD interactions reported by other research groups under different experimental circumstances.

<u>Supplementary Table S11.</u> Rate constants and affinities of EGF-EGFR ECD interactions reported by other research teams.

k_{on} ($M^{-1}s^{-1}$)	k_{off} (s^{-1})	K_D (nM)	Method	Reference
-	-	177	SPR	[12]
2.0×10^5	18×10^{-3}	90[a]	SPR[a]	[13]
1.5×10^5	2.7×10^{-3}	18[b]	SPR[b]	[13]
1.5×10^5	62×10^{-3}	410	SPR	[14]
-	-	20/550	SPR	[15]

[12]In this reference, the EGFR ECD was immobilized on the SPR sensor surface. The running buffer included 137 mM NaCl, 2.7 mM KCl, 10 mM Na_2HPO_4, and 2 mM KH_2PO_4, pH 7.4.
[a]In this reference, the human EGFR ECD was immobilized onto the SPR sensor surface. The running buffer was 10 mM HEPES, pH 7.4, 150 mM NaCl, with 0.005% (v/v) surfactant P20.[13]
[b]In this reference, the murine EGFR ECD was immobilized onto the SPR sensor surface. The running buffer was 10 mM HEPES, pH 7.4, 0.15 M NaCl, with 0.005% (v/v) surfactant P20.[13]
[14]In this reference, human EGF was immobilized onto the SPR sensor surface and EGFR ECD was added to the wells. The running buffer was 10 mM HEPES, pH 7.4, 150 mM NaCl, 3.4 mM EDTA, and 0.005% (v/v) surfactant P20 (HBS).
[15]Here, EGFR was attached to the sensor surface. The running buffer of the SPR experiment included 10 mM HEPES, pH 7.4, 3.4 mM EDTA, 150 mM NaCl, and 0.005% (v/v) Tween 20.

<u>Supplementary Table S12.</u> Rate constants and affinities of TGFα-EGFR ECD interactions reported by other research teams.

k_{on} (10^5 $M^{-1}s^{-1}$)	k_{off} (10^{-2} s^{-1})	K_D (nM)	Method	Reference
2.9/0.27	6.7/0.35	233/132	SPR	[16]

[16]Here, TGF-α was immobilized on the SPR sensor surface. The running buffer was 10 mM HEPES, pH 8.0, 150 mM NaCl, with 0.05% Tween 20.

<u>Supplementary Table S13.</u> Rate constants and affinities of HB-EGF-EGFR ECD interactions reported by other research teams.

k_{on} (10^7 $M^{-1}s^{-1}$)[17]	k_{off} (10^{-3} s^{-1})[17]	K_D (nM)[18]	Method	Reference
6.3	7.5	7.1	MD simulations	[17, 18]
-	-	7.1	Radiolabeling assay	[19]

[19]This value is determined by a radiolabeling binding assay in competitive binding interactions against ^{125}I[EGF].

7. Mapping of N-glycosylation sites of EGFR ECD and the specific location of N151 within the EGF binding site.

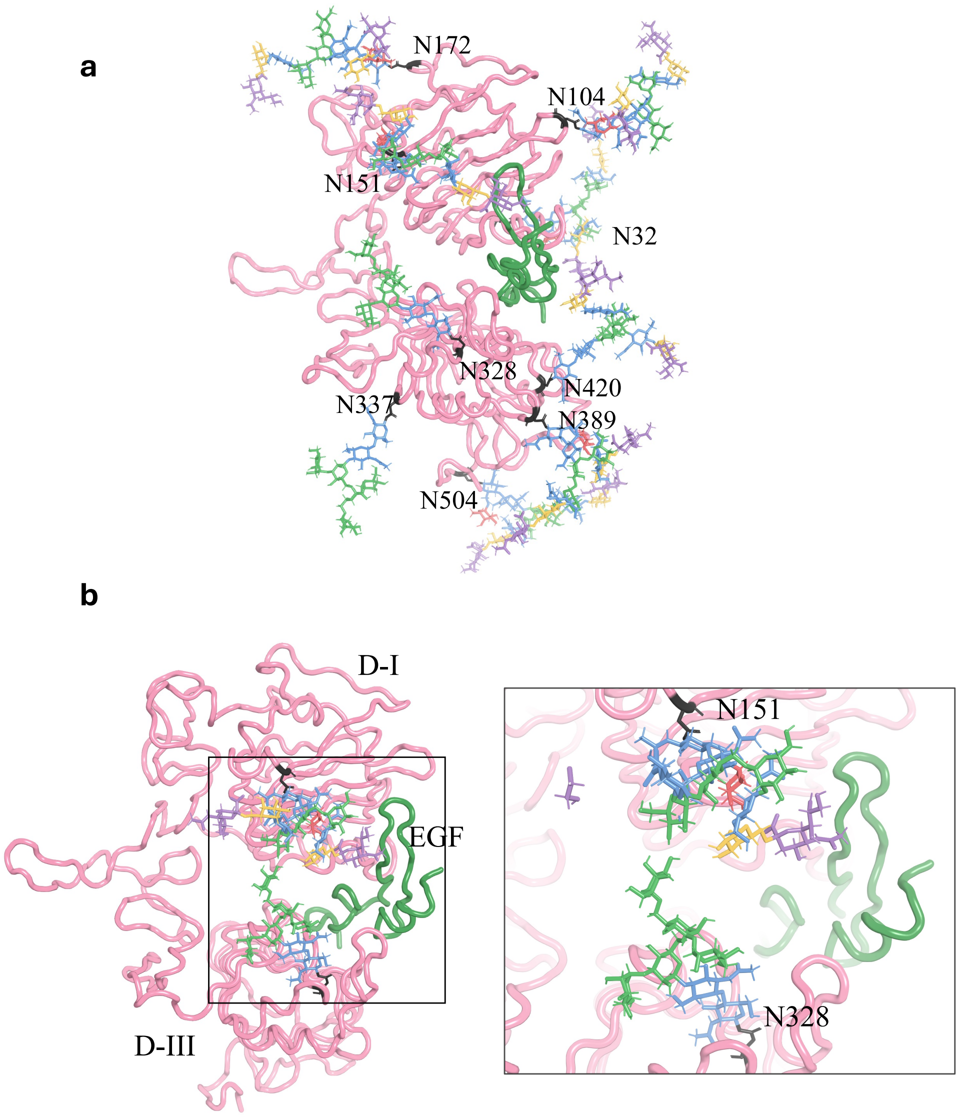

Supplementary Figure S9. Cartoon and zoomed-in view of N-glycosylation sites on EGFR ECD. (a) Overall structure of the EGFR ECD in complex with EGF. They are shown in pink and green, respectively. N-linked glycans are represented as sticks and colored according to monosaccharide type: N-acetylglucosamine (GlcNAc, blue), mannose (green), galactose (yellow), fucose (red), and sialic acid (purple). Glycan compositions were assigned based on mass spectrometry data of EGFR ECD expressed in CHO-K1 cells.[9] **(b)** Zoomed-in view of the N-linked glycans at Asn151 and Asn328. Residues Asn151 and Asn328 are highlighted in black, while the surrounding protein regions are rendered semi-transparently to emphasize the glycan–protein interface. The structures were generated from PDB entry 1IVO[20] and visualized with GlycoShape[8] and PyMOL. The EGFR ECD structure only included residues 1-504.

8. Kinetic and affinity constants of high-affinity GFs with the N151A mutant of EGFR using BLI.

<u>Supplementary Table S14.</u> BLI-determined kinetic rate constants of association and dissociation, k_{on} and k_{off}, respectively, and equilibrium dissociation constants, K_D, of the GF-receptor interactions with the EGF, TGF-α, or HB-EGF attached to the BLI chip surface and the $EGFR^{N151A}$ added to the wells. The running buffer consisted of 20 mM Tris-HCl, 150 mM KCl, and 1 mg/ml bovine serum albumin (BSA), 0.005% (v/v) Tween 20, pH 7.5. Values of all constants, which were derived using 2:1 heterogeneous ligand global fits **(Experimental Section)**, indicate mean ± s.e.m. The average values are reported as mean ± s.d. and were obtained from *n* = 3 independent BLI experiments.

The quality of the fits was assessed using the reduced chi-squared (χ^2) and the coefficient of determination (R-squared, R^2). χ^2 quantifies the deviation between the experimental and fitted sensorgrams, while R^2 indicates how well the model predicts the binding response. Lower χ^2 and higher R^2 values indicate good agreement between the binding model and the acquired BLI data.

**EGF & $EGFR^{N151A}$**								
**Repeat**	**k_{on-1} ($\times 10^5$ $M^{-1}s^{-1}$)**	**k_{off-1} ($\times 10^{-2}$ s^{-1})**	**K_{D-1} (nM)**	**k_{on-2} ($\times 10^4$ $M^{-1}s^{-1}$)**	**k_{off-2} ($\times 10^{-2}$ s^{-1})**	**K_{D-2} (nM)**	**χ^2**	**R^2**
**1**	3.79 ± 0.04	4.00 ± 0.02	106 ± 1	5.92 ± 0.08	0.656 ± 0.008	111 ± 2	0.0370	0.999
**2**	4.35 ± 0.05	4.23 ± 0.03	97.1 ± 0.1	7.67 ± 0.09	0.818 ± 0.008	107 ± 2	0.0228	0.999
**3**	3.91 ± 0.04	4.04 ± 0.02	103 ± 1	5.77 ± 0.09	0.672 ± 0.005	116 ± 2	0.0629	0.999
**Average**	4.02 ± 0.24	4.09 ± 0.10	102 ± 4	6.45 ± 0.86	0.715 ± 0.073	111 ± 4		

**TGF-α & $EGFR^{N151A}$**								
**Repeat**	**k_{on-1} ($\times 10^5$ $M^{-1}s^{-1}$)**	**k_{off-1} ($\times 10^{-2}$ s^{-1})**	**K_{D-1} (nM)**	**k_{on-2} ($\times 10^4$ $M^{-1}s^{-1}$)**	**k_{off-2} ($\times 10^{-2}$ s^{-1})**	**K_{D-2} (nM)**	**χ^2**	**R^2**
**1**	5.96 ± 0.12	7.39 ± 0.07	124 ± 3	5.31 ± 0.06	0.731 ± 0.006	138 ± 2	0.137	0.999
**2**	6.11 ± 0.13	7.35 ± 0.07	120 ± 1	4.84 ± 0.06	0.619 ± 0.005	128 ± 2	0.0610	0.999
**3**	5.35 ± 0.10	6.76 ± 0.05	126 ± 1	4.22 ± 0.05	0.549 ± 0.004	130 ± 2	0.0783	0.999
**Average**	5.81 ± 0.33	7.16 ± 0.29	124 ± 3	4.79 ± 0.44	0.633 ± 0.075	132 ± 4		

**HB-EGF & $EGFR^{N151A}$**								
**Repeat**	**k_{on-1} ($\times 10^5$ $M^{-1}s^{-1}$)**	**k_{off-1} ($\times 10^{-2}$ s^{-1})**	**K_{D-1} (nM)**	**k_{on-2} ($\times 10^4$ $M^{-1}s^{-1}$)**	**k_{off-2} ($\times 10^{-2}$ s^{-1})**	**K_{D-2} (nM)**	**χ^2**	**R^2**
**1**	2.61 ± 0.04	4.40 ± 0.04	169 ± 1	3.68 ± 0.04	0.505 ± 0.006	137 ± 2	0.0498	0.999
**2**	2.79 ± 0.05	4.46 ± 0.04	160 ± 1	3.73 ± 0.05	0.408 ± 0.006	109 ± 2	0.0550	0.999
**3**	2.78 ± 0.07	4.74 ± 0.07	170 ± 1	3.33 ± 0.07	0.242 ± 0.008	72.6 ± 2.8	0.0162	0.998
**Average**	2.73 ± 0.08	4.53 ± 0.15	166 ± 5	3.58 ± 0.18	0.385 ± 0.109	106 ± 26		

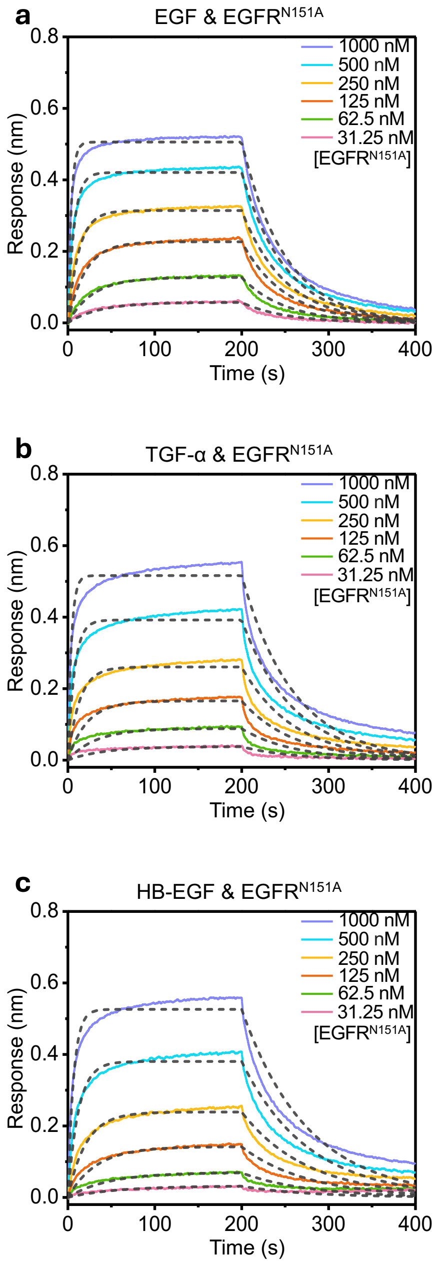

<u>Supplementary Figure S10.</u> BLI assays measuring the interactions between GFs with the extracellular domain of EGFR^{N151A} expressed by the CHO-K1 cell line. (a) EGF–EGFR^{N151A}. **(b)** TGF-α–EGFR^{N151A}. **(c)** HB-EGF–EGFR^{N151A}. Representative BLI sensorgrams show the association and dissociation phases. For each panel, 10 nM biotinylated growth factor was loaded onto SA sensors for 10 min, then dipped into buffers containing six twofold serial dilutions of EGFR^{N151A} from 1000 nM to 31.25 nM for the association phase. Sensors were subsequently transferred to EGFR^{N151A}-free buffer for the dissociation phase. Dashed lines indicate fits of BLI sensorgrams showing interactions between EGFR^{N151A} and its high-affinity GFs. The binding curves were fitted (black dotted lines) using the FortéBio Octet Data Analysis software (FortéBio) with a standard 1:1 global fitting model. The association phase is represented by the first 200 seconds, and the dissociation is indicated by the second 200 seconds.

<u>Supplementary Table S15.</u> BLI-determined kinetic rate constants of association and dissociation, k_{on} and k_{off}, respectively, and equilibrium dissociation constants, K_D, of the GF-receptor interactions with EGF, TGFα, HB-EGF attached to the BLI chip surface, and the EGFR^{N151A} added to the wells. The running buffer consisted of 20 mM Tris-HCl, 150 mM KCl, and 1 mg/ml bovine serum albumin (BSA), 0.005% (v/v) Tween 20, pH 7.5. Values of all constants, which were derived using a standard 1:1 binding model and global fits **(Experimental Section)**, indicate mean ± s.e.m. The average values are reported as mean ± s.d. and were obtained from $n = 3$ independent BLI experiments.

The quality of the fits was assessed using the reduced chi-squared (χ^2) and the coefficient of determination (R-squared, R^2). χ^2 quantifies the deviation between the experimental and fitted sensorgrams, while R^2 indicates how well the model predicts the binding response. Lower χ^2 and higher R^2 values indicate good agreement between the binding model and the acquired BLI data.

**EGF & EGFR^{N151A}**					
**Repeat**	**k_{on} (×10^5 M^{-1}s^{-1})**	**k_{off} (×10^{-2} s^{-1})**	**K_D (nM)**	**χ^2**	**R^2**
**1**	2.30 ± 0.04	1.93 ± 0.01	83.7 ± 1.6	0.805	0.991
**2**	2.43 ± 0.04	1.99 ± 0.01	82.0 ± 1.5	0.444	0.993
**3**	2.43 ± 0.04	1.99 ± 0.01	81.9 ± 1.5	0.639	0.991
**Average**	2.39 ± 0.06	1.97 ± 0.03	82.5 ± 0.8		

**TGF-α & EGFR^{N151A}**					
**Repeat**	**k_{on} (×10^5 M^{-1}s^{-1})**	**k_{off} (×10^{-2} s^{-1})**	**K_D (nM)**	**χ^2**	**R^2**
**1**	2.28 ± 0.07	1.77 ± 0.02	77.7 ± 2.4	3.002	0.980
**2**	2.33 ± 0.07	1.67 ± 0.01	71.8 ± 2.3	1.312	0.978
**3**	2.22 ± 0.07	1.67 ± 0.01	75.1 ± 2.4	1.454	0.977
**Average**	2.28 ± 0.04	1.70 ± 0.05	74.9 ± 2.4		

**HB-EGF & EGFR^{N151A}**					
**Repeat**	**k_{on} (×10^5 M^{-1}s^{-1})**	**k_{off} (×10^{-2} s^{-1})**	**K_D (nM)**	**χ^2**	**R^2**
**1**	1.29 ± 0.03	1.40 ± 0.01	108 ± 2	0.973	0.986
**2**	1.39 ± 0.03	1.32 ± 0.01	94.5 ± 2.3	0.989	0.983
**3**	1.43 ± 0.05	1.02 ± 0.01	71.6 ± 2.4	0.451	0.961
**Average**	1.37 ± 0.06	1.25 ± 0.16	91.4 ± 15.1		

9. Kinetic and affinity constants of high-affinity GFs with EGFR^{N151A} using SPR.

<u>**Supplementary Table S16.**</u> **SPR-determined kinetic rate constants of association and dissociation, k_{on} and k_{off}, respectively, and equilibrium dissociation constants, K_D, of the GF-receptor interactions with the EGFR^{N151A} attached to the SPR chip surface and the EGF, TGF-α, or HB-EGF added to the solution.** The running buffer consisted of 20 mM Tris-HCl, 150 mM KCl, and 1 mg/ml bovine serum albumin (BSA), 0.05% Tween 20, pH 7.5. Values of all constants, which were derived using a heterogeneous ligand 2:1 binding model and global fits **(Experimental Section)**, indicate mean ± s.e.m. The average values are reported as mean ± s.d. and were obtained from n = 3 independent SPR experiments.

The quality of the SPR fits was assessed using the reduced chi-squared (χ^2). χ^2 quantifies the deviation between the experimental and fitted sensorgrams. Lower χ^2 values indicate good agreement between the binding model and the acquired SPR data.

EGF & EGFR^{N151A}

Repeat	k_{on-1} (×10^6 M^{-1}s^{-1})	k_{off-1} (×10^{-1} s^{-1})	K_{D-1} (nM)	k_{on-2} (×10^5 M^{-1}s^{-1})	k_{off-2} (×10^{-1} s^{-1})	K_{D-2} (nM)	χ^2
**1**	0.889 ± 0.006	0.294 ± 0.001	33.1	5.86 ± 0.02	2.60 ± 0.01	443	0.030
**2**	1.05 ± 0.01	0.351 ± 0.002	33.4	5.93 ± 0.02	2.06 ± 0.01	347	0.037
**3**	1.48 ± 0.01	0.320 ± 0.001	21.6	6.42 ± 0.02	2.61 ± 0.01	407	0.024
**Average**	1.14 ± 0.25	0.322 ± 0.023	29.4 ± 5.5	6.07 ± 0.25	2.42 ± 0.26	399 ± 40	

TGF-α & EGFR^{N151A}

Repeat	k_{on-1} (×10^6 M^{-1}s^{-1})	k_{off-1} (×10^{-1} s^{-1})	K_{D-1} (nM)	k_{on-2} (×10^5 M^{-1}s^{-1})	k_{off-2} (×10^{-1} s^{-1})	K_{D-2} (nM)	χ^2
**1**	1.28 ± 0.01	1.75 ± 0.01	137	3.25 ± 0.02	5.83 ± 0.03	1791	0.0236
**2**	1.11 ± 0.01	1.47 ± 0.01	133	3.80 ± 0.02	5.64 ± 0.04	1483	0.0326
**3**	2.33 ± 0.03	2.95 ± 0.02	127	3.02 ± 0.03	6.26 ± 0.04	2073	0.00536
**Average**	1.57 ± 0.54	2.06 ± 0.64	132 ± 4	3.36 ± 0.33	5.91 ± 0.26	1783 ± 241	

HB-EGF & EGFR^{N151A}

Repeat	k_{on-1} (×10^6 M^{-1}s^{-1})	k_{off-1} (×10^{-1} s^{-1})	K_{D-1} (nM)	k_{on-2} (×10^5 M^{-1}s^{-1})	k_{off-2} (×10^{-1} s^{-1})	K_{D-2} (nM)	χ^2
**1**	8.68 ± 0.30	0.464 ± 0.015	5.34	19.3 ± 0.3	1.51 ± 0.02	78.1	0.389
**2**	8.51 ± 0.28	0.467 ± 0.015	5.48	17.0 ± 0.2	1.54 ± 0.02	90.6	0.147
**3**	29.1 ± 1.6	0.591 ± 0.030	2.03	35.7 ± 0.5	1.65 ± 0.02	46.2	0.177
**Average**	15.4 ± 9.6	0.507 ± 0.059	4.29 ± 1.59	24.0 ± 8.3	1.57 ± 0.06	71.6 ± 18.7	

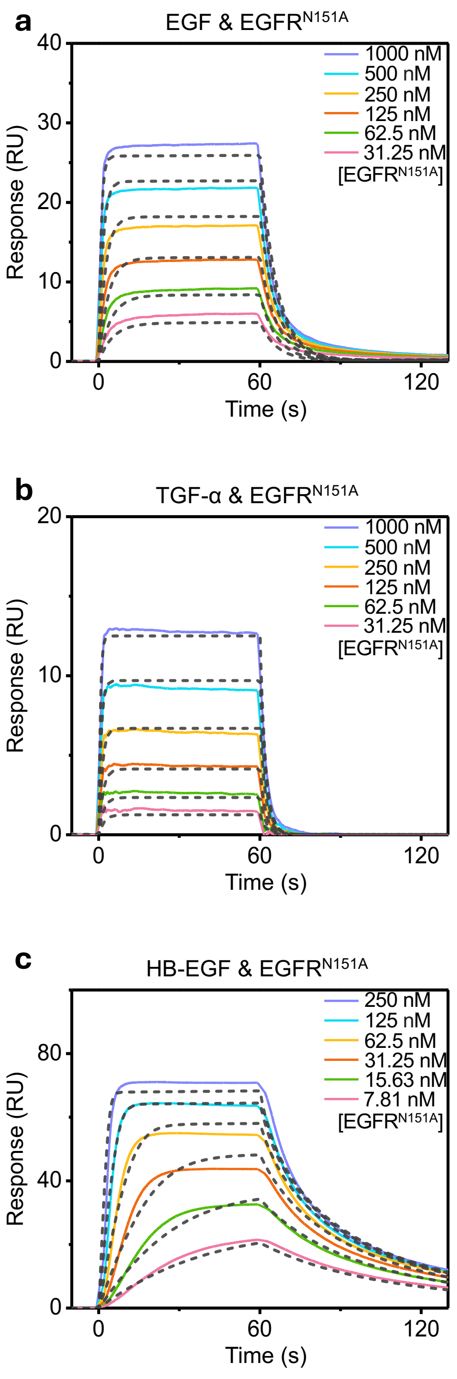

<u>Supplementary Figure S11.</u> SPR analyses of the interactions between individual GFs and the extracellular domain of $EGFR^{N151A}$ expressed in CHO-K1 cells. (a) EGF–$EGFR^{N151A}$. **(b)** TGF-α– $EGFR^{N151A}$. and **(c)** HB-EGF– $EGFR^{N151A}$. Representative SPR sensorgrams show the association and dissociation phases. $EGFR^{N151A}$ was immobilized on Cytiva CM5 sensor chips, and individual high-affinity GFs were injected into the solution. All binding curves were globally fitted (black dotted lines) using a standard 1:1 binding model. The association phase is represented by the first 60 seconds, and the dissociation is indicated by the second 70 seconds.

Supplementary Table S17. SPR-determined kinetic rate constants of association and dissociation, k_{on} and k_{off}, respectively, and equilibrium dissociation constants, K_D, of the GF-receptor interactions with the EGFR^{N151A} attached to the SPR chip surface and EGF, TGF-α, or HB-EGF added to the solution. The running buffer consisted of 20 mM Tris-HCl, 150 mM KCl, and 1 mg/ml bovine serum albumin (BSA), 0.05% Tween 20, pH 7.5. Values of all constants, which were derived using a standard 1:1 binding model and global fits **(Experimental Section)**, indicate mean ± s.e.m. The average values are reported as mean ± s.d. and were obtained from $n = 3$ independent SPR experiments.

The quality of the SPR fits was assessed using the reduced chi-squared (χ^2). χ^2 quantifies the deviation between the experimental and fitted sensorgrams. Lower χ^2 values indicate good agreement between the binding model and the acquired SPR data.

**EGF & EGFR^{N151A}**				
**Repeat**	**k_{on} ($\times 10^5$ M^{-1}s^{-1})**	**k_{off} ($\times 10^{-1}$ s^{-1})**	**K_D (nM)**	**χ^2**
**1**	5.78 ± 0.02	1.43 ± 0.01	247	0.241
**2**	6.27 ± 0.03	1.45 ± 0.01	232	0.220
**3**	6.95 ± 0.03	1.46 ± 0.01	211	0.231
**Average**	6.33 ± 0.48	1.45 ± 0.02	230 ± 15	

**TGF-α & EGFR^{N151A}**				
**Repeat**	**k_{on} ($\times 10^5$ M^{-1}s^{-1})**	**k_{off} ($\times 10^{-1}$ s^{-1})**	**K_D (nM)**	**χ^2**
**1**	10.1 ± 0.1	4.73 ± 0.02	466	0.00993
**2**	8.04 ± 0.02	3.13 ± 0.01	389	0.0468
**3**	7.34 ± 0.02	2.99 ± 0.01	408	0.0548
**Average**	8.51 ± 1.19	3.62 ± 0.79	421 ± 33	

**HB-EGF & EGFR^{N151A}**				
**Repeat**	**k_{on} ($\times 10^5$ M^{-1}s^{-1})**	**k_{off} ($\times 10^{-1}$ s^{-1})**	**K_D (nM)**	**χ^2**
**1**	62.5 ± 1.1	1.38 ± 0.02	22.1	4.72
**2**	82.9 ± 2.4	2.07 ± 0.06	24.9	3.66
**3**	89.9 ± 2.9	2.11 ± 0.07	23.5	3.75
**Average**	78.4 ± 11.6	1.85 ± 0.33	23.5 ± 1.2	

10. Kinetic and affinity constants of high-affinity growth factors with the PNGase F-treated EGFR isoform expressed in the CHO-K1 cell line ($^{DG}EGFR^{CHO\text{-}K1}$) using BLI.

<u>**Supplementary Table S18.**</u> **BLI-determined kinetic rate constants of association and dissociation, k_{on} and k_{off}, respectively, and equilibrium dissociation constants, K_D, for the GF-receptor interactions with EGF, TGF-α, or HB-EGF attached to the BLI chip surface and $^{DG}EGFR^{CHO\text{-}K1}$ added to the wells.** The running buffer consisted of 20 mM Tris-HCl, 150 mM KCl, 1 mg/ml bovine serum albumin (BSA), 0.005% (v/v) Tween 20, and pH 7.5. Values for all constants, derived using a heterogeneous ligand 2:1 binding model and global fits **(Experimental Section)**, are reported as mean ± s.e.m. Average values are reported as mean ± s.d. and were obtained from n = 3 independent BLI experiments.

The quality of the fits was assessed using the reduced chi-squared (χ^2) and the coefficient of determination (R-squared, R^2). χ^2 quantifies the deviation between the experimental and fitted sensorgrams, while R^2 indicates how well the model predicts the binding response. Lower χ^2 and higher R^2 values indicate good agreement between the binding model and the acquired BLI data.

EGF & $^{DG}EGFR^{CHO\text{-}K1}$								
**Repeat**	$k_{on\text{-}1}$ ($\times 10^5$ $M^{-1}s^{-1}$)	$k_{off\text{-}1}$ ($\times 10^{-2}$ s^{-1})	$K_{D\text{-}1}$ (nM)	$k_{on\text{-}2}$ ($\times 10^4$ $M^{-1}s^{-1}$)	$k_{off\text{-}2}$ ($\times 10^{-2}$ s^{-1})	$K_{D\text{-}2}$ (nM)	χ^2	R^2
**1**	0.323 ± 0.034	12.8 ± 0.2	3971 ± 261	0.804 ± 0.126	0.573 ± 0.027	712 ± 117	0.009	0.998
**2**	0.403 ± 0.053	13.0 ± 0.4	3234 ± 324	1.07 ± 0.20	1.96 ± 0.11	1830 ± 363	0.068	0.994
**3**	0.514 ± 0.055	12.0 ± 0.4	2328 ± 183	1.07 ± 0.21	2.10 ± 0.13	1974 ± 407	0.081	0.995
**Average**	0.413 ± 0.078	12.6 ± 0.5	3178 ± 672	0.981 ± 0.125	1.55 ± 0.69	1505 ± 564		

TGF-α & $^{DG}EGFR^{CHO\text{-}K1}$								
**Repeat**	$k_{on\text{-}1}$ ($\times 10^5$ $M^{-1}s^{-1}$)	$k_{off\text{-}1}$ ($\times 10^{-2}$ s^{-1})	$K_{D\text{-}1}$ (nM)	$k_{on\text{-}2}$ ($\times 10^4$ $M^{-1}s^{-1}$)	$k_{off\text{-}2}$ ($\times 10^{-2}$ s^{-1})	$K_{D\text{-}2}$ (nM)	χ^2	R^2
**1**	1.31 ± 0.04	12.1 ± 0.1	926 ± 10	2.14 ± 0.04	1.06 ± 0.01	494 ± 10	0.023	0.999
**2**	1.67 ± 0.04	12.4 ± 0.1	747 ± 6	2.03 ± 0.04	1.15 ± 0.01	566 ± 11	0.049	0.999
**3**	1.65 ± 0.04	12.4 ± 0.1	755 ± 5	1.98 ± 0.03	1.11 ± 0.01	560 ± 9	0.028	0.999
**Average**	1.54 ± 0.17	12.3 ± 0.2	810 ± 83	2.05 ± 0.07	1.10 ± 0.04	540 ± 33		

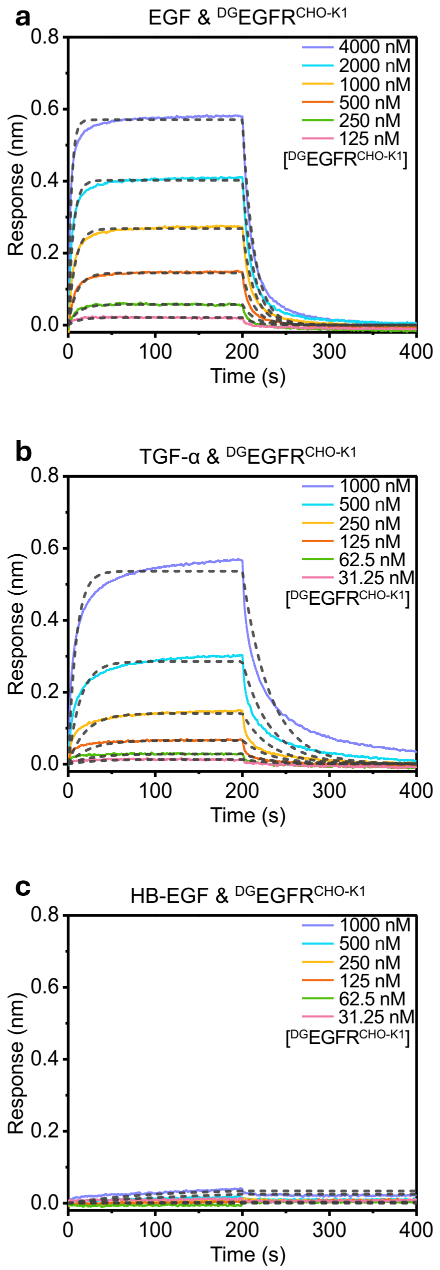

Supplementary Figure S12. BLI assays measuring interactions between GFs and the EGFR ECD after PNGase-driven extensive deglycosylation. (a) EGF–^{DG}EGFR^{CHO-K1}. **(b)** TGF-α–^{DG}EGFR^{CHO-K1}. **(c)** HB-EGF–^{DG}EGFR^{CHO-K1}. Representative BLI sensorgrams show the association and dissociation phases. For each panel, 10 nM biotinylated GF was loaded onto streptavidin-coated BLI sensors for 10 min and then dipped into buffers containing six twofold serial dilutions ^{DG}EGFR^{CHO-K1} for the association phase. Sensors were subsequently transferred to ^{DG}EGFR^{CHO-K1}-free buffer for the dissociation phase. Dashed lines indicate fits of BLI sensorgrams showing interactions between ^{DG}EGFR^{CHO-K1} and its high-affinity GFs. The binding curves were fitted (black dotted lines) using the FortéBio Octet Data Analysis software (FortéBio) with a 1:1 global fitting model. The association phase is represented by the first 200 seconds, and the dissociation is indicated by the second 200 seconds.

Supplementary Table S19. BLI-determined kinetic rate constants of association and dissociation, k_{on} and k_{off}, respectively, and equilibrium dissociation constants, K_D, of the GF-receptor interactions with EGF, TGF-α, or HB-EGF attached to the BLI chip surface and $^{DG}EGFR^{CHO-K1}$ added to the wells. The running buffer consisted of 20 mM Tris-HCl, 150 mM KCl, and 1 mg/ml bovine serum albumin (BSA), 0.005% (v/v) Tween 20, pH 7.5. Values of all constants, which were derived using 1:1 global fits **(Experimental Section)**, indicate mean ± s.e.m. The average values are reported as mean ± s.d. They were obtained from $n = 3$ independent BLI experiments.

The quality of the fits was assessed using the reduced chi-squared (χ^2) and the coefficient of determination (R-squared, R^2). χ^2 quantifies the deviation between the experimental and fitted sensorgrams, while R^2 indicates how well the model predicts the binding response. Lower χ^2 and higher R^2 values indicate good agreement between the binding model and the acquired BLI data.

EGF & $^{DG}EGFR^{CHO-K1}$					
**Repeat**	k_{on} ($\times 10^5$ $M^{-1}s^{-1}$)	k_{off} ($\times 10^{-2}$ s^{-1})	K_D (nM)	χ^2	R^2
**1**	0.477 ± 0.035	7.16 ± 0.13	1500 ± 114	0.037	0.986
**2**	0.386 ± 0.025	7.91 ± 0.10	2050 ± 133	0.082	0.993
**3**	0.424 ± 0.022	7.33 ± 0.09	1728 ± 92	0.083	0.994
**Average**	0.429 ± 0.037	7.47 ± 0.32	1759 ± 226		

TGF-α & $^{DG}EGFR^{CHO-K1}$					
**Repeat**	k_{on} ($\times 10^5$ $M^{-1}s^{-1}$)	k_{off} ($\times 10^{-2}$ s^{-1})	K_D (nM)	χ^2	R^2
**1**	0.769 ± 0.028	3.00 ± 0.04	391 ± 15	0.490	0.984
**2**	0.784 ± 0.024	3.17 ± 0.03	404 ± 13	0.795	0.986
**3**	0.799 ± 0.024	3.16 ± 0.03	396 ± 13	0.610	0.986
**Average**	0.784 ± 0.012	3.11 ± 0.08	397 ± 5		

11. The negative-control experiment for demonstrating the functional state of $^{DG}EGFR^{CHO\text{-}K1}$ using BLI.

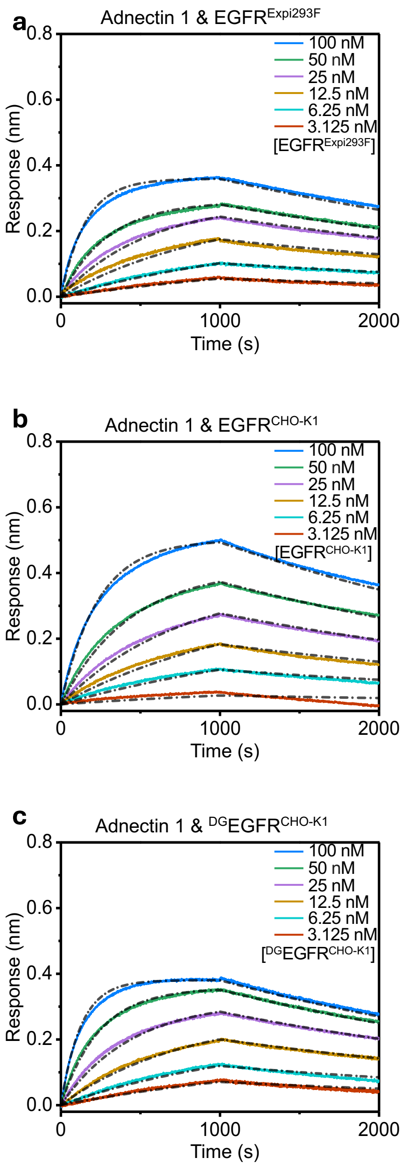

Supplementary Figure S13. The negative-control BLI experiment demonstrates that the PNGase F-treated EGFR isoform remains functional. 50 nM biotinylated EGFR monobody (Adnectin 1) was immobilized on streptavidin-coated BLI sensors. The sensors were dipped into wells containing different concentrations of EGFR. **(a)** Sensorgrams were obtained using six different $EGFR^{Expi293F}$ concentrations. **(b)** Sensorgrams were obtained using six different $EGFR^{CHO\text{-}K1}$ concentrations. **(c)** Sensorgrams were obtained using six different $^{DG}EGFR^{CHO\text{-}K1}$ concentrations. The association phase is represented by the first 1000 seconds, and the dissociation is indicated by the second 1000 seconds. The black dotted lines represent global fits using a standard 1:1 binding model.

<u>Supplementary Table S20.</u> BLI-determined kinetic rate constants of association and dissociation, k_{on} and k_{off}, respectively, and equilibrium dissociation constants, K_D, of the Adnectin 1-EGFR ECD interactions. Biotinylated Adnectin 1 was attached to the BLI sensor surface, and EGFR was added to the wells. The running buffer consisted of 20 mM Tris-HCl, 150 mM KCl, and 1 mg/ml bovine serum albumin (BSA), 0.005% Tween 20, pH 7.5. Values of all constants were derived using a standard 1:1 binding model and global fits **(Experimental Section)**. The average values are reported as mean ± s.d. They were obtained from $n = 3$ independent BLI experiments.

**Interaction partner**	**k_{on} ($\times 10^4$ $M^{-1}s^{-1}$)**	**k_{off} ($\times 10^{-4}$ s^{-1})**	**K_D (nM)**
Adnectin 1 & $EGFR^{Expi293F}$	6.16 ± 1.25	3.15 ± 0.56	5.14 ± 0.23
Adnectin 1 & $EGFR^{CHO\text{-}K1}$	5.08 ± 1.13	3.11 ± 0.16	6.39 ± 1.26
Adnectin 1 & $^{DG}EGFR^{CHO\text{-}K1}$	7.07 ± 1.47	3.14 ± 0.56	4.54 ± 0.41

12. Kinetic and affinity constants of high-affinity GFs with $^{DG}EGFR^{CHO\text{-}K1}$ using SPR.

Supplementary Table S21. SPR-determined kinetic rate constants of association and dissociation, k_{on} and k_{off}, respectively, and equilibrium dissociation constants, K_D, of the GF-receptor interactions with the $^{DG}EGFR^{CHO\text{-}K1}$ attached to the SPR chip surface and EGF, TGF-α, or HB-EGF added to the solution. The running buffer consisted of 20 mM Tris-HCl, 150 mM KCl, and 1 mg/ml bovine serum albumin (BSA), 0.05% Tween 20, pH 7.5. Values of all constants, which were derived using a heterogeneous ligand 2:1 binding model and global fits **(Experimental Section)**, indicate mean ± s.e.m. The average values are reported as mean ± s.d. They were obtained from $n = 3$ independent SPR experiments. Substate "1" contributed less than 2% to the total response. Hence, the kinetic and equilibrium constants were not determined (ND) with statistically significant accuracy.

The quality of the SPR fits was assessed using the reduced chi-squared (χ^2). χ^2 quantifies the deviation between the experimental and fitted sensorgrams. Lower χ^2 values indicate good agreement between the binding model and the acquired SPR data.

**EGF & $^{DG}EGFR^{CHO\text{-}K1}$**							
**Repeat**	**$k_{on\text{-}1}$ (×10^6 M^{-1}s^{-1})**	**$k_{off\text{-}1}$ (×10^{-1} s^{-1})**	**$K_{D\text{-}1}$ (nM)**	**$k_{on\text{-}2}$ (×10^5 M^{-1}s^{-1})**	**$k_{off\text{-}2}$ (×10^{-1} s^{-1})**	**$K_{D\text{-}2}$ (nM)**	**χ^2**
**1**	ND	ND	ND	3.24 ± 0.01	2.23 ± 0.01	689	0.0493
**2**	ND	ND	ND	3.12 ± 0.26	2.59 ± 0.11	829	5.2141
**3**	ND	ND	ND	3.97 ± 0.02	3.61 ± 0.02	908	0.0116
**Average**	ND	ND	ND	3.45 ± 0.38	2.81 ± 0.58	808 ± 91	

**TGF-α & $^{DG}EGFR^{CHO\text{-}K1}$**							
**Repeat**	**$k_{on\text{-}1}$ (×10^6 M^{-1}s^{-1})**	**$k_{off\text{-}1}$ (×10^{-1} s^{-1})**	**$K_{D\text{-}1}$ (nM)**	**$k_{on\text{-}2}$ (×10^5 M^{-1}s^{-1})**	**$k_{off\text{-}2}$ (×10^{-1} s^{-1})**	**$K_{D\text{-}2}$ (nM)**	**χ^2**
**1**	ND	ND	ND	2.67 ± 0.01	5.72 ± 0.01	2146	0.0034
**2**	ND	ND	ND	2.98 ± 0.01	5.90 ± 0.01	1979	0.0017
**3**	ND	ND	ND	2.96 ± 0.01	5.33 ± 0.01	1800	0.0016
**Average**	ND	ND	ND	2.87 ± 0.14	5.65 ± 0.24	1975 ± 141	

**HB-EGF & $^{DG}EGFR^{CHO\text{-}K1}$**							
**Repeat**	**$k_{on\text{-}1}$ (×10^6 M^{-1}s^{-1})**	**$k_{off\text{-}1}$ (×10^{-1} s^{-1})**	**$K_{D\text{-}1}$ (nM)**	**$k_{on\text{-}2}$ (×10^5 M^{-1}s^{-1})**	**$k_{off\text{-}2}$ (×10^{-1} s^{-1})**	**$K_{D\text{-}2}$ (nM)**	**χ^2**
**1**	ND	ND	ND	0.350 ± 0.035	0.200 ± 0.005	572	0.0346
**2**	ND	ND	ND	6.05 ± 0.02	1.68 ± 0.01	277	0.0375
**3**	ND	ND	ND	9.10 ± 0.03	1.69 ± 0.01	185	0.0172
**Average**	ND	ND	ND	5.17 ± 3.63	1.19 ± 0.70	345 ± 165	

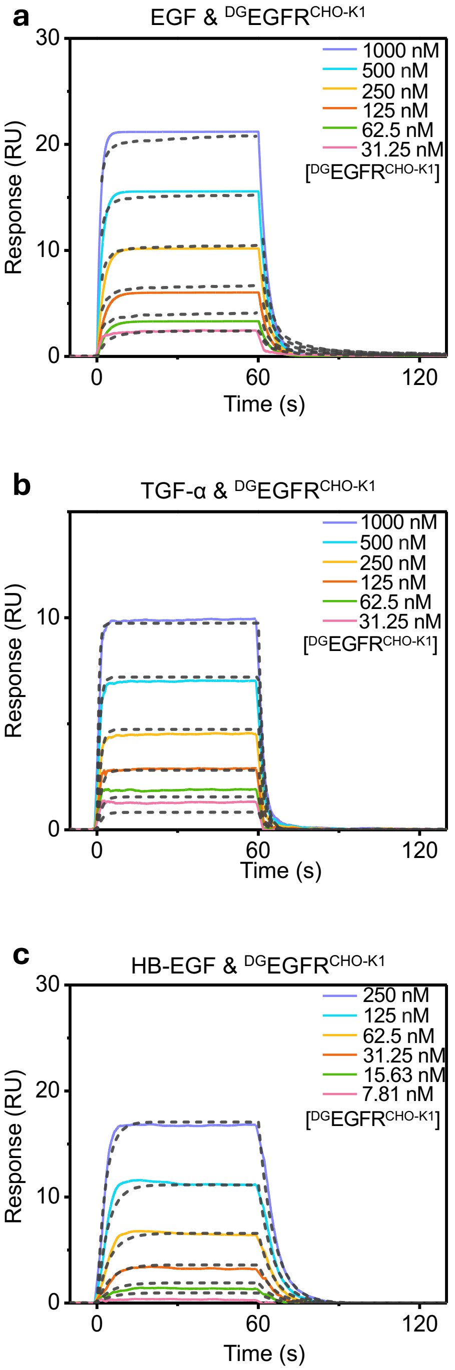

Supplementary Figure S14. SPR analyses of interactions between individual GFs and ^{DG}EGFR$^{CHO\text{-}K1}$ expressed in CHO-K1 cells. (a) EGF–^{DG}EGFR$^{CHO\text{-}K1}$, **(b)** TGF-α–^{DG}EGFR$^{CHO\text{-}K1}$, and **(c)** HB-EGF–^{DG}EGFR$^{CHO\text{-}K1}$. Representative SPR sensorgrams show the association and dissociation phases. ^{DG}EGFR$^{CHO\text{-}K1}$ was immobilized on Cytiva CM5 sensor chips, and GFs were injected into the solution. All binding curves were globally fitted (black dotted lines) using a standard 1:1 interaction model. The association phase is represented by the first 60 seconds, and the dissociation is indicated by the second 70 seconds.

Supplementary Table S22. SPR-determined kinetic rate constants of association and dissociation, k_{on} and k_{off}, respectively, and equilibrium dissociation constants, K_D, of the GF-receptor interactions with the $^{DG}EGFR^{CHO\text{-}K1}$ attached to the SPR chip surface and EGF, TGF-α, or HB-EGF added to the solution. The running buffer consisted of 20 mM Tris-HCl, 150 mM KCl, and 1 mg/ml bovine serum albumin (BSA), 0.05% Tween 20, pH 7.5. Values of all constants, which were derived using a standard 1:1 interaction model and global fits **(Experimental Section)**, indicate mean ± s.e.m. The average values are reported as mean ± s.d. They were obtained from n = 3 independent SPR experiments.

The quality of the SPR fits was assessed using the reduced chi-squared (χ^2). χ^2 quantifies the deviation between the experimental and fitted sensorgrams. Lower χ^2 values indicate good agreement between the binding model and the acquired SPR data.

EGF & $^{DG}EGFR^{CHO\text{-}K1}$				
**Repeat**	k_{on} ($\times 10^5$ $M^{-1}s^{-1}$)	k_{off} ($\times 10^{-1}$ s^{-1})	K_D (nM)	χ^2
**1**	4.38 ± 0.02	3.06 ± 0.01	700	0.030
**2**	4.91 ± 0.02	2.94 ± 0.01	598	0.038
**3**	3.65 ± 0.75	2.80 ± 0.57	768	0.018
**Average**	4.31 ± 0.52	2.93 ± 0.11	689 ± 70	

TGF-α & $^{DG}EGFR^{CHO\text{-}K1}$				
**Repeat**	k_{on} ($\times 10^5$ $M^{-1}s^{-1}$)	k_{off} ($\times 10^{-1}$ s^{-1})	K_D (nM)	χ^2
**1**	6.09 ± 0.05	6.42 ± 0.05	1054	0.0255
**2**	5.47 ± 0.03	6.19 ± 0.03	1131	0.0161
**3**	5.47 ± 0.03	5.74 ± 0.03	1050	0.0204
**Average**	5.68 ± 0.29	6.12 ± 0.28	1078 ± 37	

HB-EGF & $^{DG}EGFR^{CHO\text{-}K1}$				
**Repeat**	k_{on} ($\times 10^5$ $M^{-1}s^{-1}$)	k_{off} ($\times 10^{-1}$ s^{-1})	K_D (nM)	χ^2
**1**	9.74 ± 0.02	1.72 ± 0.01	176	0.0348
**2**	6.20 ± 0.02	1.76 ± 0.01	283	0.0366
**3**	8.77 ± 0.03	1.70 ± 0.01	193	0.0495
**Average**	8.24 ± 1.49	1.72 ± 0.02	218 ± 47	

13. Kinetic and affinity constants of high-affinity GFs with EGFR^{Expi293F} using BLI and SPR.

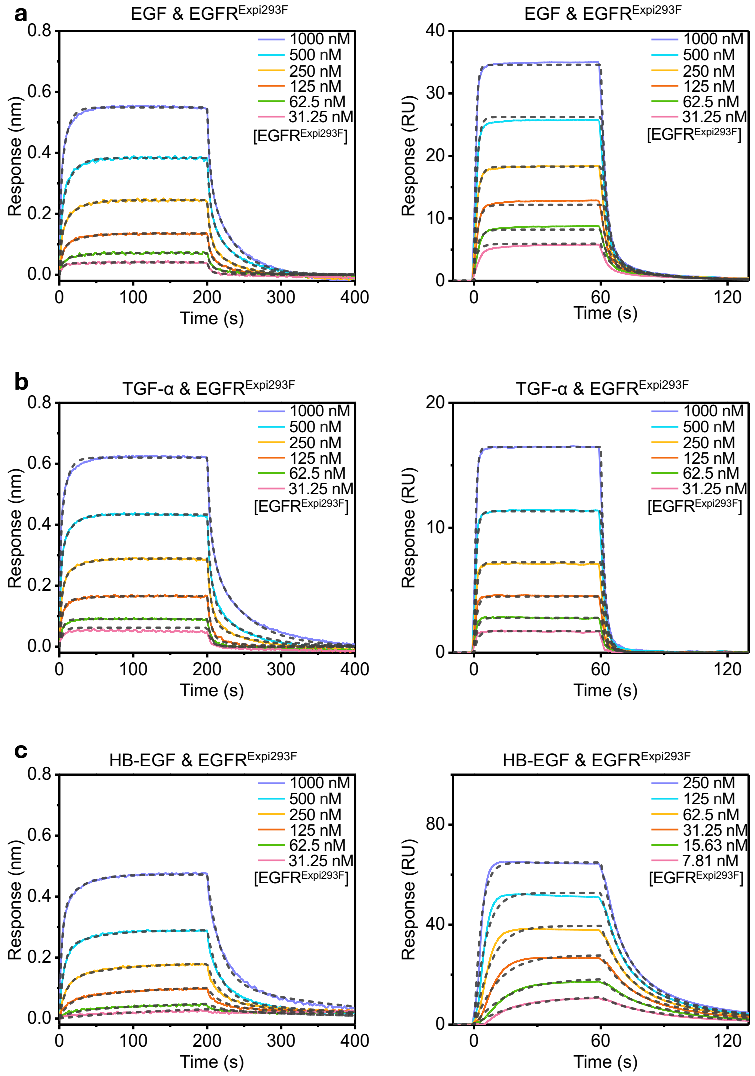

Supplementary Figure S15. BLI and SPR analyses of interactions between individual GFs with the extracellular domain of EGFR expressed in Expi293F cells. The binding interactions between GFs and the extracellular domain of EGFR expressed in Expi293F cells (EGFR^{Expi293F}) were measured by BLI and SPR. **(a)** EGF–EGFR^{Expi293F}, **(b)** TGF-α–EGFR^{Expi293F}, and **(c)** HB-EGF–EGFR^{Expi293F}. Representative BLI (left) and SPR (right) sensorgrams show the association and dissociation phases. For BLI, 10 nM biotinylated GFs were immobilized on streptavidin-coated biosensors for 10 min, followed by association with six twofold serial dilutions of EGFR^{Expi293F} and dissociation in GF-free buffer. For SPR, EGFR^{Expi293F} was immobilized on Cytiva CM5 sensor chips, and GFs were injected into the solution. All binding curves were globally fitted (black dotted lines) using a heterogeneous ligand 2:1 interaction model. For BLI experiments, the association phase is represented by the first 200 seconds, and the dissociation is indicated by the second 200 seconds. For SPR experiments, the association phase is represented by the first 60 seconds, and the dissociation is indicated by the second 70 seconds.

<u>Supplementary Table S23.</u> BLI-determined kinetic rate constants of association and dissociation, k_{on} and k_{off}, respectively, and equilibrium dissociation constants, K_D, of the GF-receptor interactions with the EGF, TGF-α, or HB-EGF attached to the BLI chip surface and the $EGFR^{Expi293F}$ added to the wells. The running buffer consisted of 20 mM Tris-HCl, 150 mM KCl, and 1 mg/ml bovine serum albumin (BSA), 0.005% (v/v) Tween 20, pH 7.5. Values of all constants, which were derived using a heterogeneous ligand 2:1 binding model and global fits **(Experimental Section)**, indicate mean ± s.e.m. The average values are reported as mean ± s.d. and were obtained from n = 3 independent BLI experiments.

The quality of the fits was assessed using the reduced chi-squared (χ^2) and the coefficient of determination (R-squared, R^2). χ^2 quantifies the deviation between the experimental and fitted sensorgrams, while R^2 indicates how well the model predicts the binding response. Lower χ^2 and higher R^2 values indicate good agreement between the binding model and the acquired BLI data.

**EGF & $EGFR^{Expi293F}$**								
**Repeat**	**k_{on-1} ($\times10^5$ $M^{-1}s^{-1}$)**	**k_{off-1} ($\times10^{-2}$ s^{-1})**	**K_{D-1} (nM)**	**k_{on-2} ($\times10^4$ $M^{-1}s^{-1}$)**	**k_{off-2} ($\times10^{-2}$ s^{-1})**	**K_{D-2} (nM)**	**χ^2**	**R^2**
**1**	3.10 ± 0.03	7.22 ± 0.04	233 ± 1	4.45 ± 0.06	1.23 ± 0.01	276 ± 4	0.0175	0.999
**2**	2.74 ± 0.04	6.51 ± 0.05	237 ± 1	3.73 ± 0.08	1.04 ± 0.01	278 ± 6	0.0415	0.999
**3**	2.65 ± 0.04	6.33 ± 0.05	239 ± 1	3.87 ± 0.10	1.17 ± 0.02	303 ± 9	0.0449	0.999
**Average**	2.83 ± 0.20	6.68 ± 0.39	236 ± 3	4.02 ± 0.31	1.15 ± 0.08	286 ± 12		

**TGF-α & $EGFR^{Expi293F}$**								
**Repeat**	**k_{on-1} ($\times10^5$ $M^{-1}s^{-1}$)**	**k_{off-1} ($\times10^{-2}$ s^{-1})**	**K_{D-1} (nM)**	**k_{on-2} ($\times10^4$ $M^{-1}s^{-1}$)**	**k_{off-2} ($\times10^{-2}$ s^{-1})**	**K_{D-2} (nM)**	**χ^2**	**R^2**
**1**	6.30 ± 0.40	18.6 ± 0.5	296 ± 3	10.9 ± 0.4	2.10 ± 0.04	193 ± 8	0.0496	0.995
**2**	4.88 ± 0.11	14.7 ± 0.1	302 ± 1	3.98 ± 0.07	0.978 ± 0.007	246 ± 4	0.0647	0.999
**3**	5.13 ± 0.11	13.3 ± 0.1	259 ± 1	3.66 ± 0.05	0.810 ± 0.006	221 ± 4	0.0411	0.999
**Average**	5.44 ± 0.62	15.5 ± 2.3	285 ± 19	6.17 ± 3.33	1.29 ± 0.57	220 ± 22		

**HB-EGF & $EGFR^{Expi293F}$**								
**Repeat**	**k_{on-1} ($\times10^5$ $M^{-1}s^{-1}$)**	**k_{off-1} ($\times10^{-2}$ s^{-1})**	**K_{D-1} (nM)**	**k_{on-2} ($\times10^4$ $M^{-1}s^{-1}$)**	**k_{off-2} ($\times10^{-2}$ s^{-1})**	**K_{D-2} (nM)**	**χ^2**	**R^2**
**1**	1.10 ± 0.03	6.11 ± 0.08	555 ± 4	1.35 ± 0.06	0.285 ± 0.014	211 ± 14	0.0537	0.997
**2**	1.10 ± 0.03	5.62 ± 0.08	513 ± 4	1.36 ± 0.06	0.350 ± 0.010	258 ± 14	0.0413	0.997
**3**	1.21 ± 0.03	5.57 ± 0.07	462 ± 3	1.42 ± 0.05	0.329 ± 0.008	231 ± 10	0.0718	0.998
**Average**	1.13 ± 0.05	5.76 ± 0.24	510 ± 38	1.38 ± 0.03	0.321 ± 0.027	233 ± 19		

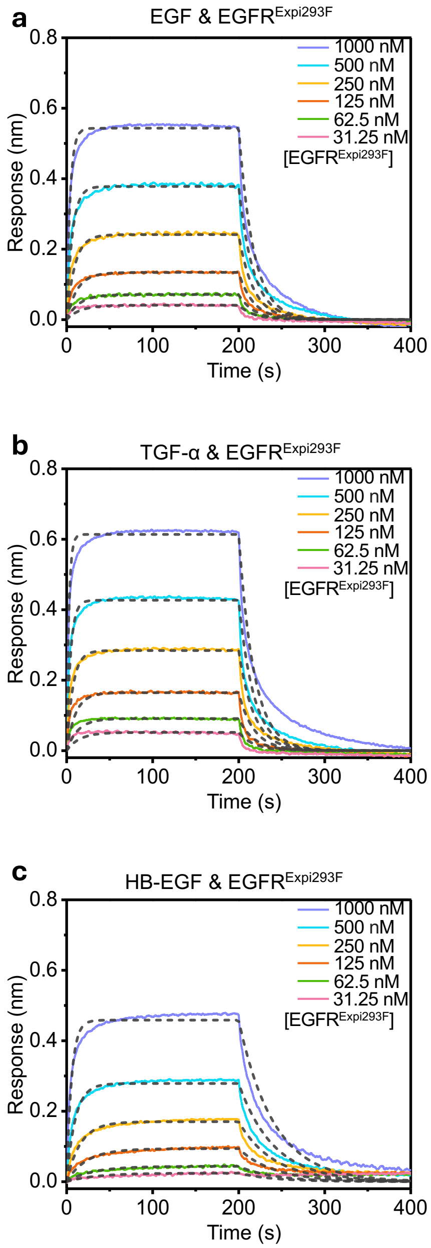

<u>Supplementary Figure S16.</u> BLI assays measuring interactions between GFs and the EGFR extracellular domain expressed by the Expi293F cell line. (a) EGF–EGFR^{Expi293F}. **(b)** TGF-α–EGFR^{Expi293F}. **(c)** HB-EGF–EGFR^{Expi293F}. Representative BLI sensorgrams show the association and dissociation phases. For each panel, 10 nM biotinylated GF was loaded onto streptavidin-loaded BLI sensors for 10 min, then dipped into buffers containing six twofold serial dilutions of EGFRExpi293F from 1000 nM to 31.25 nM for the association phase. Sensors were subsequently transferred to EGFR^{Expi293F}-free buffer for the dissociation phase. Dashed lines indicate fits of BLI sensorgrams showing interactions between EGFR^{Expi293F} and its high-affinity GFs. The binding curves were fitted (black dotted lines) using the FortéBio Octet Data Analysis software (FortéBio) with a standard 1:1 binding model and global fits. The association phase is represented by the first 200 seconds, and the dissociation is indicated by the second 200 seconds.

<u>Supplementary Table S24.</u> BLI-determined kinetic rate constants of association and dissociation, k_{on} and k_{off}, respectively, and equilibrium dissociation constants, K_D, of the GF-receptor interactions with EGF, TGF-α, or HB-EGF attached to the BLI sensor surface and EGFR^{Expi293F} added to the sensor wells. The running buffer consisted of 20 mM Tris-HCl, 150 mM KCl, and 1 mg/ml bovine serum albumin (BSA), 0.005% (v/v) Tween 20, pH 7.5. Values of all constants, which were derived using a standard 1:1 binding model and global fits **(Experimental Section)**, indicate mean ± s.e.m. The average values are reported as mean ± s.d. and were obtained from n = 3 independent BLI experiments.

The quality of the fits was assessed using the reduced chi-squared (χ^2) and the coefficient of determination (R-squared, R^2). χ^2 quantifies the deviation between the experimental and fitted sensorgrams, while R^2 indicates how well the model predicts the binding response. Lower χ^2 and higher R^2 values indicate good agreement between the binding model and the acquired BLI data.

		**EGF & EGFR^{Expi293F}**			
**Repeat**	k_{on} (×10^5 $M^{-1}s^{-1}$)	k_{off} (×10^{-2} s^{-1})	K_D (nM)	χ^2	R^2
**1**	1.77 ± 0.04	3.57 ± 0.02	202 ± 4	0.325	0.994
**2**	1.69 ± 0.03	3.41 ± 0.02	202 ± 4	0.410	0.993
**3**	1.66 ± 0.03	3.61 ± 0.02	217 ± 4	0.304	0.995
**Average**	1.71 ± 0.05	3.53 ± 0.08	207 ± 7		

		**TGF-α & EGFR^{Expi293F}**			
**Repeat**	k_{on} (×10^5 $M^{-1}s^{-1}$)	k_{off} (×10^{-2} s^{-1})	K_D (nM)	χ^2	R^2
**1**	3.25 ± 0.10	5.65 ± 0.06	174 ± 6	1.19	0.989
**2**	2.41 ± 0.09	3.93 ± 0.05	163 ± 6	1.23	0.981
**3**	2.47 ± 0.10	3.18 ± 0.04	129 ± 5	1.15	0.977
**Average**	2.71 ± 0.38	4.25 ± 1.03	155 ± 19		

		**HB-EGF & EGFR^{Expi293F}**			
**Repeat**	k_{on} (×10^5 $M^{-1}s^{-1}$)	k_{off} (×10^{-2} s^{-1})	K_D (nM)	χ^2	R^2
**1**	0.846 ± 0.032	1.60 ± 0.02	189 ± 7	0.827	0.957
**2**	0.769 ± 0.027	1.66 ± 0.02	216 ± 8	0.478	0.969
**3**	0.793 ± 0.024	1.47 ± 0.02	186 ± 6	0.872	0.970
**Average**	0.803 ± 0.032	1.58 ± 0.08	197 ± 14		

Supplementary Table S25. SPR-determined kinetic rate constants of association and dissociation, k_{on} and k_{off}, respectively, and equilibrium dissociation constants, K_D, of the GF-receptor interactions with $EGFR^{Expi293F}$ attached to the SPR chip surface and EGF, TGF-α, or HB-EGF added to the solution. The running buffer consisted of 20 mM Tris-HCl, 150 mM KCl, and 1 mg/ml bovine serum albumin (BSA), 0.05% Tween 20, pH 7.5. Values of all constants, which were derived using a heterogeneous ligand 2:1 binding model and global fits **(Experimental Section)**, indicate mean ± s.e.m. The average values are reported as mean ± s.d. and were obtained from n = 3 independent SPR experiments. For EGF and TGF-α, the substate "1" contributed less than 8% and 3%, respectively, to the total response. Hence, the kinetic and equilibrium constants for these cases were not determined (ND) with statistically significant accuracy.

The quality of the SPR fits was assessed using the reduced chi-squared (χ^2). χ^2 quantifies the deviation between the experimental and fitted sensorgrams. Lower χ^2 values indicate good agreement between the binding model and the acquired SPR data.

EGF & $EGFR^{Expi293F}$							
**Repeat**	k_{on-1} ($\times 10^6$ $M^{-1}s^{-1}$)	k_{off-1} ($\times 10^{-1}$ s^{-1})	K_{D-1} (nM)	k_{on-2} ($\times 10^5$ $M^{-1}s^{-1}$)	k_{off-2} ($\times 10^{-1}$ s^{-1})	K_{D-2} (nM)	χ^2
**1**	ND	ND	ND	4.83 ± 0.01	3.95 ± 0.01	817	0.027
**2**	ND	ND	ND	6.33 ± 0.03	3.88 ± 0.02	613	0.033
**3**	ND	ND	ND	5.07 ± 0.03	3.57 ± 0.02	704	0.130
**Average**	ND	ND	ND	5.41 ± 0.66	3.80 ± 0.17	711 ± 83	

TGF-α & $EGFR^{Expi293F}$							
**Repeat**	k_{on-1} ($\times 10^6$ $M^{-1}s^{-1}$)	k_{off-1} ($\times 10^{-1}$ s^{-1})	K_{D-1} (nM)	k_{on-2} ($\times 10^5$ $M^{-1}s^{-1}$)	k_{off-2} ($\times 10^{-1}$ s^{-1})	K_{D-2} (nM)	χ^2
**1**	ND	ND	ND	1.91 ± 0.04	7.28 ± 0.07	3820	0.0179
**2**	ND	ND	ND	2.48 ± 0.01	5.11 ± 0.01	2060	0.00466
**3**	ND	ND	ND	1.39 ± 0.01	5.27 ± 0.01	3780	0.00543
**Average**	ND	ND	ND	1.92 ± 0.44	5.89 ± 0.99	3230 ± 822	

HB-EGF & $EGFR^{Expi293F}$							
**Repeat**	k_{on-1} ($\times 10^6$ $M^{-1}s^{-1}$)	k_{off-1} ($\times 10^{-1}$ s^{-1})	K_{D-1} (nM)	k_{on-2} ($\times 10^5$ $M^{-1}s^{-1}$)	k_{off-2} ($\times 10^{-1}$ s^{-1})	K_{D-2} (nM)	χ^2
**1**	2.95 ± 0.03	0.232 ± 0.002	7.87	10.5 ± 0.1	1.09 ± 0.01	104	0.282
**2**	3.29 ± 0.02	0.256 ± 0.002	7.79	10.4 ± 0.1	1.25 ± 0.01	120	0.367
**3**	6.15 ± 0.12	0.283 ± 0.004	4.60	14.8 ± 0.1	1.28 ± 0.01	86.4	0.665
**Average**	4.13 ± 1.44	0.257 ± 0.021	6.75 ± 1.52	11.9 ± 2.0	1.20 ± 0.08	104 ± 14	

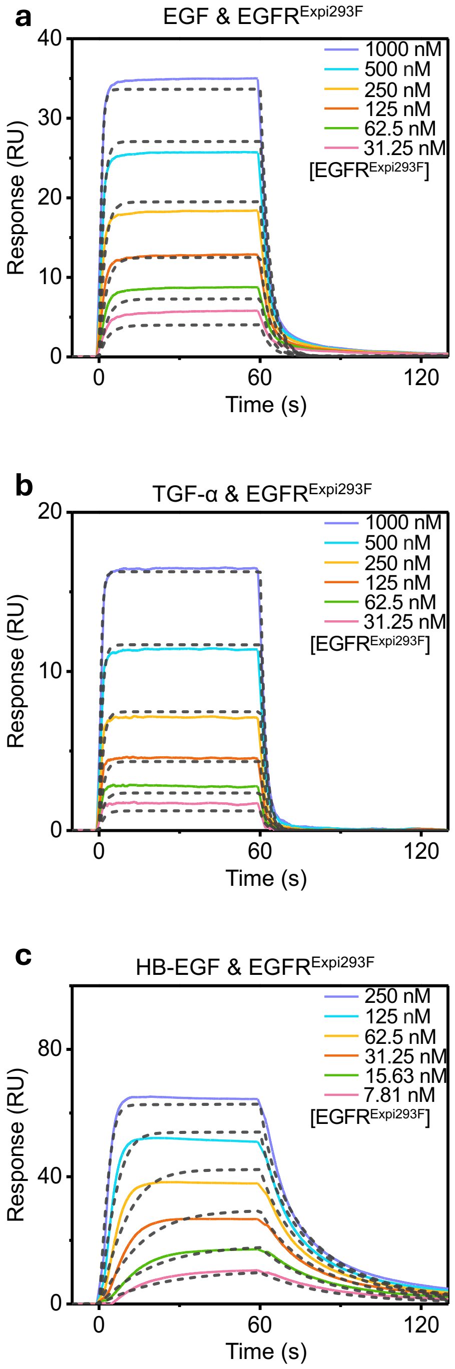

Supplementary Figure S17. SPR analyses of interactions between individual GFs with the extracellular domain of EGFR^{Expi293F}. (a) EGF–EGFR^{Expi293F}, **(b)** TGF-α–EGFR^{Expi293F}, and **(c)** HB-EGF– EGFR^{Expi293F}. Representative SPR sensorgrams show the association and dissociation phases. EGFR^{Expi293F} was immobilized on Cytiva CM5 sensor chips, and GFs were injected into solutions. All binding curves were globally fitted (black dotted lines) using a standard 1:1 binding interaction model. The association phase is represented by the first 60 seconds, and the dissociation is indicated by the second 70 seconds.

<u>Supplementary Table S26.</u> SPR-determined kinetic rate constants of association and dissociation, k_{on} and k_{off}, respectively, and equilibrium dissociation constants, K_D, of the GF-receptor interactions with the $EGFR^{Expi293F}$ attached to the SPR chip surface and the EGF, TGF-α, or HB-EGF added to the solution. The running buffer consisted of 20 mM Tris-HCl, 150 mM KCl, and 1 mg/ml bovine serum albumin (BSA), 0.05% Tween 20, pH 7.5. Values of all constants, which were derived using a standard 1:1 binding model and global fits **(Experimental Section)**, indicate mean ± s.e.m. The average values are reported as mean ± s.d. and were obtained from n = 3 independent SPR experiments.

The quality of the SPR fits was assessed using the reduced chi-squared (χ^2). χ^2 quantifies the deviation between the experimental and fitted sensorgrams. Lower χ^2 values indicate good agreement between the binding model and the acquired SPR data.

**EGF & $EGFR^{Expi293F}$**				
**Repeat**	k_{on} ($\times 10^5$ $M^{-1}s^{-1}$)	k_{off} ($\times 10^{-1}$ s^{-1})	K_D (nM)	χ^2
**1**	6.47 ± 0.03	2.55 ± 0.01	394	0.218
**2**	6.97 ± 0.03	2.53 ± 0.01	364	0.225
**3**	5.93 ± 0.03	2.61 ± 0.01	439	0.285
**Average**	6.46 ± 0.42	2.56 ± 0.03	399 ± 31	

**TGF-α & $EGFR^{Expi293F}$**				
**Repeat**	k_{on} ($\times 10^5$ $M^{-1}s^{-1}$)	k_{off} ($\times 10^{-1}$ s^{-1})	K_D (nM)	χ^2
**1**	9.18 ± 0.05	5.80 ± 0.03	632	0.0168
**2**	8.27 ± 0.03	4.99 ± 0.02	603	0.0192
**3**	7.38 ± 0.02	4.79 ± 0.01	649	0.01
**Average**	8.28 ± 0.73	5.19 ± 0.44	628 ± 19	

**HB-EGF & $EGFR^{Expi293F}$**				
**Repeat**	k_{on} ($\times 10^5$ $M^{-1}s^{-1}$)	k_{off} ($\times 10^{-1}$ s^{-1})	K_D (nM)	χ^2
**1**	25.7 ± 0.3	1.16 ± 0.01	45.1	0.220
**2**	33.3 ± 0.6	1.61 ± 0.03	48.4	0.926
**3**	16.3 ± 0.1	0.747 ± 0.003	46.0	0.681
**Average**	25.1 ± 7.0	1.17 ± 0.35	46.5 ± 1.4	

14. Kinetic and affinity constants of high-affinity GFs with EGFR^{GnTI} using BLI and SPR.

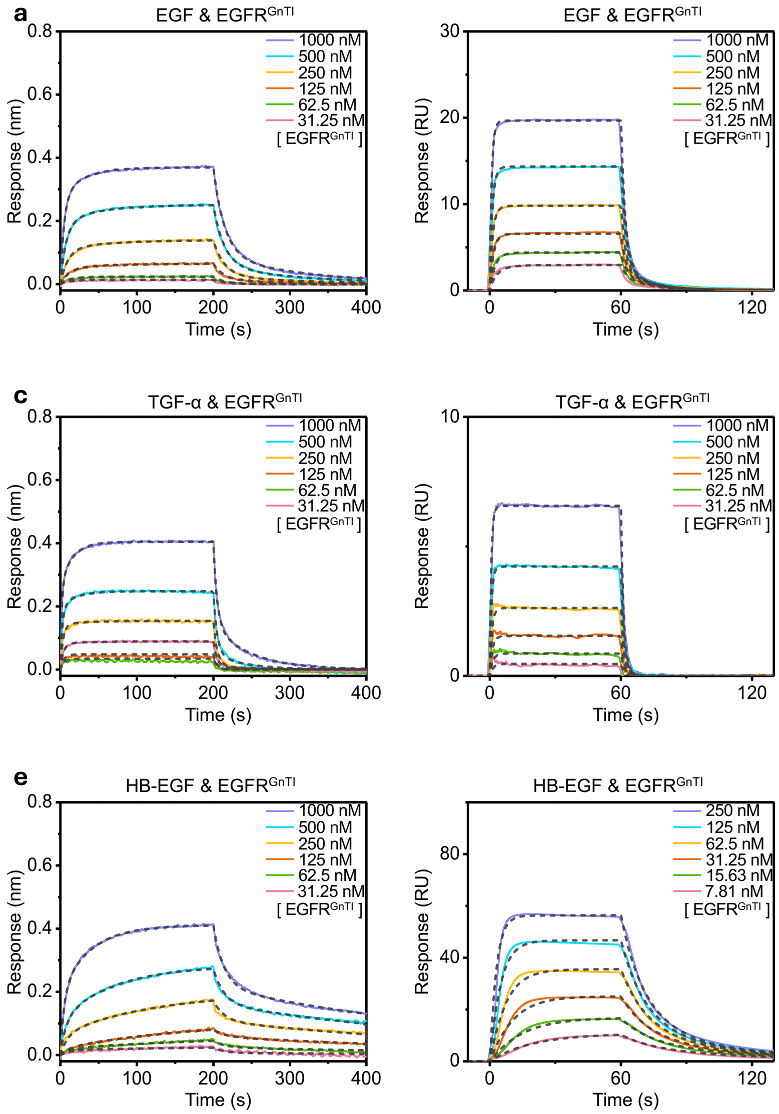

<u>Supplementary Figure S18.</u> **BLI and SPR analyses of interactions of GFs with the extracellular domain of EGFR expressed in GnTI cells.** Binding between GFs and the extracellular domain of EGFR expressed in GnTI cells (EGFR^{GnTI}) was measured by BLI and SPR. **(a)** EGF–EGFR^{GnTI}, **(b)** TGF-α– EGFR^{GnTI}, and **(c)** HB-EGF–EGFR^{GnTI}. Representative BLI (left) and SPR (right) sensorgrams show the association and dissociation phases. For BLI, 10 nM biotinylated growth factors were immobilized on streptavidin-coated biosensors for 10 min, followed by association with six twofold serial dilutions of EGFR^{GnTI} and dissociation in GF-free buffer. For SPR, EGFR^{GnTI} was immobilized on Cytiva CM5 sensor chips, and GFs were injected into the solution. All binding curves were globally fitted (black dotted lines) using a heterogeneous ligand 2:1 interaction model. For BLI experiments, the association phase is represented by the first 200 seconds, and the dissociation is indicated by the second 200 seconds. For SPR experiments, the association phase is represented by the first 60 seconds, and the dissociation is indicated by the second 70 seconds.

Supplementary Table S27. BLI-determined kinetic rate constants of association and dissociation, k_{on} and k_{off}, respectively, and equilibrium dissociation constants, K_D, of the GF-receptor interactions with EGF, TGF-α, or HB-EGF attached to the BLI chip surface and $EGFR^{GnTI}$ added to the wells. The running buffer consisted of 20 mM Tris-HCl, 150 mM KCl, and 1 mg/ml bovine serum albumin (BSA), 0.005% (v/v) Tween 20, pH 7.5. Values of all constants, which were derived using a heterogeneous ligand 2:1 binding model and global fits **(Experimental Section)**, indicate mean ± s.e.m. The average values are reported as mean ± s.d. and were obtained from n = 3 independent BLI experiments.

The quality of the fits was assessed using the reduced chi-squared (χ^2) and the coefficient of determination (R-squared, R^2). χ^2 quantifies the deviation between the experimental and fitted sensorgrams, while R^2 indicates how well the model predicts the binding response. Lower χ^2 and higher R^2 values indicate good agreement between the binding model and the acquired BLI data.

**EGF & $EGFR^{GnTI}$**								
**Repeat**	k_{on-1} ($\times 10^5$ $M^{-1}s^{-1}$)	k_{off-1} ($\times 10^{-2}$ s^{-1})	K_{D-1} (nM)	k_{on-2} ($\times 10^4$ $M^{-1}s^{-1}$)	k_{off-2} ($\times 10^{-2}$ s^{-1})	K_{D-2} (nM)	χ^2	R^2
**1**	1.70 ± 0.07	9.95 ± 0.23	585 ± 8	3.33 ± 0.12	1.89 ± 0.04	566 ± 23	0.0183	0.998
**2**	1.52 ± 0.05	7.85 ± 0.14	516 ± 5	3.00 ± 0.12	1.68 ± 0.04	559 ± 25	0.0516	0.998
**3**	1.40 ± 0.02	7.37 ± 0.04	526 ± 2	2.81 ± 0.04	1.01 ± 0.01	359 ± 7	0.0100	0.999
**Average**	1.54 ± 0.12	8.39 ± 1.12	542 ± 30	3.05 ± 0.22	1.52 ± 0.37	495 ± 96		

**TGF-α & $EGFR^{GnTI}$**								
**Repeat**	k_{on-1} ($\times 10^5$ $M^{-1}s^{-1}$)	k_{off-1} ($\times 10^{-2}$ s^{-1})	K_{D-1} (nM)	k_{on-2} ($\times 10^4$ $M^{-1}s^{-1}$)	k_{off-2} ($\times 10^{-2}$ s^{-1})	K_{D-2} (nM)	χ^2	R^2
**1**	3.69 ± 0.24	21.2 ± 0.6	574 ± 9	4.49 ± 0.22	2.13 ± 0.05	474 ± 26	0.0252	0.994
**2**	2.83 ± 0.14	20.5 ± 0.4	725 ± 10	2.75 ± 0.09	1.26 ± 0.02	458 ± 17	0.0485	0.998
**3**	2.81 ± 0.07	19.0 ± 0.2	677 ± 4	2.70 ± 0.05	1.00 ± 0.01	371 ± 7	0.0165	0.999
**Average**	3.11 ± 0.41	20.2 ± 0.9	659 ± 63	3.31 ± 0.83	1.46 ± 0.48	434 ± 45		

**HB-EGF & $EGFR^{GnTI}$**								
**Repeat**	k_{on-1} ($\times 10^5$ $M^{-1}s^{-1}$)	k_{off-1} ($\times 10^{-2}$ s^{-1})	K_{D-1} (nM)	k_{on-2} ($\times 10^4$ $M^{-1}s^{-1}$)	k_{off-2} ($\times 10^{-2}$ s^{-1})	K_{D-2} (nM)	χ^2	R^2
**1**	1.42 ± 0.04	8.00 ± 0.11	565 ± 4	2.65 ± 0.03	0.182 ± 0.003	276 ± 4	0.0213	0.998
**2**	1.37 ± 0.03	7.20 ± 0.09	527 ± 3	2.33 ± 0.02	0.255 ± 0.003	278 ± 6	0.0311	0.999
**3**	0.714 ± 0.034	5.35 ± 0.13	750 ± 13	0.911 ± 0.072	0.111 ± 0.011	303 ± 9	0.0170	0.991
**Average**	1.17 ± 0.32	6.85 ± 1.11	614 ± 97	1.97 ± 0.76	0.183 ± 0.059	286 ± 12		

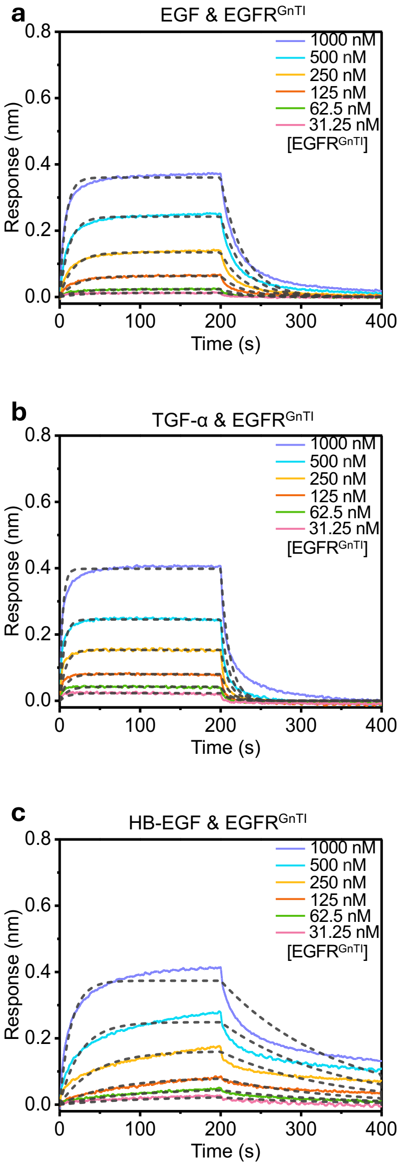

<u>Supplementary Figure S19.</u> BLI assays measuring the interactions of GFs with the EGFR extracellular domain expressed in the Expi293F GnTI cell line. (a) EGF–EGFR^{GnTI}. **(b)** TGF-α–EGFR^{GnTI}. **(c)** HB-EGF–EGFR^{GnTI}. Representative BLI sensorgrams show the association and dissociation phases. For each panel, 10 nM biotinylated GF was loaded onto streptavidin-coated sensors for 10 min, then dipped into buffers containing six twofold serial dilutions from 1000 nM to 31.25 nM of EGFR^{GnTI} for the association phase. Sensors were subsequently transferred to EGFR^{GnTI}-free buffer for the dissociation phase. Dashed lines indicate fits of BLI sensorgrams showing interactions between EGFR^{GnTI} and its high-affinity GFs. The binding curves were fitted (black dotted lines) using the ForteBio Octet Data Analysis software (ForteBio) with a standard 1:1 binding model. For these experiments, the association phase is represented by the first 200 seconds, and the dissociation is indicated by the second 200 seconds.

Supplementary Table S28. BLI-determined kinetic rate constants of association and dissociation, k_{on} and k_{off}, respectively, and equilibrium dissociation constants, K_D, of the GF-receptor interactions with EGF, TGF-α, or HB-EGF attached to the BLI chip surface and $EGFR^{GnTI}$ added to the wells. The running buffer consisted of 20 mM Tris-HCl, 150 mM KCl, and 1 mg/ml bovine serum albumin (BSA), 0.005% (v/v) Tween 20, pH 7.5. Values of all constants, which were derived using the standard 1:1 binding model and global fits **(Experimental Section)**, indicate mean ± s.e.m. The average values are reported as mean ± s.d. and were obtained from n = 3 independent BLI experiments.

The quality of the fits was assessed using the reduced chi-squared (χ^2) and the coefficient of determination (R-squared, R^2). χ^2 quantifies the deviation between the experimental and fitted sensorgrams, while R^2 indicates how well the model predicts the binding response. Lower χ^2 and higher R^2 values indicate good agreement between the binding model and the acquired BLI data.

			EGF & $EGFR^{GnTI}$		
**Repeat**	k_{on} ($\times 10^5$ $M^{-1}s^{-1}$)	k_{off} ($\times 10^{-2}$ s^{-1})	K_D (nM)	χ^2	R^2
**1**	0.883 ± 0.026	4.38 ± 0.04	496 ± 15	0.090	0.992
**2**	0.915 ± 0.018	4.24 ± 0.03	464 ± 9	0.129	0.996
**3**	0.981 ± 0.021	3.55 ± 0.03	362 ± 8	0.231	0.992
**Average**	0.926 ± 0.041	4.05 ± 0.36	440 ± 57		

			TGF-α & $EGFR^{GnTI}$		
**Repeat**	k_{on} ($\times 10^5$ $M^{-1}s^{-1}$)	k_{off} ($\times 10^{-2}$ s^{-1})	K_D (nM)	χ^2	R^2
**1**	1.80 ± 0.07	9.19 ± 0.13	511 ± 22	0.571	0.987
**2**	1.54 ± 0.07	5.93 ± 0.09	386 ± 18	0.343	0.982
**3**	1.59 ± 0.07	4.75 ± 0.07	299 ± 13	0.419	0.980
**Average**	1.64 ± 0.11	6.62 ± 1.88	399 ± 87		

			HB-EGF & $EGFR^{GnTI}$		
**Repeat**	k_{on} ($\times 10^5$ $M^{-1}s^{-1}$)	k_{off} ($\times 10^{-2}$ s^{-1})	K_D (nM)	χ^2	R^2
**1**	0.692 ± 0.021	0.623 ± 0.008	90.1 ± 3.0	0.627	0.948
**2**	0.643 ± 0.015	0.706 ± 0.007	110 ± 3	0.778	0.972
**3**	0.706 ± 0.032	0.862 ± 0.014	122 ± 6	0.262	0.909
**Average**	0.680 ± 0.027	0.730 ± 0.099	107 ± 13		

<u>Supplementary Table S29.</u> SPR-determined kinetic rate constants of association (k_{on}) and dissociation (k_{off}), along with the equilibrium dissociation constants (K_D) for GF-EGFR^{GnTI} receptor interactions. These interactions involve the EGFR^{GnTI} attached to the SPR chip surface and the addition of EGF, TGF-α, or HB-EGF to the solution. The running buffer consisted of 20 mM Tris-HCl, 150 mM KCl, 1 mg/ml bovine serum albumin (BSA), 0.05% Tween 20, at pH 7.5. All constant values, obtained using a heterogeneous ligand 2:1 binding model and global fits **(Experimental Section)**, are reported as mean ± s.e.m. The average values, derived from three independent SPR experiments, are reported as mean ± s.d. For EGF and TGF-α, the substrate "1" contributed less than 7% and 2%, respectively, to the total response. Hence, the kinetic and equilibrium constants for these cases were not determined (ND) with statistically significant accuracy.

The quality of the SPR fits was assessed using the reduced chi-squared (χ^2). χ^2 quantifies the deviation between the experimental and fitted sensorgrams. Lower χ^2 values indicate good agreement between the binding model and the acquired SPR data.

EGF & EGFR^{GnTI}

Repeat	k_{on-1} (×10^6 M^{-1}s^{-1})	k_{off-1} (×10^{-1} s^{-1})	K_{D-1} (nM)	k_{on-2} (×10^5 M^{-1}s^{-1})	k_{off-2} (×10^{-1} s^{-1})	K_{D-2} (nM)	χ^2
**1**	ND	ND	ND	4.07 ± 0.02	4.86 ± 0.02	1194	0.008
**2**	ND	ND	ND	5.51 ± 0.02	4.23 ± 0.01	767	0.014
**3**	ND	ND	ND	5.23 ± 0.02	4.88 ± 0.02	934	0.009
**Average**	ND	ND	ND	4.94 ± 0.62	4.66 ± 0.30	965 ± 176	

TGF-α & EGFR^{GnTI}

Repeat	k_{on-1} (×10^6 M^{-1}s^{-1})	k_{off-1} (×10^{-1} s^{-1})	K_{D-1} (nM)	k_{on-2} (×10^5 M^{-1}s^{-1})	k_{off-2} (×10^{-1} s^{-1})	K_{D-2} (nM)	χ^2
**1**	ND	ND	ND	3.52 ± 0.04	7.14 ± 0.07	2030	0.00703
**2**	ND	ND	ND	2.96 ± 0.03	6.57 ± 0.04	2220	0.00329
**3**	ND	ND	ND	3.54 ± 0.02	5.45 ± 0.02	1540	0.00503
**Average**	ND	ND	ND	3.34 ± 0.27	6.39 ± 0.70	1930 ± 287	

HB-EGF & EGFR^{GnTI}

Repeat	k_{on-1} (×10^6 M^{-1}s^{-1})	k_{off-1} (×10^{-1} s^{-1})	K_{D-1} (nM)	k_{on-2} (×10^5 M^{-1}s^{-1})	k_{off-2} (×10^{-1} s^{-1})	K_{D-2} (nM)	χ^2
**1**	3.83 ± 0.06	0.221 ± 0.002	5.79	11.8 ± 0.1	0.987 ± 0.005	83.7	0.241
**2**	7.82 ± 0.17	0.255 ± 0.003	3.27	11.5 ± 0.1	1.30 ± 0.01	113	0.182
**3**	2.90 ± 0.02	0.251 ± 0.002	8.65	10.1 ± 0.1	1.10 ± 0.01	109	0.368
**Average**	4.85 ± 2.14	0.242 ± 0.015	5.90 ± 2.20	11.1 ± 0.7	1.13 ± 0.13	102 ± 13	

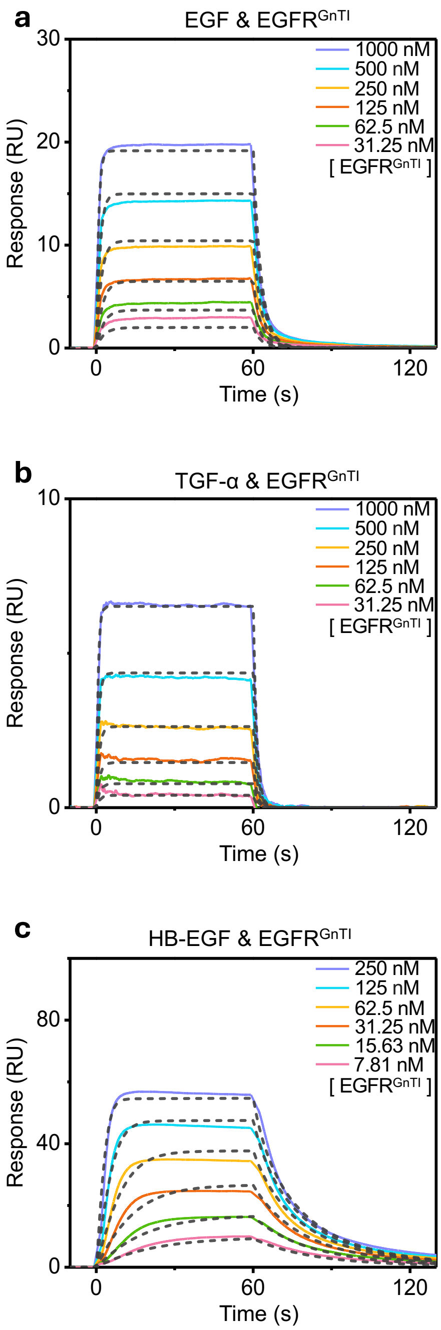

Supplementary Figure S20. SPR analyses of interactions of GFs with the extracellular domain of EGFR^{GnTI}. (a) EGF–EGFR^{GnTI}, **(b)** TGF-α–EGFR^{GnTI}, and **(c)** HB-EGF– EGFR^{GnTI}. Representative SPR sensorgrams show the association and dissociation phases. EGFR^{GnTI} was immobilized on Cytiva CM5 sensor chips, and GFs were injected into the solution. All binding curves were globally fitted (black dotted lines) using a standard 1:1 binding interaction model. For these experiments, the association phase is represented by the first 60 seconds, and the dissociation is indicated by the second 70 seconds.

<u>Supplementary Table S30.</u> SPR-determined kinetic rate constants of association and dissociation, k_{on} and k_{off}, respectively, and equilibrium dissociation constants, K_D, of the GF-receptor interactions with $EGFR^{GnTI}$ attached to the SPR chip surface and EGF, TGF-α, or HB-EGF added to the wells. The running buffer consisted of 20 mM Tris-HCl, 150 mM KCl, and 1 mg/ml bovine serum albumin (BSA), 0.05% Tween 20, pH 7.5. Values of all constants, which were derived using the standard 1:1 binding model and global fits **(Experimental Section)**, indicate mean ± s.e.m. The average values are reported as mean ± s.d. and were obtained from n = 3 independent SPR experiments.

The quality of the SPR fits was assessed using the reduced chi-squared (χ^2). χ^2 quantifies the deviation between the experimental and fitted sensorgrams. Lower χ^2 values indicate good agreement between the binding model and the acquired SPR data.

**EGF & $EGFR^{GnTI}$**				
**Repeat**	**k_{on} ($\times 10^5$ $M^{-1}s^{-1}$)**	**k_{off} ($\times 10^{-1}$ s^{-1})**	**K_D (nM)**	**χ^2**
**1**	6.29 ± 0.03	3.07 ± 0.01	488	0.051
**2**	6.70 ± 0.04	3.02 ± 0.01	451	0.087
**3**	6.82 ± 0.03	3.13 ± 0.01	459	0.063
**Average**	6.60 ± 0.23	3.07 ± 0.04	466 ± 16	

**TGF-α & $EGFR^{GnTI}$**				
**Repeat**	**k_{on} ($\times 10^5$ $M^{-1}s^{-1}$)**	**k_{off} ($\times 10^{-1}$ s^{-1})**	**K_D (nM)**	**χ^2**
**1**	6.67 ± 0.03	5.14 ± 0.02	771	0.0245
**2**	6.34 ± 0.02	4.90 ± 0.02	773	0.0185
**3**	6.42 ± 0.02	4.68 ± 0.02	728	0.0228
**Average**	6.48 ± 0.14	4.91 ± 0.19	757 ± 20	

**HB-EGF & $EGFR^{GnTI}$**				
**Repeat**	**k_{on} ($\times 10^5$ $M^{-1}s^{-1}$)**	**k_{off} ($\times 10^{-1}$ s^{-1})**	**K_D (nM)**	**χ^2**
**1**	19.2 ± 0.2	0.973 ± 0.008	50.8	0.981
**2**	28.1 ± 0.4	1.25 ± 0.02	44.5	0.687
**3**	14.1 ± 0.1	0.962 ± 0.003	68.1	0.751
**Average**	20.5 ± 5.8	1.06 ± 0.13	54.5 ± 10.0	

15. Two-dimensional plots of the kinetic and affinity constants of GF-EGFR ECD interactions for various EGFR ECD isoforms using BLI.

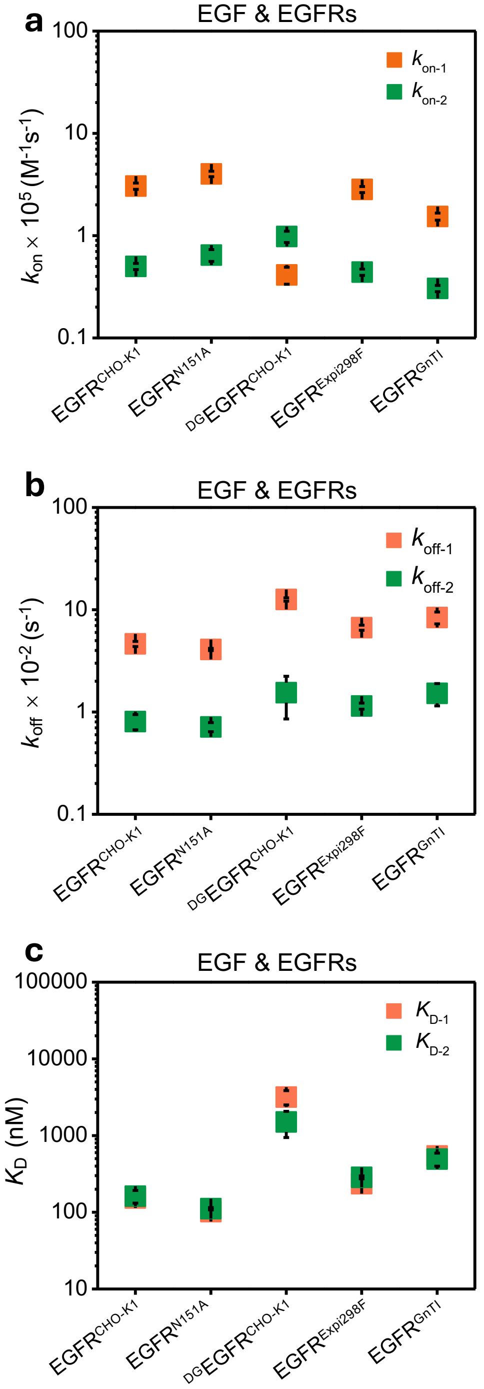

Supplementary Figure S21. 2D plots of the kinetic and affinity constants of GF-EGFR ECD interactions for various EGFR ECD isoforms using BLI. **(a)** The rate constants of association. **(b)** The rate constants of dissociation. **(c)** The equilibrium dissociation constants. Results indicate the values corresponding to a heterogeneous ligand 2:1 binding interaction model.

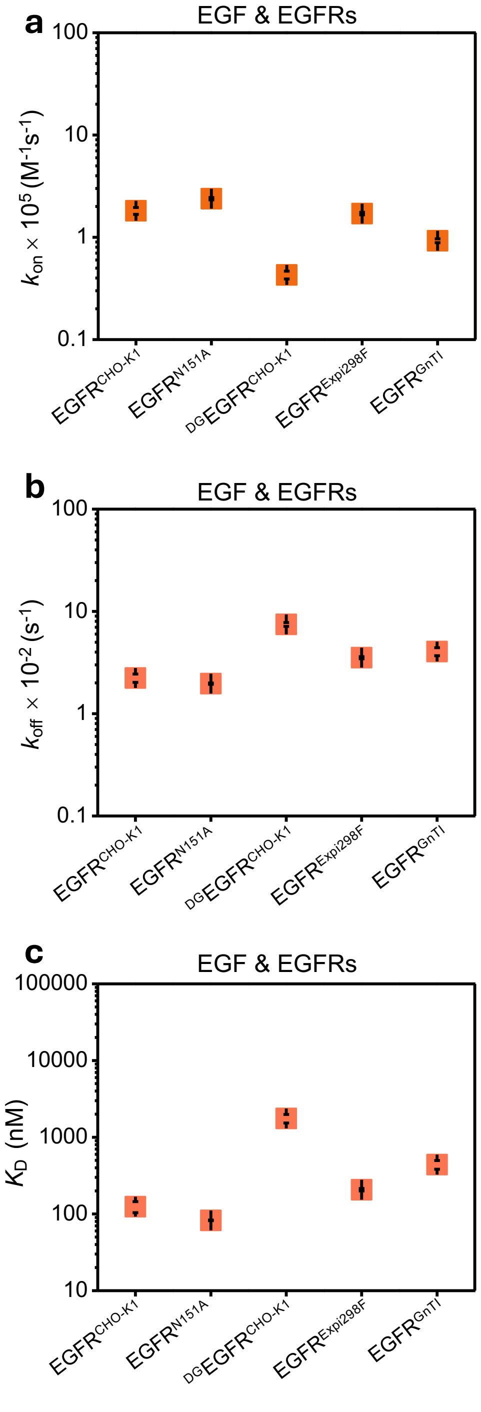

Supplementary Figure S22. 2D plots of the kinetic and affinity constants for GF-EGFR ECD interactions across various EGFR ECD isoforms using BLI. (a) The association rate constants. (b) The dissociation rate constants. **(c)** The equilibrium dissociation constants. Results are derived for the standard 1:1 binding model.

16. Two-dimensional plots of the kinetic and affinity constants of GF-EGFR ECD interactions for various high-affinity GFs against specific EGFR ECD isoforms using BLI.

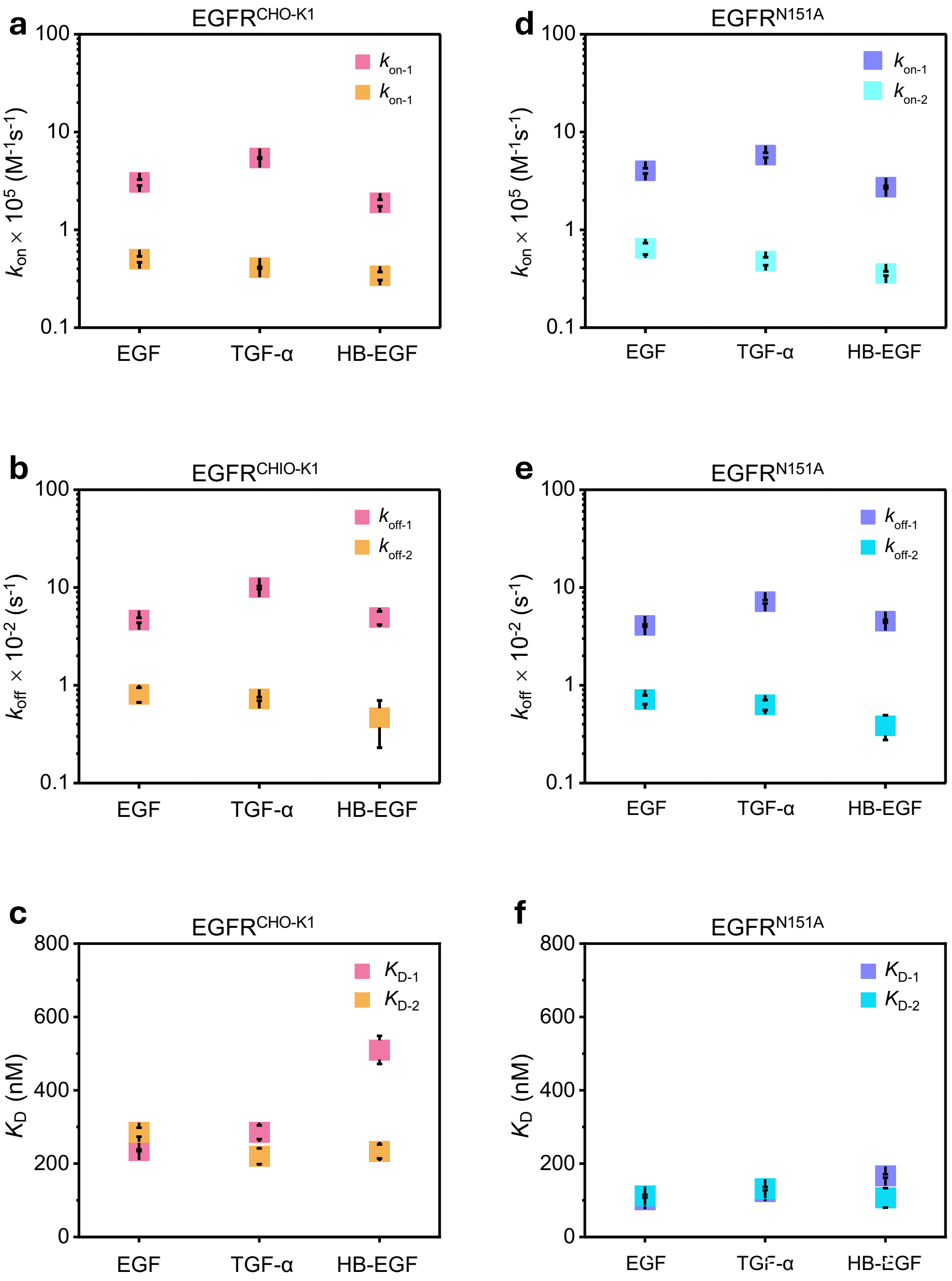

<u>Supplementary Figure S23.</u> 2D plots of the kinetic and affinity constants for GF-EGFR interactions with EGFR$^{CHO\text{-}K1}$ and EGFR^{N151A} isoforms using high-affinity GFs and BLI. Results show values derived with a heterogeneous ligand 2:1 binding interaction model.

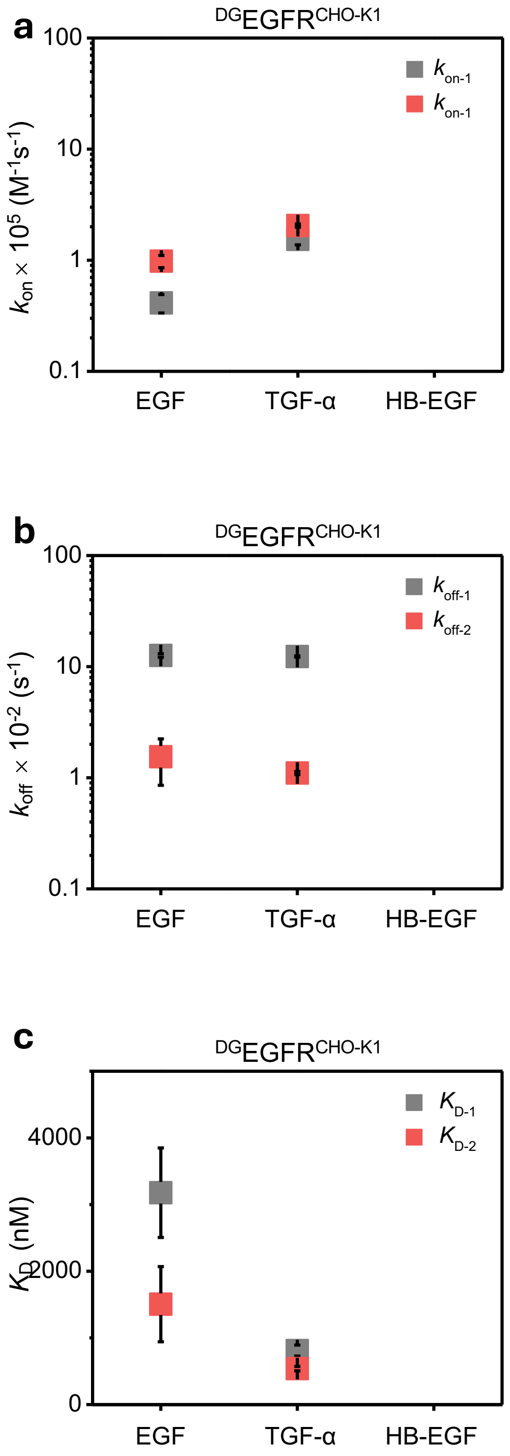

<u>Supplementary Figure S24.</u> 2D plots of the kinetic and affinity constants of GF-EGFR ECD interactions for $^{DG}EGFR^{CHO\text{-}K1}$ isoform against high-affinity GFs using BLI. Results indicate the values corresponding to a heterogeneous ligand 2:1 binding interaction model.

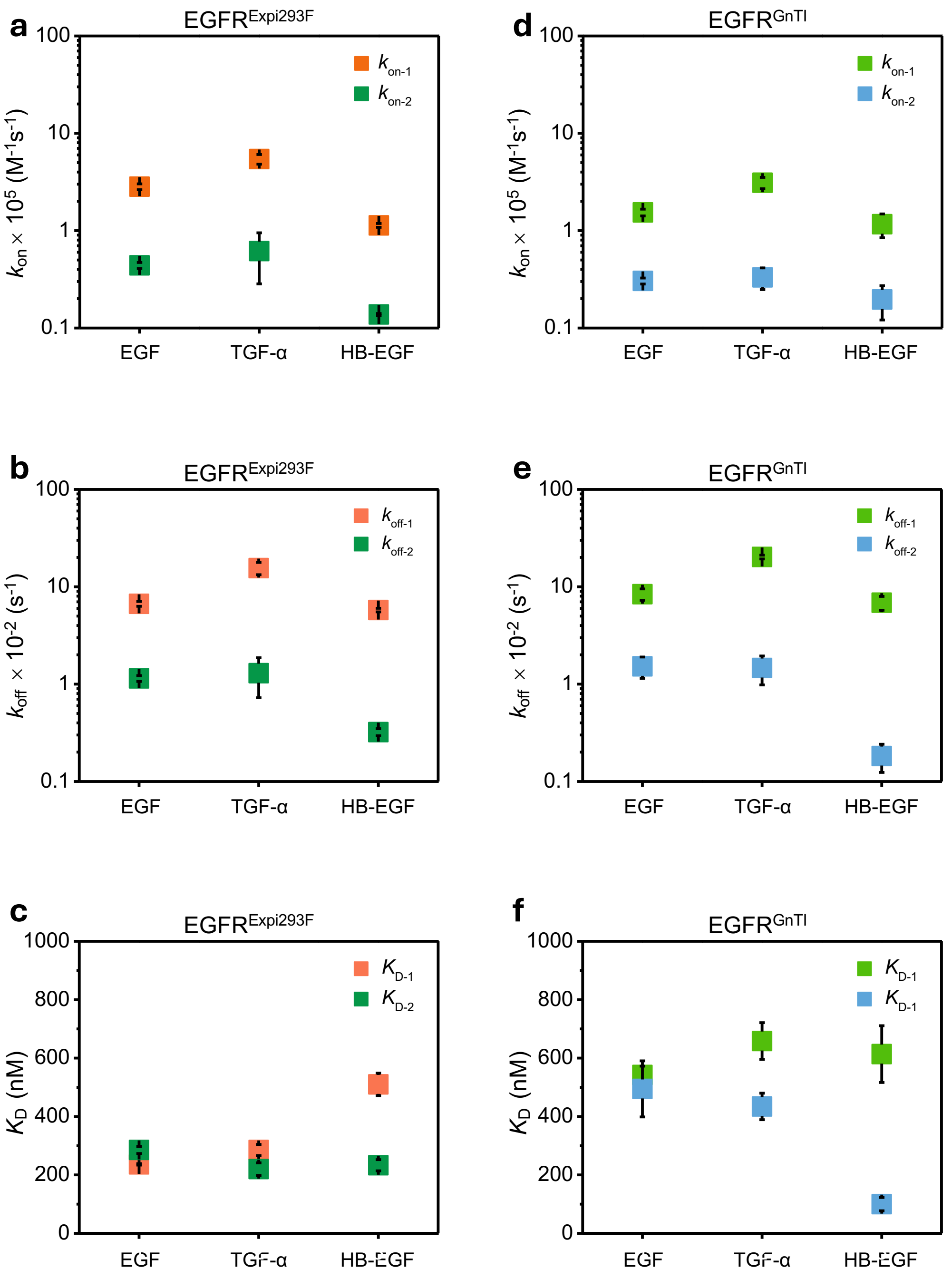

<u>Supplementary Figure S25.</u> 2D plots of the kinetic and affinity constants of GF-EGFR ECD interactions for $EGFR^{Expi293F}$ and $EGFR^{GnTI}$ isoforms against high-affinity GFs using BLI. Results indicate the values corresponding to a heterogeneous ligand 2:1 binding interaction model.

17. SUPPLEMENTARY REFERENCES

(1) Lu, H. S.; Chai, J. J.; Li, M.; Huang, B. R.; He, C. H.; Bi, R. C. Crystal structure of human epidermal growth factor and its dimerization. *J Biol Chem* **2001**, *276* (37), 34913-34917. DOI: 10.1074/jbc.M102874200 From NLM.

(2) Harvey, T. S.; Wilkinson, A. J.; Tappin, M. J.; Cooke, R. M.; Campbell, I. D. The solution structure of human transforming growth factor alpha. *Eur J Biochem* **1991**, *198* (3), 555-562. DOI: 10.1111/j.1432-1033.1991.tb16050.x From NLM.

(3) Louie, G. V.; Yang, W.; Bowman, M. E.; Choe, S. Crystal structure of the complex of diphtheria toxin with an extracellular fragment of its receptor. *Mol Cell* **1997**, *1* (1), 67-78. DOI: 10.1016/s1097-2765(00)80008-8 From NLM.

(4) Bai, X.; Sun, P.; Wang, X.; Long, C.; Liao, S.; Dang, S.; Zhuang, S.; Du, Y.; Zhang, X.; Li, N.; et al. Structure and dynamics of the EGFR/HER2 heterodimer. *Cell Discov* **2023**, *9* (1), 18. DOI: 10.1038/s41421-023-00523-5 From NLM.

(5) Huang, Y.; Ognjenovic, J.; Karandur, D.; Miller, K.; Merk, A.; Subramaniam, S.; Kuriyan, J. A molecular mechanism for the generation of ligand-dependent differential outputs by the epidermal growth factor receptor. *Elife* **2021**, *10*. DOI: 10.7554/eLife.73218 From NLM.

(6) Abramson, J.; Adler, J.; Dunger, J.; Evans, R.; Green, T.; Pritzel, A.; Ronneberger, O.; Willmore, L.; Ballard, A. J.; Bambrick, J.; et al. Accurate structure prediction of biomolecular interactions with AlphaFold 3. *Nature* **2024**, *630* (8016), 493-500. DOI: 10.1038/s41586-024-07487-w From NLM.

(7) Varki, A.; Cummings, R. D.; Aebi, M.; Packer, N. H.; Seeberger, P. H.; Esko, J. D.; Stanley, P.; Hart, G.; Darvill, A.; Kinoshita, T.; et al. Symbol Nomenclature for Graphical Representations of Glycans. *Glycobiology* **2015**, *25* (12), 1323-1324. DOI: 10.1093/glycob/cwv091 From NLM.

(8) Ives, C. M.; Singh, O.; D'Andrea, S.; Fogarty, C. A.; Harbison, A. M.; Satheesan, A.; Tropea, B.; Fadda, E. Restoring protein glycosylation with GlycoShape. *Nat Methods* **2024**, *21* (11), 2117-2127. DOI: 10.1038/s41592-024-02464-7 From NLM.

(9) Hasegawa, Y.; Takahashi, M.; Ariki, S.; Asakawa, D.; Tajiri, M.; Wada, Y.; Yamaguchi, Y.; Nishitani, C.; Takamiya, R.; Saito, A.; et al. Surfactant protein D suppresses lung cancer progression by downregulation of epidermal growth factor signaling. *Oncogene* **2015**, *34* (7), 838-845. DOI: 10.1038/onc.2014.20 From NLM.

(10) Abaandou, L.; Quan, D.; Shiloach, J. Affecting HEK293 Cell Growth and Production Performance by Modifying the Expression of Specific Genes. *Cells* **2021**, *10* (7). DOI: 10.3390/cells10071667 From NLM.

(11) Lim, C. Y.; Owens, N. A.; Wampler, R. D.; Ying, Y.; Granger, J. H.; Porter, M. D.; Takahashi, M.; Shimazu, K. Succinimidyl ester surface chemistry: implications of the

competition between aminolysis and hydrolysis on covalent protein immobilization. *Langmuir* **2014**, *30* (43), 12868-12878. DOI: 10.1021/la503439g From NLM.

(12) Kuo, W. T.; Lin, W. C.; Chang, K. C.; Huang, J. Y.; Yen, K. C.; Young, I. C.; Sun, Y. J.; Lin, F. H. Quantitative analysis of ligand-EGFR interactions: a platform for screening targeting molecules. *PLoS One* **2015**, *10* (2), e0116610. DOI: 10.1371/journal.pone.0116610 From NLM.

(13) Lahti, J. L.; Lui, B. H.; Beck, S. E.; Lee, S. S.; Ly, D. P.; Longaker, M. T.; Yang, G. P.; Cochran, J. R. Engineered epidermal growth factor mutants with faster binding on-rates correlate with enhanced receptor activation. *FEBS Lett* **2011**, *585* (8), 1135-1139. DOI: 10.1016/j.febslet.2011.03.044 From NLM.

(14) Zhou, M.; Felder, S.; Rubinstein, M.; Hurwitz, D. R.; Ullrich, A.; Lax, I.; Schlessinger, J. Real-time measurements of kinetics of EGF binding to soluble EGF receptor monomers and dimers support the dimerization model for receptor activation. *Biochemistry* **1993**, *32* (32), 8193-8198. DOI: 10.1021/bi00083a020 From NLM.

(15) Domagala, T.; Konstantopoulos, N.; Smyth, F.; Jorissen, R. N.; Fabri, L.; Geleick, D.; Lax, I.; Schlessinger, J.; Sawyer, W.; Howlett, G. J.; et al. Stoichiometry, kinetic and binding analysis of the interaction between epidermal growth factor (EGF) and the extracellular domain of the EGF receptor. *Growth Factors* **2000**, *18* (1), 11-29. DOI: 10.3109/08977190009003231 From NLM.

(16) De Crescenzo, G.; Grothe, S.; Lortie, R.; Debanne, M. T.; O'Connor-McCourt, M. Real-time kinetic studies on the interaction of transforming growth factor alpha with the epidermal growth factor receptor extracellular domain reveal a conformational change model. *Biochemistry* **2000**, *39* (31), 9466-9476. DOI: 10.1021/bi992987r From NLM.

(17) Miller, M. A.; Moss, M. L.; Powell, G.; Petrovich, R.; Edwards, L.; Meyer, A. S.; Griffith, L. G.; Lauffenburger, D. A. Targeting autocrine HB-EGF signaling with specific ADAM12 inhibition using recombinant ADAM12 prodomain. *Sci Rep* **2015**, *5*, 15150. DOI: 10.1038/srep15150 From NLM.

(18) Sanders, J. M.; Wampole, M. E.; Thakur, M. L.; Wickstrom, E. Molecular determinants of epidermal growth factor binding: a molecular dynamics study. *PLoS One* **2013**, *8* (1), e54136. DOI: 10.1371/journal.pone.0054136 From NLM.

(19) Jones, J. T.; Akita, R. W.; Sliwkowski, M. X. Binding specificities and affinities of egf domains for ErbB receptors. *FEBS Lett* **1999**, *447* (2-3), 227-231. DOI: 10.1016/s0014-5793(99)00283-5 From NLM.

(20) Ogiso, H.; Ishitani, R.; Nureki, O.; Fukai, S.; Yamanaka, M.; Kim, J. H.; Saito, K.; Sakamoto, A.; Inoue, M.; Shirouzu, M.; et al. Crystal structure of the complex of human epidermal growth factor and receptor extracellular domains. *Cell* **2002**, *110* (6), 775-787. DOI: 10.1016/s0092-8674(02)00963-7 From NLM.
